# Application of Lithiation–Borylation to the Total Synthesis of (−)-Rakicidin F

**DOI:** 10.1021/acs.orglett.2c03716

**Published:** 2022-12-20

**Authors:** Christian P. Bold, Kay Yeung, Felix Pape, Daniel Kaiser, Varinder K. Aggarwal

**Affiliations:** School of Chemistry, University of Bristol, Bristol BS8 1TS, U.K.

## Abstract

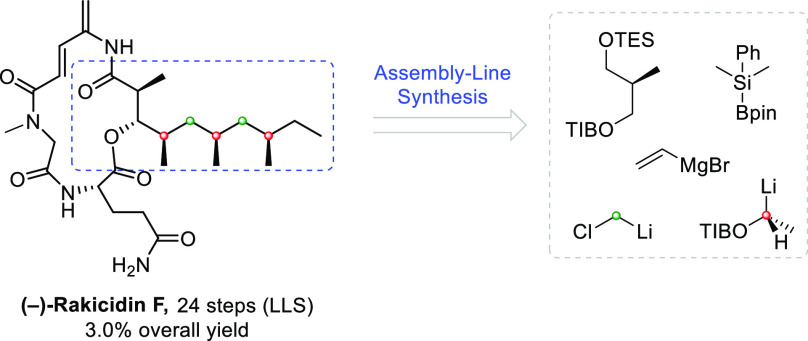

The stereochemistry of the lipophilic side chain of (+)-rakicidin F had not been determined until recently. Using our lithiation–borylation methodology (“assembly line synthesis”) we were able to efficiently prepare the all-syn isomer as well as the C-21 epimer of the side chain, and comparison with the natural product suggested that the natural product had all-syn stereochemistry. Completion of the total synthesis using a macrolactamization of the northern amide enabled us to confirm Wang and Chen’s stereochemical findings for the structure of (+)-rakicidin F.

Natural products continue to be a source of inspiration for the development of new therapeutic agents, highlighted by the 2015 Nobel Prize in Physiology or Medicine for the discovery and use of avermectins and artemisinin.^1^ In this context, the rakicidin family of natural products^2−7^ show promise as potential therapeutic agents, since they have been found to exhibit significant cytotoxicity against cancer stem cells and enhanced inhibitory activities toward cancer cells cultured in hypoxia, compared to those in normoxia.^2,3,8−13^ These unique cyclic depsipeptides all feature a 4-amino-2,4-pentadienoate (APD, marked in blue, Figure 1) moiety, a feature which is implicated in their biological activity. Prior to the outset of this work, the structures of all members of the rakicidins had been established except for those of rakicidin C and F. While the absolute stereochemistry of the core could potentially be extrapolated by analogy with other members of the class (although it turned out to be opposite), the stereochemistry of the lipophilic side chain could not. We sought to address this problem through synthesis and in particular using our lithiation–borylation methodology (“assembly line synthesis”),^14,15^ which had been successfully employed in establishing the stereochemistry of methyl substituted stereocenters on the alkyl chain of the baulamycins.^16^ During the course of this work, Wang and Chen published the synthesis and structural assignment of both rakicidin C and F.^17,18^ Here, the lipophilic side chains were built up by auxiliary-assisted asymmetric alkylations and Mukaiyama aldol reaction. In addition, they established that the absolute stereochemistry of rakicidin C and F were opposite to the other members of this class, being composed of the unnatural (*R*)-glutamine. In this paper we describe the total synthesis of (−)-rakicidin F, the most complex member of the family, confirming the stereochemistry established by Wang and Chen. Furthermore, our route differs from Wang and Chen’s route in that it uses a different macrolactamization strategy.

**Figure 1:**
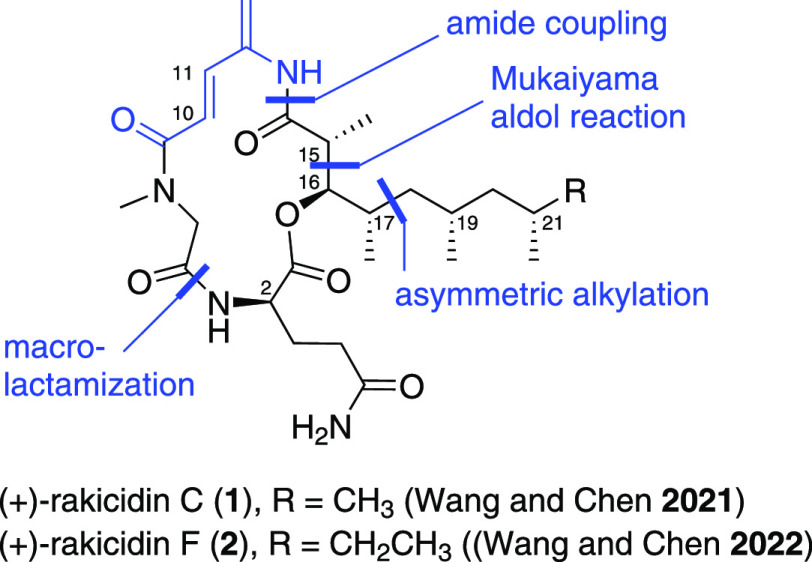
Structure of rakicidins C and F and Wang and Chen’s synthetic strategy.

There is strong evidence that the lipid chain in the family of rakicidins is highly influential to their biological activity, as only a small structural change of an additional methylene group within the lipid chain can result in varying potency levels of cytotoxic activity between rakicidin A, B, and E.^2,3,5,19^ For example, rakicidin A was found to possess a roughly 5-fold increased cytotoxicity in comparison to rakicidin B, which bears only a single additional methylene unit in its lipophilic side chain.^2^ For structure–activity relationship studies on the impact of different lipophilic side chains, it would be helpful to bring the side chain in at the end of the synthesis. However, this would require esterification or a macrolactonization of the hindered alcohol **5** (Scheme 1), which in early studies in our laboratory was found to be inefficient, and remains a notable challenge in synthesis.^20^ This meant that the hindered alcohol **5** had to be coupled to a single amino acid **10** rather than a more hindered peptide **4** and that, for ring closure, a macrolactamization had to be employed. Following this reasoning, our retrosynthetic analysis is shown in Scheme 1. Rakicidin F (**3**) could be obtained from acid **6** and amine **7** through amide coupling and macrolactamization. Acid **6** could be derived from aldehyde **8** and phosphonate **9** through Horner–Wadsworth–Emmons (HWE)-olefination and amine **7** through esterification of alcohol **5** and glutamine derivative **10**. Compound **5**, bearing the lipophilic side chain, could be constructed using our lithiation–borylation methodology starting from (*S*)-Roche ester **16**.

**Scheme 1:**
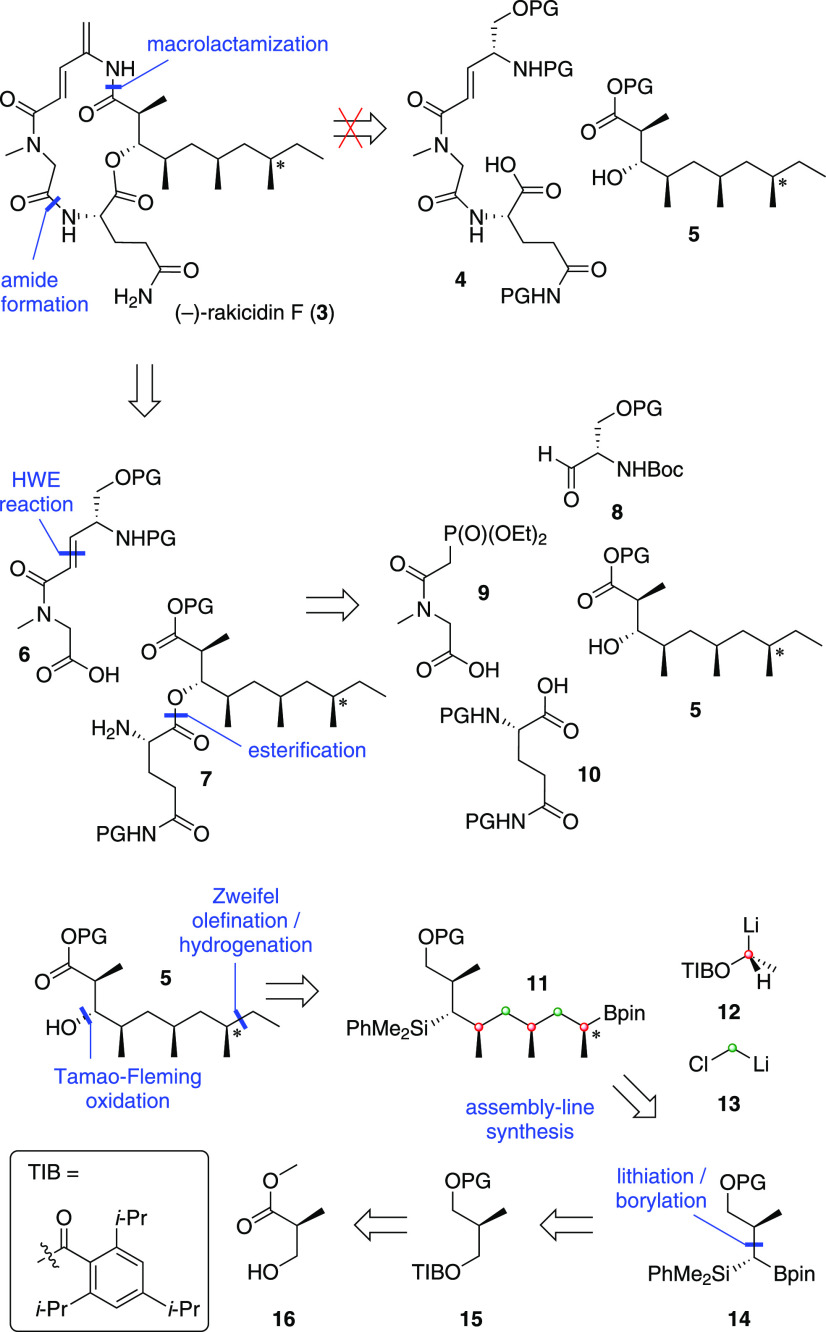
Retrosynthetic Analysis of (−)-Rakicidin F

We commenced our convergent synthesis of rakicidin F with the construction of fragment **19** from readily available Boc-protected l-serine methyl ester **17**. There is only one reported synthesis of aldehyde **19**, which is very low yielding,^21^ but gratifyingly we found that PMB-protection of the primary alcohol in **17** followed by reduction of the methyl ester to the alcohol,^22,23^ and oxidation to the aldehyde gave fragment **19** in high yield (83% over 3 steps) (Scheme 2). The second building block **20**(9) was readily synthesized from sarcosine methyl ester through an acylation and Arbuzov reaction. Phosphonate **20** and aldehyde **19** smoothly underwent an HWE-olefination with Ba(OH)_2_,^25^ giving a 6:1 mixture of *E*/*Z* isomers from which the pure *E* isomer could be isolated in 65% yield. A range of alternative methods were examined for this transformation (see Supporting Information (SI)), but they proved less effective (e.g., Masamune–Roush^26^). Finally, treatment with LiOH yielded lithium carboxylate **21**.

**Scheme 2:**
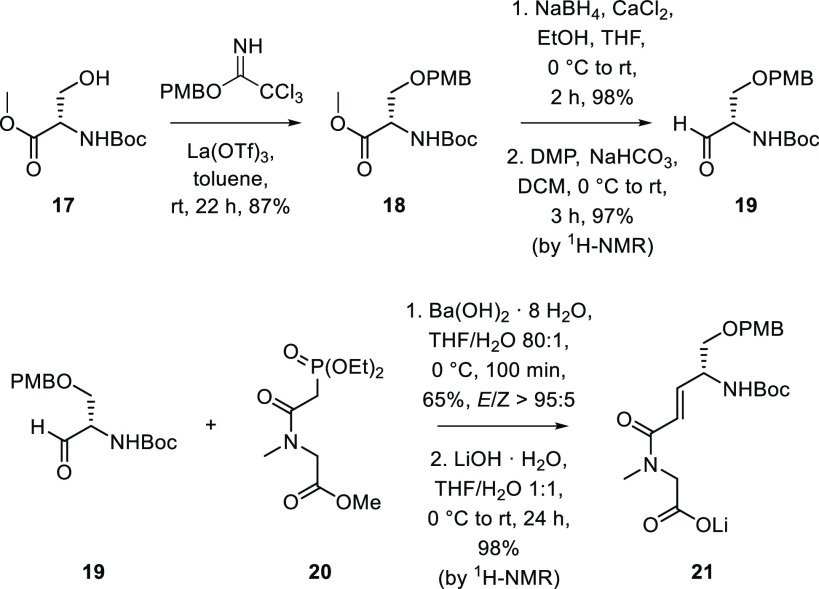
Synthesis of Carboxylate **21**

We then turned to the construction of fragment **31** using our assembly line synthesis strategy (Scheme 3).^27,28^ Benzoate ester **23** could be prepared on a multigram scale from commercially available (*S*)-Roche ester **16** in three steps. Lithiation–borylation was then employed to convert benzoate ester **23** into boronic ester **24** using conditions previously reported by our group.^29^ This gave **24** as a single diastereomer, with stereochemistry being controlled by the choice of enantiomer of sparteine used in the lithiation step. Gratifyingly, no mis-matched effects were observed. With boronic ester **24** in hand, we used five consecutive lithiation–borylation reactions (assembly line methodology) to grow the lipophilic side chain of rakicidin F in an iterative fashion with complete stereocontrol. The first homologation is illustrative. Treatment of boronic ester **24** with *in situ* generated lithiated species **12** formed boronate complex **26**, and upon warming to ambient temperature, a stereospecific 1,2-metalate rearrangement occurred to yield a secondary boronic ester, which overall resulted in the introduction of a methyl group on the homologated alkyl chain. The resulting boronic ester could undergo further homologation reactions with lithiated species **13** and **12** in a sequential fashion to grow the lipophilic side chain to the desired length with exquisite reagent-mediated stereocontrol. Zweifel olefination^30^ of boronic ester **28** afforded terminal alkene **29** in 49% yield over 6 steps to complete the synthesis of the lipophilic side chain. Fragment **31** was then accessed from alkene **29** in 5 steps. Initially, hydrogenation of the double bond was found to be problematic, giving a mixture of diastereoisomers using Pd/C, presumably due to competing alkene migration followed by hydrogenation.^31−34^ In contrast, using PtO_2_, no isomerization occurred.^31^ Subsequent Tamao–Fleming oxidation under Woerpel’s conditions^36^ with simultaneous TES deprotection afforded diol **30** in 92% over 2 steps. Finally, oxidation followed by allylation smoothly yielded fragment **31**.

**Scheme 3:**
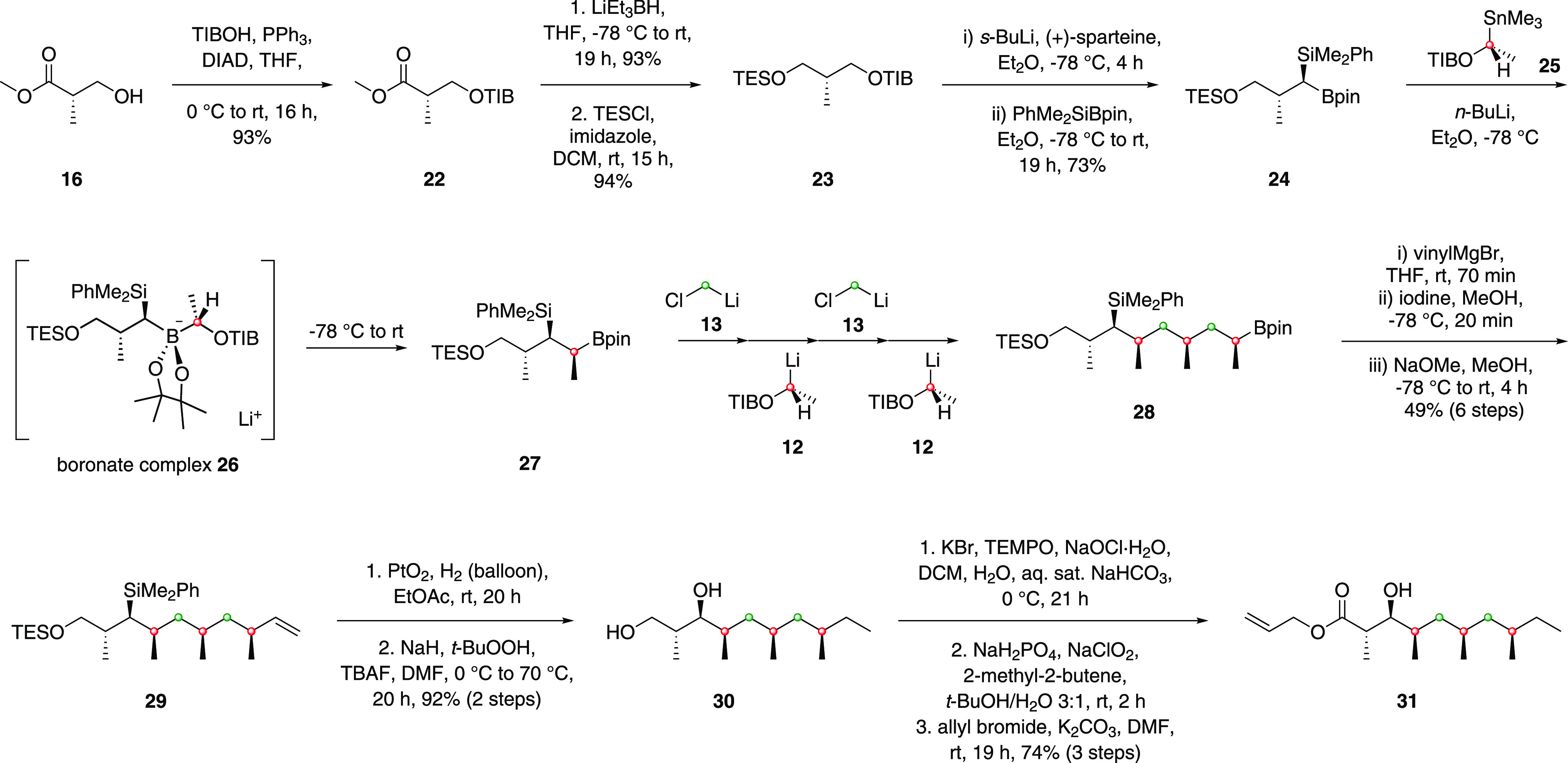
Synthesis of Side Chain Fragment **31** Using Lithiation–Borylation Methodology

In order to determine the relative stereochemistry of C-21 of the side chain of rakicidin F,^37^ we had planned to prepare its epimer (denoted as epi-**30** in Figure 2) using our lithiation–borylation methodology, but using the enantiomeric coupling partner in the final homologation. However, this was deemed unnecessary since the Pd/C hydrogenation followed by Tamao–Fleming oxidation had given a mixture of syn and anti diastereoisomers, while PtO_2_ had given a single syn isomer **30**. Comparison of the ^13^C NMR spectrum of the syn and anti isomers of the diols with that of the natural product^6^ gave a near identical match to the syn-configuration of the methyl groups in the side chain (Figure 2), suggesting that the all-syn configuration was the most likely stereochemistry of the side chain of rakicidin F.

**Figure 2:**
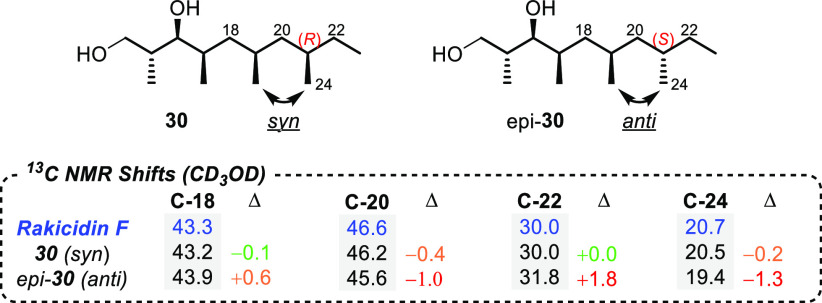
Comparison of the most significant ^13^C NMR data of rakicidin F with syn and anti lipophilic side chains **30** and its C-21 epimer epi-**30**. For a full comparison of the data, please see the SI.

Having evidence for the stereochemistry of the side chain, we continued the total synthesis of rakicidin F with added confidence. Coupling of secondary alcohol **31** with glutamine derivative **32** afforded the corresponding ester in 96% yield (Scheme 4). We employed a convergent strategy to bring fragments **21** and **33** through a HATU-mediated amide coupling, which successfully afforded the late-stage intermediate **34**. Following deallylation, we found that treatment with HCl to effect Boc deprotection also resulted in cleavage of the PMB group giving amino alcohol **35**. We were concerned over whether cyclization would occur through nitrogen or oxygen, but in the event upon treatment with HATU, clean cyclization occurred exclusively through nitrogen, giving macrolactam **36** in 81% yield over 2 steps. Due to the sensitivity of the APD motif, we first oxidized **36** to reveal the primary amide; however, the product was found to be challenging to purify due to its low solubility. The crude sample was concentrated and then directly subjected to a *N,N*′-disuccinimidyl carbonate (DSC)-mediated elimination affording (−)-rakicidin F (**3**) in 18% over 2 steps. To circumvent the insolubility issue of the late-stage intermediate, an alternative route was employed, where alcohol **36** was converted to a sulfonate,^17^ oxidized to remove the DMB group, and then directly treated with DBU to afford (−)-rakicidin F (**3**) in 32% over 3 steps, completing the total synthesis in 24 steps for the longest linear sequence with an overall yield of 3.0%. This compares favorably with Wang and Chen’s synthesis which was completed in 20 steps and 0.68% overall yield.^18^ The analytical data (NMR, IR, HRMS, and [α]_D_) of synthetic compound **3** confirmed the identity of (−)-rakicidin F.^6^

**Scheme 4:**
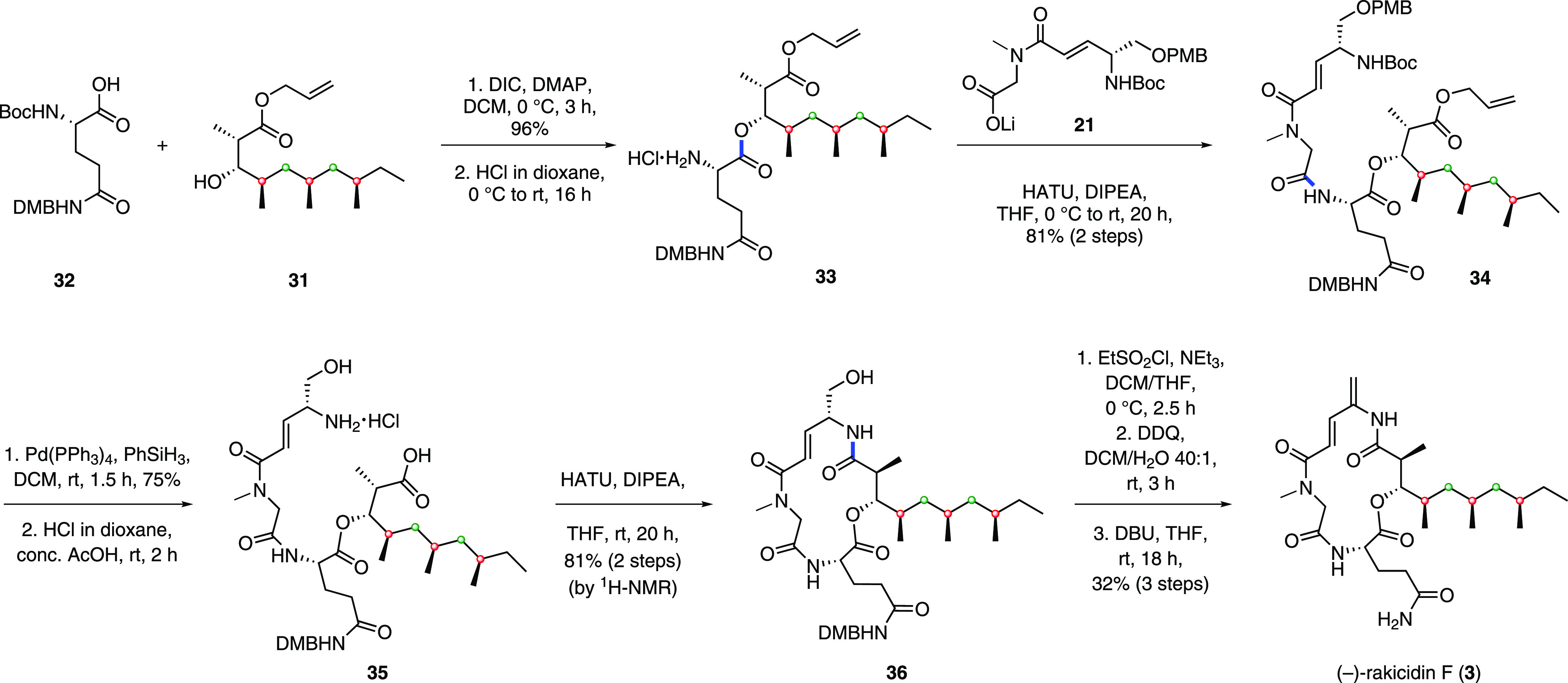
Fragment Coupling, Macrocyclization, and Completion of the Synthesis of (−)-Rakicidin F

In conclusion, we have developed a synthesis of (−)-rakicidin F using our lithiation–borylation methodology as the key step to construct the lipophilic side chain with high efficiency. This versatile methodology enabled the C-21 stereoisomers of the lipophilic side chain to be prepared, and comparison of the NMR data with the natural product suggested that the most likely stereochemistry for the side chain was syn. Completion of the synthesis of rakicidin F confirmed the syn stereochemistry of the natural product as well as the absolute stereochemistry as determined by Wang and Chen.

## Data Availability

The data underlying this study are available in the published article and its Supporting Information.

## Supporting Information Available

Experimental procedures, complete characterization data, copies of ^1^H and ^13^C NMR spectra (PDF)
