# Supplementary material for: Application of Lithiation–Borylation to the Total Synthesis of (−)-Rakicidin F
Source: Org Lett. 2022 Dec 20;24(51):9398–402. doi: 10.1021/acs.orglett.2c03716 (PMC9806854; doi:10.1021/acs.orglett.2c03716)
Supplement: Supplementary file 1 — ol2c03716_si_001.pdf [file ol2c03716_si_001.pdf]

SUPPORTING INFORMATION

Application of Lithiation–Borylation to the Total Synthesis of (–)-Rakicidin F

Christian P. Bold,[§] Kay Yeung,[§] Felix Pape,[§] Daniel Kaiser, Varinder K. Aggarwal*

School of Chemistry, University of Bristol, Cantock's Close, Bristol BS8 1TS, United Kingdom

*e-mail: v.aggarwal@bristol.ac.uk

TABLE OF CONTENTS

1. MATERIALS AND GENERAL METHODS S3

1.1. Glassware, Solvents and Reagents S3

1.2. Chromatography and Instrumentation S3

1.3. Naming of Compounds S3

2. EXPERIMENTAL DATA S4

2.1. Synthesis of Carboxylate 21 .... S4

2.1.1. methyl *N*-(*tert*-butoxycarbonyl)-*O*-(4-methoxybenzyl)-*L*-serinate (18) S5

2.1.2. *tert*-butyl (*R*)-(1-hydroxy-3-((4-methoxybenzyl)oxy)propan-2-yl)carbamate (SI-1) S5

2.1.3. *tert*-butyl (*S*)-(1-((4-methoxybenzyl)oxy)-3-oxopropan-2-yl)carbamate (19) S6

2.1.4. methyl *N*-(2-bromoacetyl)-*N*-methylglycinate (SI-2)[2] S6

2.1.5. methyl *N*-(2-(diethoxyphosphoryl)acetyl)-*N*-methylglycinate (20)[2] S7

2.1.6. methyl (*R*,*E*)-*N*-(4-((*tert*-butoxycarbonyl)amino)-5-((4-methoxybenzyl)oxy)pent-2-enoyl)-*N*-methylglycinate (SI-3) S7

2.1.7. lithium (*R*,*E*)-*N*-(4-((*tert*-butoxycarbonyl)amino)-5-((4-methoxybenzyl)oxy)pent-2-enoyl)-*N*-methylglycinate (21) S8

2.2. Synthesis of Fragment 32 .... S9

2.2.1. benzyl *N*2-(*tert*-butoxycarbonyl)-*N*5-(2,4-dimethoxybenzyl)-*L*-glutaminate (SI-4) S9

2.2.2. *N*2-(*tert*-butoxycarbonyl)-*N*5-(2,4-dimethoxybenzyl)-*L*-glutamine (32) S10

2.3. Synthesis of Side Chain Fragment 31 .... S11

2.3.1. (S)-3-methoxy-2-methyl-3-oxopropyl 2,4,6-triisopropylbenzoate (22) S12

2.3.2. (*R*)-3-hydroxy-2-methylpropyl 2,4,6-triisopropylbenzoate (SI-5) S13

2.3.3. (*S*)-2-methyl-3-((triethylsilyl)oxy)propyl 2,4,6-triisopropylbenzoate (23) S13

2.3.4. ((2*R*,3*S*)-3-(dimethyl(phenyl)silyl)-2-methyl-3-(4,4,5,5-tetramethyl-1,3,2-dioxaborolan-2-yl) propoxy)triethylsilane (24) S14

2.3.5. (((2*R*,3*S*,4*S*)-3-(dimethyl(phenyl)silyl)-2-methyl-4-(4,4,5,5-tetramethyl-1,3,2-dioxaborolan-2-yl)pentyl)oxy)triethylsilane (27) S14

2.3.6. (((2*R*,3*S*,4*R*)-3-(dimethyl(phenyl)silyl)-2,4-dimethyl-5-(4,4,5,5-tetramethyl-1,3,2-dioxaborolan-2-yl)pentyl)oxy)triethylsilane (SI-6) S15

2.3.7. (((2*R*,3*S*,4*R*,6*S*)-3-(dimethyl(phenyl)silyl)-2,4-dimethyl-6-(4,4,5,5-tetramethyl-1,3,2-dioxaborolan-2-yl)heptyl)oxy)triethylsilane (SI-7) S15

2.3.8. (((2*R*,3*S*,4*R*,6*R*)-3-(dimethyl(phenyl)silyl)-2,4,6-trimethyl-7-(4,4,5,5-tetramethyl-1,3,2-dioxaborolan-2-yl)heptyl)oxy)triethylsilane (SI-8) S16

2.3.9. (((2*R*,3*S*,4*R*,6*R*,8*S*)-3-(dimethyl(phenyl)silyl)-2,4,6-trimethyl-8-(4,4,5,5-tetramethyl-1,3,2-dioxaborolan-2-yl)nonyl)oxy)triethylsilane (28) S17

2.3.10. (((2*R*,3*S*,4*R*,6*R*,8*S*)-3-(dimethyl(phenyl)silyl)-2,4,6,8-tetramethyldec-9-en-1-yl)oxy)triethylsilane (29) S17

2.3.11. (((2*R*,3*S*,4*R*,6*R*,8*R*)-3-(dimethyl(phenyl)silyl)-2,4,6,8-tetramethyldecyl)oxy)triethylsilane (SI-9) S18

2.3.12. (2*R*,3*S*,4*R*,6*R*,8*R*)-2,4,6,8-tetramethyldecane-1,3-diol (30) S18

2.3.13. (2*S*,3*S*,4*R*,6*R*,8*R*)-3-hydroxy-2,4,6,8-tetramethyldecanal (SI-10) S19

2.3.14. (2*S*,3*S*,4*R*,6*R*,8*R*)-3-hydroxy-2,4,6,8-tetramethyldecanoic acid (SI-11) S19

2.3.15. allyl (2*S*,3*S*,4*R*,6*R*,8*R*)-3-hydroxy-2,4,6,8-tetramethyldecanoate (31) S20

2.4. Building Block Assembly S21

2.4.1. allyl (2*S*,3*S*,4*R*,6*R*,8*R*)-3-((*N*2-(*tert*-butoxycarbonyl)-*N*5-(2,4-dimethoxybenzyl)-*L*-glutaminyl)oxy)-2,4,6,8-tetramethyldecanoate (SI-12) S22

2.4.2. allyl (2*S*,3*S*,4*R*,6*R*,8*R*)-3-((*N*5-(2,4-dimethoxybenzyl)-*L*-glutaminyl)oxy)-2,4,6,8-tetramethyldecanoate (33) .... S**Error! Bookmark not defined.**

2.4.3. (2*S*,3*S*,4*R*,6*R*,8*R*)-1-(allyloxy)-2,4,6,8-tetramethyl-1-oxodecan-3-yl (6*R*,14*S*,*E*)-14-(3-((2,4-dimethoxybenzyl)amino)-3-oxopropyl)-6-(((4-methoxybenzyl)oxy)methyl)-2,2,10-trimethyl-4,9,12-trioxo-3-oxa-5,10,13-triazapentadec-7-en-15-oate (34) S23

2.4.4. (6*R*,14*S*,17*S*,18*S*,*E*)-14-(3-((2,4-dimethoxybenzyl)amino)-3-oxopropyl)-17-((2*R*,4*R*,6*R*)-4,6-dimethyloctan-2-yl)-6-(((4-methoxybenzyl)oxy)methyl)-2,2,10,18-tetramethyl-4,9,12,15-tetraoxo-3,16-dioxa-5,10,13-triazanonadec-7-en-19-oic acid (SI-13) S24

2.4.5. (2*S*,3*S*,4*R*,6*R*,8*R*)-3-((*N*2-(*N*-((*R*,*E*)-4-amino-5-hydroxypent-2-enoyl)-*N*-methylglycyl)-*N*5-(2,4-dimethoxybenzyl)-*L*-glutaminyl)oxy)-2,4,6,8-tetramethyldecanoic acid (35) S25

2.4.6. *N*-(2,4-dimethoxybenzyl)-3-((3*S*,11*R*,14*S*,15*S*,*E*)-15-((2*R*,4*R*,6*R*)-4,6-dimethyloctan-2-yl)-11-(hydroxymethyl)-7,14-dimethyl-2,5,8,13-tetraoxo-1-oxa-4,7,12-triazacyclopentadec-9-en-3-yl)propanamide (36) S26

2.4.7. ((3*S*,11*R*,14*S*,15*S*,*E*)-3-(3-((2,4-dimethoxybenzyl)amino)-3-oxopropyl)-15-((2*R*,4*R*,6*R*)-4,6-dimethyloctan-2-yl)-7,14-dimethyl-2,5,8,13-tetraoxo-1-oxa-4,7,12-triazacyclopentadec-9-en-11-yl)methyl ethanesulfonate (SI-14) S27

2.4.8. ((3*S*,11*R*,14*S*,15*S*,*E*)-3-(3-amino-3-oxopropyl)-15-((2*R*,4*R*,6*R*)-4,6-dimethyloctan-2-yl)-7,14-dimethyl-2,5,8,13-tetraoxo-1-oxa-4,7,12-triazacyclopentadec-9-en-11-yl)methyl ethanesulfonate (SI-15) S27

2.4.9. 3-((3*S*,14*S*,15*S*,*E*)-15-((2*R*,4*R*,6*R*)-4,6-dimethyloctan-2-yl)-7,14-dimethyl-11-methylene-2,5,8,13-tetraoxo-1-oxa-4,7,12-triazacyclopentadec-9-en-3-yl)propanamide (3) S28

2.5. Alternative Route S29

2.5.1. 3-((3*S*,11*R*,14*S*,15*S*,*E*)-15-((2*R*,4*R*,6*R*)-4,6-dimethyloctan-2-yl)-11-(hydroxymethyl)-7,14-dimethyl-2,5,8,13-tetraoxo-1-oxa-4,7,12-triazacyclopentadec-9-en-3-yl)propanamide (SI-16) S29

2.5.2. 3-((3*S*,14*S*,15*S*,*E*)-15-((2*R*,4*R*,6*R*)-4,6-dimethyloctan-2-yl)-7,14-dimethyl-11-methylene-2,5,8,13-tetraoxo-1-oxa-4,7,12-triazacyclopentadec-9-en-3-yl)propanamide (3) S30

2.6. Data Comparison of Synthesized (–)-Rakicidin F and Natural (+)-Rakicidin F[4] S31

2.6.1. ^{1}H NMR Data Comparison (CD_3OD) S31

2.6.2. ^{13}C NMR Data Comparison (CD_3OD) S32

2.6.3. HRMS, IR, $[\alpha]_D$ Data Comparison S33

2.7. Screening of Conditions S34

2.7.1. Reduction Methyl ester 18 .... S34

2.7.2. Horner-Wadsworth-Emmons Reaction S35

2.7.3. Allyl Group Cleavage S36

2.8. Comparison of the ^{13}C NMR Data of Rakicidin F with syn- and anti-configured Lipophilic Side Chains 30 and epi-30 .... S37

3. SPECTROSCOPIC DATA S38

4. REFERENCES S61

1. MATERIALS AND GENERAL METHODS

1.1. Glassware, Solvents and Reagents

All manipulations were performed with oven-dried (130 °C for a minimum of 12 h) or flame-dried glassware using standard Schlenk techniques under an atmosphere of nitrogen, unless otherwise stated.

All anhydrous solvents were commercially supplied or dried using an Anhydrous Engineering alumina column drying system (THF, toluene, Et_2O, CH_2Cl_2). Reagents were purchased from commercial sources and used as received. All organolithium reagents were titrated against *N*-benzylbenzamide.[1]

1.2. Chromatography and Instrumentation

Thin layer chromatography (TLC) was performed using Merck Kieselgel 60 F254 fluorescent treated silica adsorbed onto aluminium, components were visualized under UV light, or by staining with aqueous basic potassium permanganate followed by heating, *p*-anisaldehyde solution followed by heating, Hanessian's stain (CAM stain) followed by heating, or an ethanolic solution of phosphomolybdic acid followed by heating, as stated.

Flash column chromatography (FCC) was carried out using Sigma-Aldrich silica gel (60 Å, 230-400 mesh, 40-63 µm) or a Biotage Isolera^{TM} flash purification system. In cases where automated column chromatography was employed, the solvent gradient and flow rate are indicated.

NMR spectra were recorded at various field strengths, as indicated, using Bruker 400 MHz, Varian VNMR 400 MHz, Varian VNMR 500 MHz, or Bruker Cryo 500 MHz for ^{1}H, and ^{13}C acquisitions. All NMR spectra were recorded at 25 °C unless otherwise stated. Chemical shifts (δ) are reported in parts per million (ppm) and referenced $CDCl_3$ (^{1}H: 7.26 ppm; ^{13}C: 77.0 ppm) or DMSO-d_6 (^{1}H: 2.50 ppm; ^{13}C: 39.5 ppm) or CD_3OD (^{1}H: 3.31 ppm; ^{13}C: 49.0 ppm). Coupling constants (*J*) are given in Hertz (Hz) and refer to apparent multiplicities (s = singlet, d = doublet, t = triplet, q = quartet, quin = quintet, sex = hextet, hept = heptet, m = multiplet, br = broad signal, dd = doublet of doublets, dtt = doublet of triplets of triplets, dqd = doublet of quartets of doublets, br s = broad singlet, etc.). The ^{1}H NMR spectra are reported as follows: chemical shift (multiplicity, coupling constants, number of protons).

High resolution mass spectra (HRMS) were recorded on a Bruker Daltonics MicrOTOF II by Electrospray Ionisation (ESI); a Thermo Scientific QExactive by Electron Ionisation (EI); a Thermo Scientific Orbitrap Elite by ESI or Atmospheric Pressure Chemical Ionisation (APCI); or a Bruker UltrafleXtreme by Matrix-assisted Laser Desorption/Ionisation (MALDI).

IR spectra were recorded neat as a thin film on a Perkin Elmer Spectrum One FT-IR. Selected absorption maxima (ν_{max}) are reported in wavenumbers (cm^{-1}).

Optical rotations ($[\alpha]_D^T$) were recorded using a Bellingham & Stanley ADP 220 Polarimeter.

1.3. Naming of Compounds

Compound names are generated by ChemDraw Professional 20.0 software (PerkinElmer), following the IUPAC nomenclature.

2. EXPERIMENTAL DATA

2.1. Synthesis of Carboxylate 21

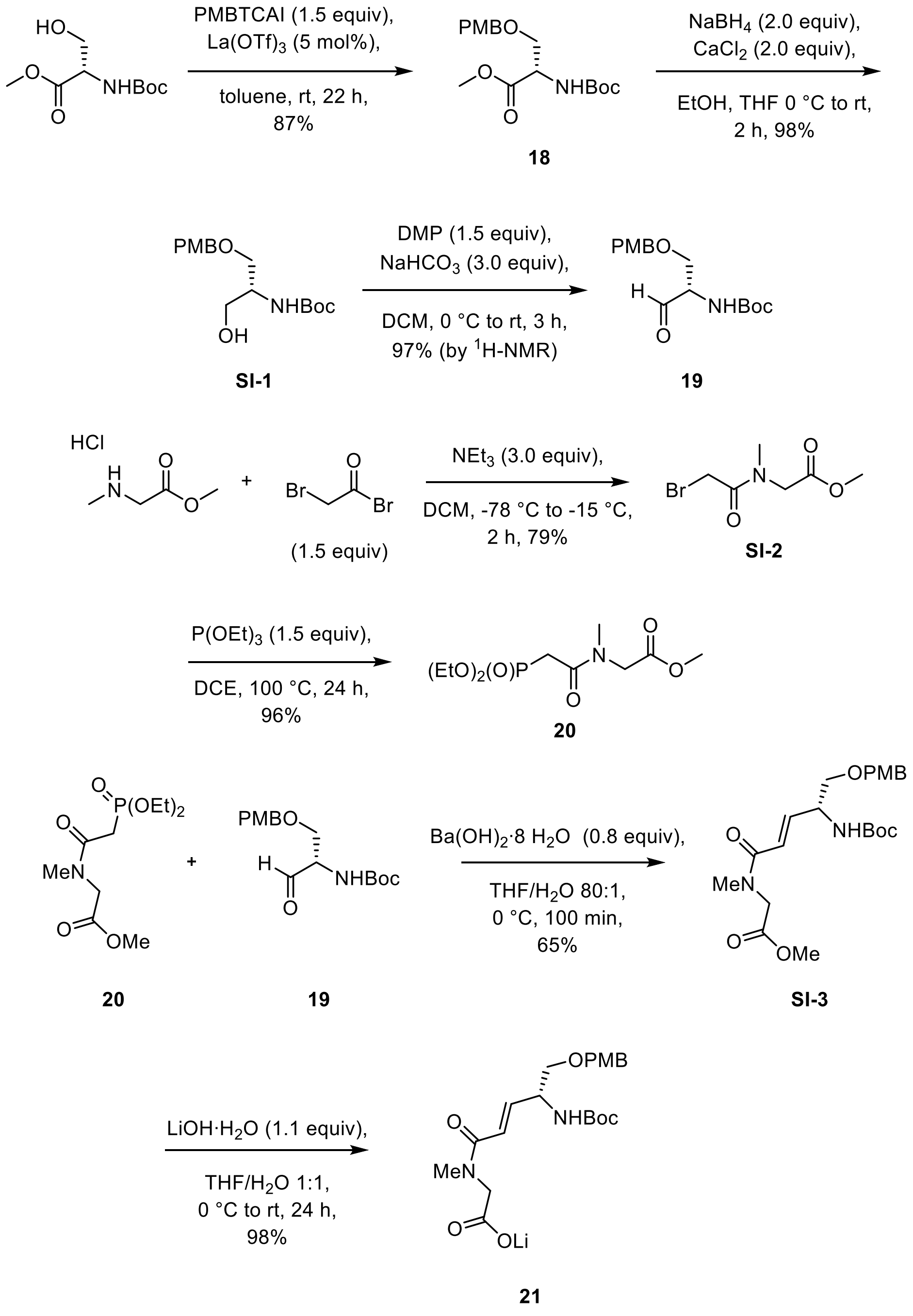

2.1.1. methyl *N*-(*tert*-butoxycarbonyl)-*O*-(4-methoxybenzyl)-*L*-serinate (18)

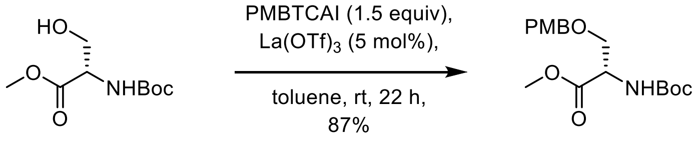

Boc-*L*-Ser-OMe **18**

In a 500 mL round bottom flask, Boc-*L*-Ser-OMe (4.00 g, 18.2 mmol, 1.0 equiv) and PMB-2,2,2-trichloroacetimidate (7.73 g, 27.4 mmol, 1.5 equiv) were dissolved in dry toluene (320 mL). $La(OTf)_3$ (535 mg, 0.912 mmol, 5 mol%) was added and the resulting colorless solution was stirred at rt for 22 h. Then, the reaction mixture was concentrated under reduced pressure to give 14.2 g of a white oily solid. The crude residue was purified by flash chromatography (SiO_2, Hex:EtOAc 4:1 to 2:1) to afford **18** (11.6 g, 48% pure by ^{1}H-NMR analysis with ISTD, impurity: trichloroacetamide) as a white oily solid. The residue was twice suspended in hexane (100 mL) and filtered. The two mother liquors were combined and concentrated under reduced pressure to obtain pure **18** (5.41 g, 15.9 mmol, 87%) as a pale-yellow oil.

TLC (SiO_2; Pent:Et_2O 1:1, $KMnO_4$, CAM, UV): R_f = 0.45. **$[\alpha]_D^{24}$** = +4.4 (c = 2.99 in $CHCl_3$). **^{1}H NMR** (400 MHz, $CDCl_3$) δ 7.21 – 7.16 (m, 2H), 6.89 – 6.83 (m, 2H), 5.38 (d, *J* = 8.8 Hz, 1H), 4.49 – 4.34 (m, 3H), 3.85 – 3.77 (m, 1H), 3.79 (s, 3H), 3.72 (s, 3H), 3.64 (dd, *J* = 9.5, 3.5 Hz, 1H), 1.44 (s, 9H). **^{13}C NMR** (101 MHz, $CDCl_3$) δ 171.3, 159.4, 155.6, 129.7, 129.4, 113.9, 80.0, 73.0, 69.7, 55.4, 54.1, 52.5, 28.4. **IR** (thin film): υ = 3358, 2956, 1713, 1513, 1245, 1153 cm^{-1}. **HRMS** (ESI): calc. for $C_{17}H_{25}O_6NNa$ $[M+Na]^+$: 362.1574 m/z; found: 362.1584 m/z. (see NMR spectra)

2.1.2. *tert*-butyl (*R*)-(1-hydroxy-3-((4-methoxybenzyl)oxy)propan-2-yl)carbamate (SI-1)

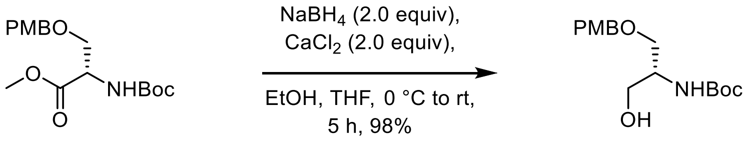

18 **SI-1**

In a 250 mL round bottom flask, anhydrous granular $CaCl_2$ (1.30 g, 11.7 mmol, 2.0 equiv) and $NaBH_4$ (443 mg, 11.7 mmol, 2.0 equiv) were suspended in dry THF (30 mL) at 0 °C under an atmosphere of nitrogen. To the resulting suspension, **18** (coevaporated with toluene (2x 50 mL), 1.99 g, 5.86 mmol, 1.0 equiv) dissolved in dry EtOH (30 mL) was added dropwise over 60 min. The resulting white suspension was stirred at 0 °C for additional 20 min and then allowed to warm to rt. After 5 h, the reaction mixture was cooled to 0 °C, then sat. aq. Rochelle's salt solution (150 mL) was added. The resulting white suspension was stirred at 0 °C for 20 min. EtOAc (200 mL) and water (100 mL) were added, and the layers were separated. The aq. layer was extracted with EtOAc (2x 200 mL). The org. layers were washed with brine (1x 200 mL), combined, dried over $MgSO_4$, filtrated, and concentrated under reduced pressure to give 2.44 g of a pale-yellow oil. The crude residue was purified by flash chromatography (SiO_2, pentane:Et_2O 1:1 to 1:2) to afford **SI-1** (1.79 g, 5.75 mmol, 98%) as a colorless oil.

TLC (SiO_2; Pent:Et_2O 1:3, $KMnO_4$, CAM, UV): R_f = 0.25. **$[\alpha]_D^{24}$** = +10.2 (c = 3.13 in $CHCl_3$). **^{1}H NMR** (500 MHz, $CDCl_3$) δ 7.28 – 7.23 (m, 2H), 6.93 – 6.88 (m, 2H), 5.19 (s, 1H), 4.52 – 4.42 (m, 2H), 3.86 – 3.77 (m, 5H), 3.73

– 3.66 (m, 1H), 3.66 – 3.57 (m, 2H), 2.73 (s, 1H), 1.46 (s, 9H). **^{13}C NMR** (126 MHz, $CDCl_3$) δ 159.5, 156.2, 129.8, 129.5, 114.0, 79.8, 73.3, 70.6, 64.3, 55.4, 51.7, 28.5. **IR** (thin film): υ = 3416, 2933, 1687, 1512, 1244, 1168, 1030 cm^{-1}. **HRMS** (ESI): calc. for $C_{16}H_{25}O_5NNa$ $[M+Na]^+$: 334.1625 m/z; found: 334.1628 m/z. (see NMR spectra)

2.1.3. *tert*-butyl (*S*)-(1-((4-methoxybenzyl)oxy)-3-oxopropan-2-yl)carbamate (19)

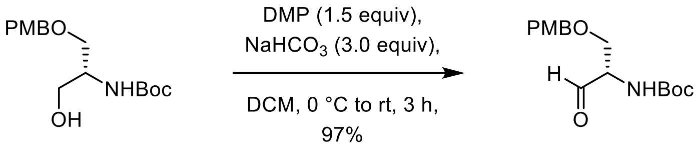

SI-1 **19**

In a 500 mL round bottom flask, **SI-1** (1.58 g, 5.07 mmol, 1.0 equiv) was dissolved in DCM (50 mL). $NaHCO_3$ (1.28 g, 15.2 mmol, 3.0 equiv) was added and the colorless solution with white $NaHCO_3$-particles was cooled to 0 °C. Then DMP (3.22 g, 7.60 mmol, 1.5 equiv) was added in one portion. The resulting colorless suspension was stirred at 0 °C for 30 min and then allowed to warm to rt. After 3 h, Dess-Martin workup solution[1] (200 mL) was added to the colorless suspension, which was then stirred for 75 min. DCM (200 mL) and additional Dess-Martin workup solution (100 mL) were added and the layers were separated. The aq. layer was extracted with DCM (2x 200 mL). The org. layers were washed with Dess-Martin workup solution (200 mL), followed by brine (200 mL), combined, dried over $MgSO_4$, filtrated, and concentrated under reduced pressure to afford crude **19** (96% purity by ^{1}H-NMR, 1.53 g, 4.94 mmol, 97%) as a yellow oil. The crude residue was directly used for step 2.1.6.

2.1.4. methyl *N*-(2-bromoacetyl)-*N*-methylglycinate (SI-2)[2]

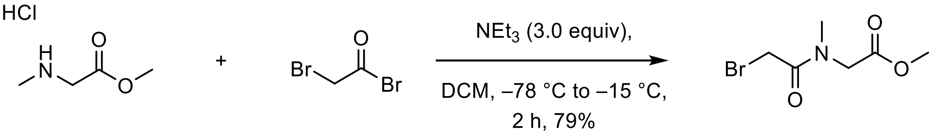

sarcosine methyl ester hydrochloride

bromoacetyl bromide (1.5 equiv)

SI-2

In a 500 mL round bottom flask, sarcosine methyl ester hydrochloride (1.79 g, 12.8 mmol, 1.0 equiv) was dissolved in dry DCM (140 mL). Triethylamine (5.36 mL, 38.5 mmol, 3.0 equiv) was added and the colorless solution was cooled to -78 ˚C using a cryostat. A solution of bromoacetyl bromide (2.32 mL, 19.2 mmol, 1.5 equiv) in DCM (20 mL) was added dropwise over 5 minutes using a syringe. The resulting weakly brownish solution was stirred at -78 °C for 30 min, at -60 °C for 15 min, at -35 °C for 15 min, at -12 °C for 60 min. After a total of 2 h, aq. 1 M HCl solution (230 mL) was added to the reaction mixture (weakly brown solution) and the layers were separated. The aq. layer was extracted with DCM (3x 250 mL). The org. layers were washed with brine (250 mL), combined, dried over $MgSO_4$, filtered, and concentrated under reduced pressure to obtain 3.61 g of a brown oil. The crude residue was purified by flash chromatography (SiO_2, pentane:Et_2O 1:3 to 1:4) to

[1] Dess-Martin work-up solution: $NaHCO_3$ (80 g) and $Na_2S_2O_3 \cdot 5\ H_2O$ (14 g) dissolved in 1000 ml water.

afford **SI-2** (2.27 g, 10.1 mmol, 79%) as a yellow oil.

TLC (SiO_2; Pent:Et_2O 1:3, $KMnO_4$, UV): R_f = 0.27. **^{1}H NMR** (400 MHz, $CDCl_3$) δ 4.13 (s, 2H), 3.91 (s, 2H), 3.74 (s, 3H), 3.17 (s, 3H) (in agreement with lit. [2]). (see NMR spectra)

2.1.5. methyl *N*-(2-(diethoxyphosphoryl)acetyl)-*N*-methylglycinate (20)[2]

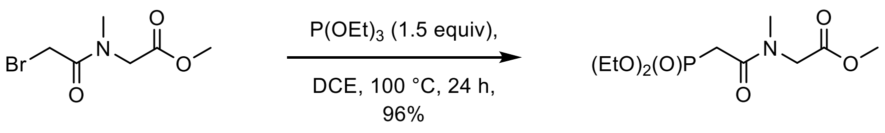

SI-2 **20**

In a 100 mL round bottom flask, **SI-2** (4.44 g, 19.8 mmol, 1.0 equiv) was dissolved in anhydrous 1,2-dichloroethane (20 mL). Triethylphosphite (5.10 mL, 29.7 mmol, 1.5 equiv) was added in one portion and the reaction was heated with an oil bath to reflux (100 °C). After 24 h, the reaction mixture was allowed to cool to ambient temperature and concentrated under reduced pressure. The crude residue was purified by flash chromatography (SiO_2, EtOAc:MeOH 98:2 to 9:1) to afford **20** (5.34 g, 19.0 mmol, 96%) as a pale-reddish oil.

TLC (SiO_2; EtOAc:MeOH 9:1, $KMnO_4$, UV): R_f = 0.34. Mixture of rotamers, NMR data is reported for the major rotamer. **^{1}H NMR** (400 MHz, $CDCl_3$) δ 4.20 – 4.08 (m, 6H), 3.70 (s, 3H), 3.17 (s, 3H), 3.08 (d, J = 22.1 Hz, 2H), 1.34 – 1.28 (m, 6H) **^{13}C NMR** (101 MHz, $CDCl_3$) δ 169.4, 165.8 (d, J = 5.4 Hz), 62.8 (d, J = 6.6 Hz), 52.2, 49.7, 37.9, 33.4 (d, J = 133.2 Hz), 16.4 (d, J = 6.2 Hz) (major rotamer – in agreement with lit. [2]). (see NMR spectra)

2.1.6. methyl (*R*,*E*)-*N*-(4-((*tert*-butoxycarbonyl)amino)-5-((4-methoxybenzyl)oxy)pent-2-enoyl)-*N*-methylglycinate (SI-3)

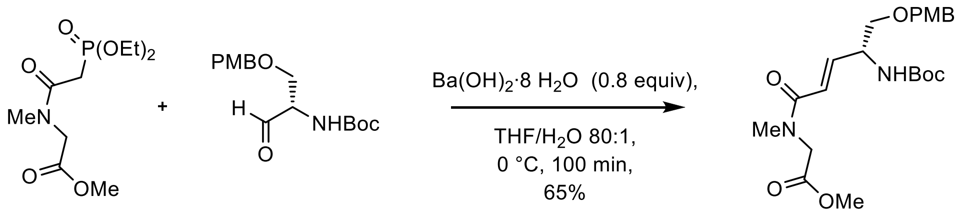

20 **19** **SI-3**

In a 250 mL round bottom flask, **20** (500 mg, 1.78 mmol, 1.0 equiv) was dissolved in dry THF (40 mL) and cooled to 0 °C. $Ba(OH)_2$·8 H_2O (449 mg, 1.42 mmol, 0.80 equiv) was added, and the resulting colorless solution with white particles was stirred at 0 °C for 5 min. Then, a solution of **19** (660 mg, 2.13 mmol, 1.2 equiv) dissolved in wet THF (41 mL, THF:H_2O 40:1) was added dropwise. After 100 min, sat. aq. $NaHCO_3$ (200 mL) and EtOAc (200 mL) were added to the white suspension and the layers were separated. The aq. layer was extracted with EtOAc (2x 200 mL). The org. layers were washed with brine (200 mL), combined, dried over $MgSO_4$, filtered, and concentrated under reduced pressure to give 1.15 g of a pale-yellow oil (^{1}H-NMR of crude mixture: E/Z = 5.7:1). The crude residue was purified by flash chromatography (SiO_2, pentane:EtOAc 2:1 to 100% EtOAc) to afford **SI-3** (503 mg, 1.15 mmol, 65%, E/Z > 95:5) as a colorless oil.

TLC (SiO_2; Pent:EtOAc 1:4, $KMnO_4$, CAM, UV): R_f = 0.37. **$[\alpha]_D^{22}$** = +2.4 (c = 3.29 in MeOH). Mixture of rotamers, NMR data is reported for the major rotamer. **^{1}H NMR** (400 MHz, CD_3OD) δ 7.25 (dd, J = 8.6, 3.3 Hz, 2H), 6.91

– 6.84 (m, 2H), 6.76 (dd, *J* = 15.3, 5.4 Hz, 1H), 6.56 (dd, *J* = 15.3, 1.6 Hz, 1H), 4.51 – 4.38 (m, 3H), 4.17 (s, 2H), 3.77 (s, 3H), 3.72 (s, 3H), 3.51 (d, *J* = 5.9 Hz, 2H), 3.15 (s, 3H), 1.45 (s, 9H). **^{13}C NMR** (101 MHz, CD_3OD) δ 171.2, 169.0, 160.8, 157.7, 145.3, 131.3, 130.6, 121.7, 114.8, 80.5, 73.8, 72.2, 55.7, 53.1, 52.6, 50.7, 37.3, 28.7. **IR** (thin film): υ = 3325, 2975, 2866, 1749, 1708, 1664, 1613, 1513, 1365, 1246, 1171, 1104, 1031, 820 cm^{-1}. **HRMS** (ESI): calc. for $C_{22}H_{32}O_7N_2Na$ [M+Na]$^+$: 459.2102 m/z; found: 459.2112 m/z. (see NMR spectra)

2.1.7. lithium (*R*,*E*)-*N*-(4-((*tert*-butoxycarbonyl)amino)-5-((4-methoxybenzyl)oxy)pent-2-enoyl)-*N*-methylglycinate (21)

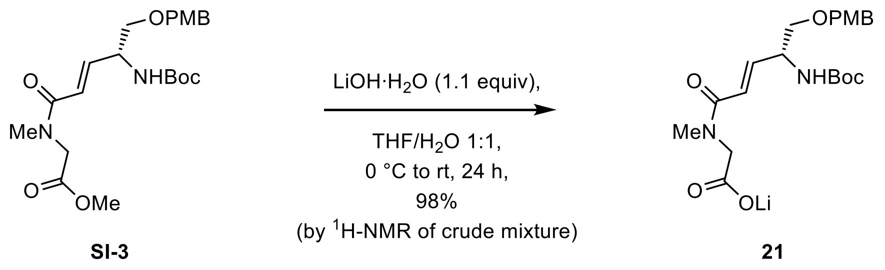

In a 25 mL round bottom flask, **SI-3** (433 mg, 0.99 mmol, 1.0 equiv) was dissolved in THF/H_2O (1:1, 10 mL) and cooled to 0 ˚C. LiOH·H_2O (45.8 mg, 1.09 mmol, 1.10 equiv) was added in one portion. The resulting colorless solution was stirred at 0 ˚C for 5 h and then allowed to warm to rt. After stirring additional 24 h, the reaction mixture was concentrated under reduced pressure at 40 °C. The residue (colorless oil) was dissolved twice in THF (10 mL) and concentrated again to remove residual water, giving crude **21** (454 mg, 91% purity, 0.97 mmol, 98%) as an off-white foam. The crude residue was directly used for step 2.4.3.

2.2. Synthesis of Fragment 32

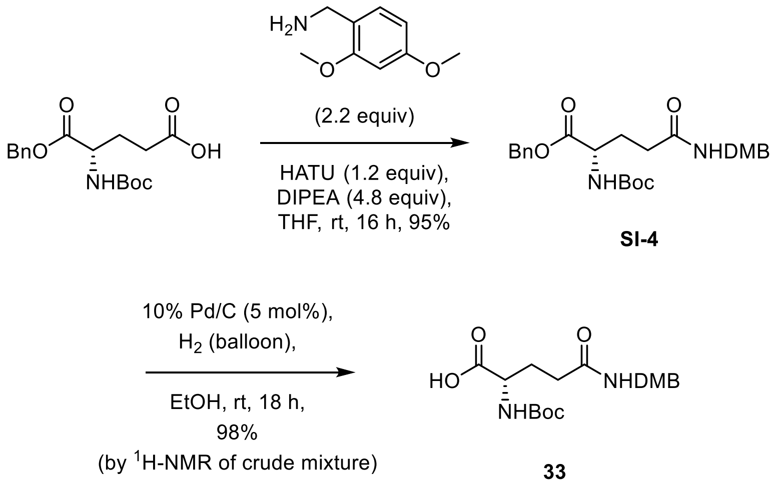

SI-4

33

2.2.1. benzyl *N*^{2}-(*tert*-butoxycarbonyl)-*N*^{5}-(2,4-dimethoxybenzyl)-*L*-glutaminate (SI-4)

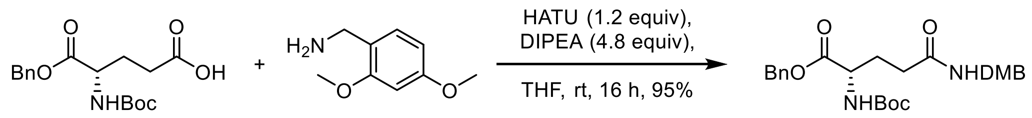

1-Bn-*N*-Boc-*L*-glutamate

2,4-dimethoxy-benzylamine

SI-4

In a 250 mL round bottom flask, 1-benzyl *N*-(*tert*-butoxycarbonyl)-*L*-glutamate (4.00 g, 11.9 mmol, 1.0 equiv) was dissolved in dry THF (60 mL). DIPEA (9.91 mL, 56.9 mmol, 4.8 equiv) and 2,4-dimethoxybenzylamine (3.92 mL, 26.1 mmol, 2.2 equiv), followed by HATU (5.41 g, 14.2 mmol, 1.2 equiv) was added. The resulting strong yellow suspension was stirred at rt for 16 h. Then, sat. aq. $NaHCO_3$ (200 mL) and EtOAc (200 mL) were added, and the layers were separated. The aq. layer was extracted with EtOAc (2x 200 mL). The org. layers were washed with $NaHCO_3$ (200 mL) and with brine (200 mL), combined, dried over $MgSO_4$, filtered, and concentrated under reduced pressure to give 13.1 g of a yellow oil. The crude residue was purified by flash chromatography (SiO_2, Et_2O) to afford **SI-4** (5.50 g, 11.3 mmol, 95%) as a white solid.

Remark: The product fractions contain tetramethylurea as an inseparable impurity (**SI-4**:tetramethylurea = 1:0.2).

TLC (SiO_2; 100% Et_2O, CAM, UV): R_f = 0.36. **$[\alpha]_D^{22}$** = -1.0 (c = 4.79 in $CHCl_3$). **^{1}H NMR** (400 MHz, $CDCl_3$) δ 7.36 – 7.27 (m, 5H), 7.15 (d, *J* = 8.2 Hz, 1H), 6.44 (d, *J* = 2.3 Hz, 1H), 6.41 (dd, *J* = 8.2, 2.4 Hz, 1H), 6.11 (br s, 1H), 5.35 (d, *J* = 8.2 Hz, 1H), 5.20 – 5.07 (m, 2H), 4.35 – 4.23 (m, 1H), 4.33 (d, *J* = 5.7 Hz, 2H), 3.80 (s, 3H), 3.78 (s, 3H), 2.27 – 2.09 (m, 3H), 2.02 – 1.90 (m, 1H), 1.41 (s, 9H). **^{13}C NMR** (101 MHz, $CDCl_3$) δ 172.3, 171.4, 160.6, 158.7, 155.8, 135.5, 130.6, 128.7, 128.5, 128.4, 118.9, 104.1, 98.7, 80.0, 67.2, 55.5, 55.4, 53.4, 39.1, 32.7, 28.7, 28.4. **IR** (thin film): υ = 3309, 2976, 2933, 1709, 1507, 1156, 1031 cm^{-1}. **HRMS** (ESI): calc. for $C_{26}H_{35}O_7N_2$ $[M+H]^+$: 487.2439 m/z; found: 487.2441 m/z. (see NMR spectra)

2.2.2. *N*[2]-(*tert*-butoxycarbonyl)-*N*[5]-(2,4-dimethoxybenzyl)-*L*-glutamine (32)

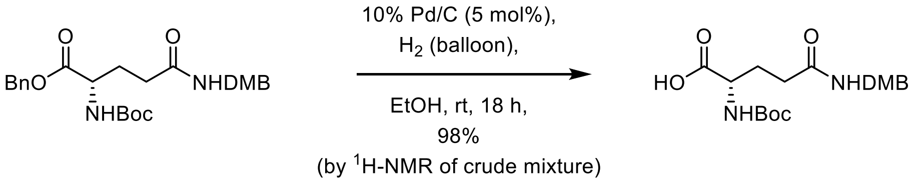

SI-4 **32**

In a 250 mL round bottom flask, **SI-4** (5.31 g, 10.9 mmol, 1.0 equiv) was dissolved in dry EtOH (100 mL). After the addition of Pd/C 10% (581 mg, 0.55 mmol, 5 mol%), the reaction vessel was subjected to three cycles of evacuation followed by venting with N_2. Then, the reaction vessel was subjected to three cycles of evacuation followed by venting with H_2 (balloon). The black suspension was stirred under an H_2 atmosphere for 18 h. After this time, the reaction mixture was filtered through a Celite pad (2 cm) and the residue was washed with EtOAc (200 mL). The filtrate was concentrated under reduced pressure to give crude **32** (4.64, 91% purity by ^{1}H-NMR, 10.7 mmol, 98%) as a white foam and was used without further purification for step 2.4.1.

Remark: The crude mixture contains tetramethylurea as impurity (from the previous step) (**32**:tetramethylurea = 1:0.2)

TLC (SiO_2; EtOAc + 1 drop AcOH, $KMnO_4$, UV): R_f = 0.34. **$[\alpha]_D^{21}$** = +14.6 (c = 4.13 in $CHCl_3$). **^{1}H NMR** (400 MHz, $CDCl_3$) δ 11.23 (br s, 1H), 7.13 (d, J = 8.1 Hz, 1H), 6.69 (br s, 1H), 6.44 – 6.35 (m, 2H), 5.57 (d, J = 7.4 Hz, 1H), 4.33 (d, J = 5.5 Hz, 2H), 4.27 – 4.18 (m, 1H), 3.79 (s, 3H), 3.76 (s, 3H), 2.42 – 2.20 (m, 2H), 2.19 – 2.06 (m, 1H), 2.05 – 1.87 (m, 1H), 1.40 (s, 9H). **^{13}C NMR** (101 MHz, $CDCl_3$) δ 174.1, 173.1, 160.7, 158.6, 156.0, 130.6, 118.3, 104.1, 98.6, 80.0, 55.5, 55.4, 53.1, 39.3, 32.7, 29.3, 28.4. **IR** (thin film): υ = 3319, 2975, 2935, 1706, 1614, 1507, 1156, 1031 cm^{-1}. **HRMS** (ESI): calc. for $C_{19}H_{29}O_7N_2$ $[M+H]^+$: 397.1975 m/z; found: 397.1983 m/z. (see NMR spectra)

2.3. Synthesis of Side Chain Fragment 31

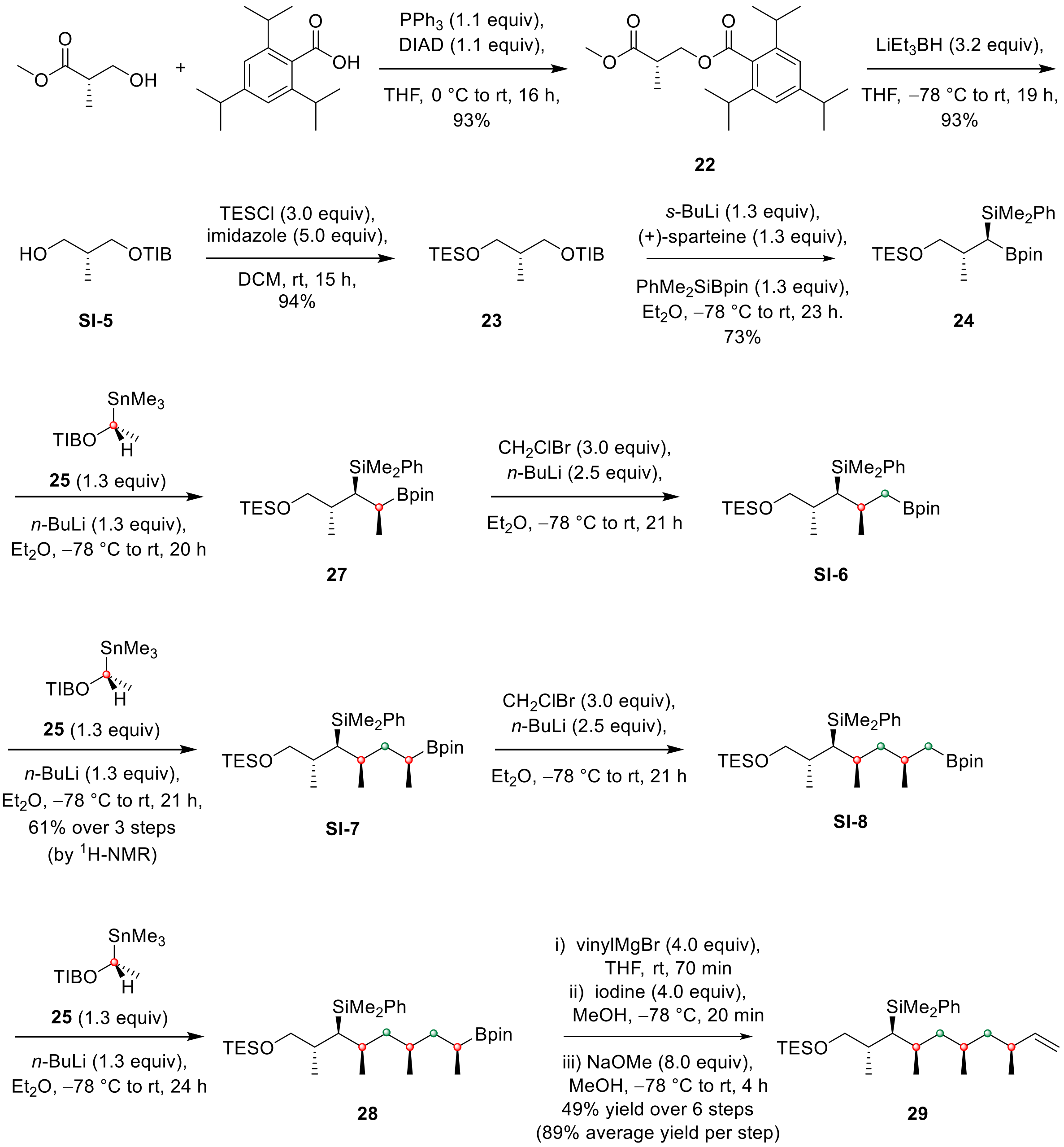

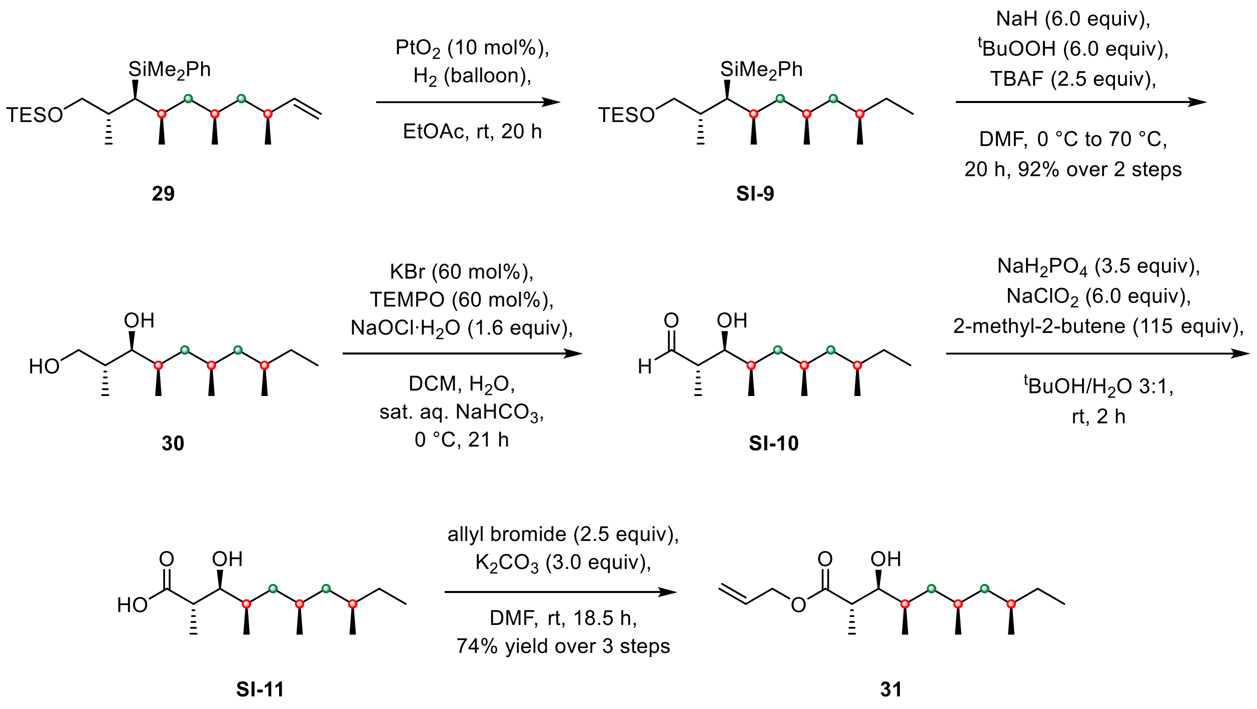

2.3.1. (S)-3-methoxy-2-methyl-3-oxopropyl 2,4,6-triisopropylbenzoate (22)

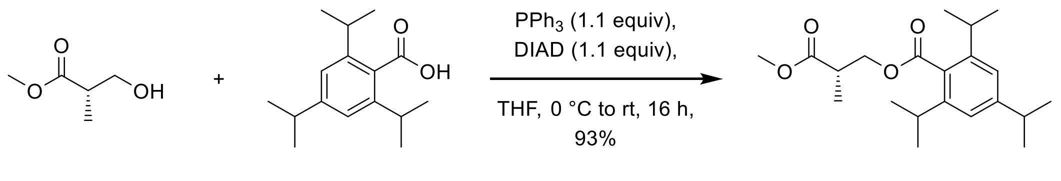

(*S*)-Roche ester 2,4,6-triisopropylbenzoic acid **22**

In a 250 mL round bottom flask, triphenylphosphine (4.44 g, 16.9 mmol, 1.1 equiv), 2,4,6-triisopropylbenzoic acid (3.82 g, 15.4 mmol, 1.0 equiv) and (*S*)-Roche ester (2.00 g, 16.9 mmol, 1.1 equiv) were dissolved in dry THF (31 mL) and cooled to 0 °C. Then diisopropyl azodicarboxylate (3.33 mL, 16.9 mmol, 1.1 equiv) was added dropwise (DIAD decolorized upon addition to the reaction mixture). The resulting pale-yellow solution was allowed to warm to rt. After a total of 16 h, the reaction mixture was concentrated under reduced pressure and the residue was purified by flash chromatography (SiO_2, pent:Et_2O 98:2 to 95:5) to afford **22** (5.00 g, 14.3 mmol, 93%) as a colorless oil.

TLC (SiO_2; pent:Et_2O 10:1, CAM, UV): R_f = 0.22. **$[\alpha]_D^{23}$** = +13.5 (c = 3.72 in $CHCl_3$). **^{1}H NMR** (500 MHz, $CDCl_3$) δ 7.01 (s, 2H), 4.49 (dd, *J* = 10.9, 7.4 Hz, 1H), 4.39 (dd, *J* = 10.9, 5.3 Hz, 1H), 3.69 (s, 3H), 2.95 – 2.86 (m, 2H), 2.82 (hept, *J* = 6.8 Hz, 2H), 1.27 (d, *J* = 7.2 Hz, 3H), 1.25 (d, *J* = 7.0 Hz, 6H), 1.24 (d, *J* = 7.0 Hz, 12H). **^{13}C NMR** (101 MHz, $CDCl_3$) δ 174.2, 170.8, 150.3, 145.0, 130.3, 121.0, 66.3, 52.0, 39.1, 34.5, 31.7, 24.3, 24.1, 14.2. **IR** (thin film): υ = 2961, 2871, 1728, 1606, 1461, 1384, 1363, 1249, 1201, 1136, 1068 cm^{-1}. **HRMS** (ESI): calc. for $C_{21}H_{32}O_4Na$ $[M+Na]^+$: 371.2193 m/z; found: 371.2190 m/z. (see NMR spectra)

2.3.2. (*R*)-3-hydroxy-2-methylpropyl 2,4,6-triisopropylbenzoate (SI-5)

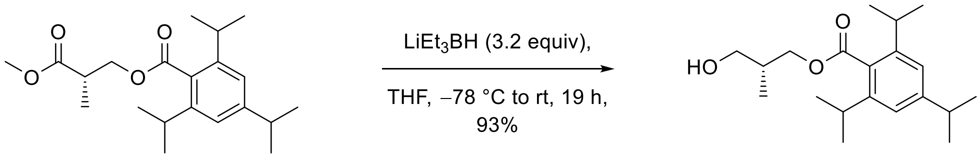

22 **SI-5**

In a 25 mL round bottom flask, **22** (732 mg, 2.10 mmol, 1.0 equiv) was dissolved in THF (10 mL) and cooled to –78 °C. Then Super Hydride® ($LiEt_3BH$, 1 M in THF, 6.72 mL, 6.72 mmol, 3.2 equiv) was added dropwise to the colorless solution. The reaction mixture was stirred at –78 °C for additional 90 min and then allowed to warm to rt. After a total 19 h, the reaction mixture was slowly added, at rt, to sat. aq. Rochelle's salt (50 mL) (effervescence observed). The mixture was stirred for 1.5 h at rt, then water (40 mL) and EtOAc (100 mL) were added, and the layers were separated. The aq. layer was extracted with EtOAc (2x 100 mL). The org. layers were washed with brine (100 mL), combined, dried over $MgSO_4$, filtered, and concentrated under reduced pressure to give 1.91 g of a colorless oil. The crude residue was purified by column chromatography (SiO_2, pent:Et_2O 2:1 to 1:1) to afford **SI-5** (628 mg, 1.96 mmol, 93%) as a colorless oil.

TLC (SiO_2; pent:Et_2O 1:1, CAM, $KMnO_4$, UV): R_f = 0.37. **$[\alpha]_D^{18}$** = +2.20 (c = 4.19 in $CHCl_3$). **^{1}H NMR** (400 MHz, $CDCl_3$) δ 7.03 (s, 2H), 4.31 (d, *J* = 5.7 Hz, 2H), 3.66 – 3.53 (m, 2H), 2.97 – 2.78 (m, 3H), 2.24 (br s, 1H), 2.16 – 2.02 (m, 1H), 1.26 (d, *J* = 6.9 Hz, 18H), 1.02 (d, *J* = 6.9 Hz, 3H). **^{13}C NMR** (101 MHz, $CDCl_3$) δ 171.5, 150.3, 144.9, 130.5, 121.0, 67.1, 64.8, 35.6, 34.5, 31.7, 24.27, 24.25, 24.0, 13.9. **IR** (thin film): υ = 3442, 2960, 2931, 2871, 1724, 1606, 1461, 1250, 1138, 1075, 1045, 876 cm^{-1}. **HRMS** (ESI): calc. for $C_{20}H_{32}O_3Na$ $[M+Na]^+$: 343.2249 m/z; found: 343.2244 m/z. (see NMR spectra)

2.3.3. (*S*)-2-methyl-3-((triethylsilyl)oxy)propyl 2,4,6-triisopropylbenzoate (23)

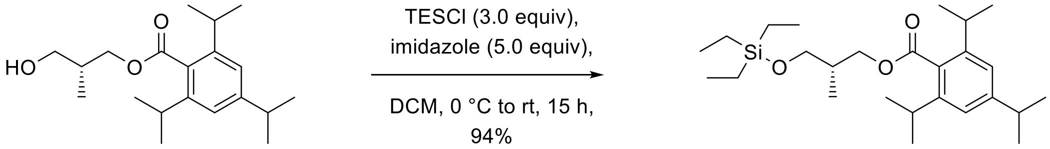

SI-5 **23**

In a flame-dried round bottom flask, **SI-5** (3.42 g, 10.7 mmol, 1.0 equiv) and imidazole (3.64 g, 53.4 mmol, 5.0 equiv) were dissolved in DCM (35.6 mL) and cooled to 0 °C. Then, TESCl (1.0 M in THF, 32.0 mL, 32.0 mmol, 3.0 equiv) was added dropwise. The reaction mixture was allowed to warm to rt and was stirred overnight. After 15 h, water (50 mL) was added, and the layers were separated. The aq. layer was extracted with DCM (3x 40 mL). The combined org. layers were washed with brine (30 mL), dried over $MgSO_4$, filtered, and concentrated under reduced pressure to give 8.01 g of a light-brown oil. The crude residue was purified through Biotage column chromatography (SiO_2, 100% pent to pent:Et_2O 9:1) to afford **23** (4.36 g, 10.0 mmol, 94%) as a colorless oil.

TLC (SiO_2; pent:Et_2O 20:1, $KMnO_4$, UV): R_f = 0.46. **$[\alpha]_D^{20}$** = -1.04 (c = 4.23 in MeOH). **^{1}H NMR** (400 MHz, $CDCl_3$) δ 7.02 (s, 2H), 4.32 (dd, *J* = 10.9, 5.8 Hz, 1H), 4.21 (dd, *J* = 10.9, 6.1 Hz, 1H), 3.61 – 3.52 (m, 2H), 2.96 – 2.79 (m, 3H), 2.14 – 1.99 (m, 1H), 1.28 – 1.23 (m, 18H), 1.00 (d, *J* = 6.8 Hz, 3H), 0.97 (t, *J* = 7.9 Hz, 9H), 0.60 (q, *J* = 7.8 Hz, 6H). **^{13}C NMR** (101 MHz, $CDCl_3$) δ 171.3, 150.1, 144.9, 130.9, 121.0, 67.2, 64.7, 35.7, 34.6, 31.7, 24.33, 24.31, 24.1, 14.1, 6.9, 4.5. **IR** (thin film): υ = 2959, 2875, 1726, 1607, 1460, 1250, 1074 cm^{-1}. **HRMS** (ESI): calc. for $C_{26}H_{47}O_3Si$ $[M+H]^+$: 435.3289 m/z; found: 435.3276 m/z. (see NMR spectra)

2.3.4. ((2*R*,3*S*)-3-(dimethyl(phenyl)silyl)-2-methyl-3-(4,4,5,5-tetramethyl-1,3,2-dioxaborolan-2-yl)propoxy)triethylsilane (24)

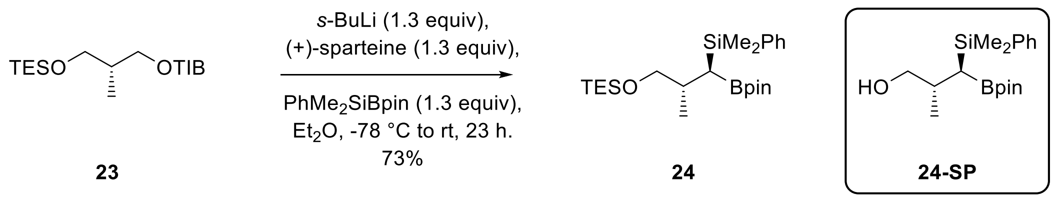

In a flame-dried Schlenk tube, **23** (3.00 g, 6.90 mmol, 1.0 equiv) and (+)-sparteine (2.06 mL, 2.10 g, 8.97 mmol, 1.3 equiv) were dissolved in dry Et_2O (29 mL) and cooled to -78 °C. *s*-BuLi (1.3 M in hexanes, 6.90 mL, 8.97 mmol, 1.3 equiv) was added *via* slow addition at -78 °C (0.2 mL/min) (colorless solution → orange solution → dark brown solution). The reaction mixture was stirred for 4 h at -78 °C. Then, $PhMe_2SiBpin$ (2.45 mL, 2.35 g, 8.97 mmol, 1.3 equiv) was added (neat) to the reaction mixture *via* slow addition (0.08 mL/min) (brown solution → yellow solution). The reaction mixture was stirred for 2 h at -78 °C and then allowed to warm to rt (removal of cooling bath). After additional 16 h, Et_2O (30 mL) and aq. 2 M HCl (30 mL) were added to the reaction mixture and the layers were separated. The aq. layer was extracted with Et_2O (2x 50 mL). The org. layers were washed with aq. 2 M HCl (2x 30 mL), brine (1x 30 mL), combined, dried over $MgSO_4$, filtered, and concentrated under reduced pressure to give 5.84 g of a colorless oil with white particles. The crude residue was purified by column chromatography (SiO_2, pent:Et_2O 100:1 to 50:1) to afford **24** (2.27 g, 5.05 mmol, 73%) as a colorless oil. In addition, the TES-deprotected side-product **24-SP** (299 mg, 0.89 mmol, 13%) was isolated.

TLC (SiO_2; pent:Et_2O 25:1, $KMnO_4$, UV): R_f = 0.23. **$[\alpha]_D^{20}$** = -3.99 (c = 4.21 in MeOH). **^{1}H NMR** (400 MHz, $CDCl_3$) δ 7.59 – 7.51 (m, 2H), 7.35 – 7.29 (m, 3H), 3.43 (dd, *J* = 9.6, 5.2 Hz, 1H), 3.18 (dd, *J* = 9.6, 8.7 Hz, 1H), 1.91 – 1.76 (m, 1H), 1.20 (s, 6H), 1.15 (s, 6H), 1.01 (d, *J* = 6.8 Hz, 3H), 0.90 (t, *J* = 7.9 Hz, 9H), 0.75 (d, *J* = 5.5 Hz, 1H), 0.55 – 0.45 (m, 6H), 0.38 (s, 3H), 0.34 (s, 3H). **^{13}C NMR** (101 MHz, $CDCl_3$) δ 139.8, 134.0, 128.8, 127.7, 82.8, 70.2, 34.5, 25.3, 25.0, 18.0, 16.7, 6.9, 4.6, -1.0, -1.7. **IR** (thin film): υ = 2955, 2876, 1458, 1370, 1306, 1247, 1144, 1084, 813, 728 cm^{-1}. **HRMS** (MALDI): calc. for $C_{24}H_{45}O_3BSi_2Na$ $[M+Na]^+$: 471.2898 m/z; found: 471.2890 m/z. (see NMR spectra)

2.3.5. (((2*R*,3*S*,4*S*)-3-(dimethyl(phenyl)silyl)-2-methyl-4-(4,4,5,5-tetramethyl-1,3,2-dioxaborolan-2-yl)pentyl)oxy)triethylsilane (27)

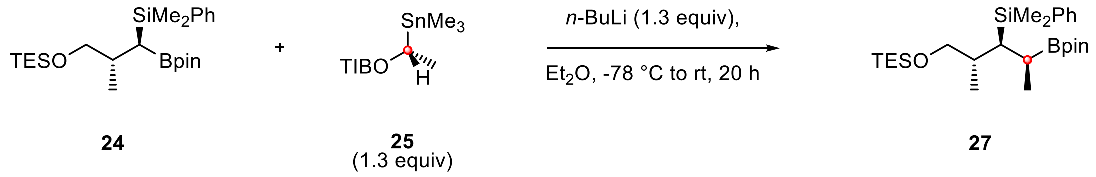

Titration of *n*-BuLi:[1] *N*-benzylbenzamide (105.6 mg, 0.500 mmol) was dissolved in dry THF (5.0 mL) and cooled to -40 °C. Then, *n*-BuLi (approx. 1.6 M in hexanes) was added dropwise until the color of the solution changed from colorless to dark blue. This procedure was repeated 3x. Average consumption: 310 µL → *n*-BuLi = 1.61 M.

In a flame-dried Schlenk flask, stannane **25**[3] (2.82 g, 6.42 mmol, 1.3 equiv) was dissolved in dry Et_2O (26 mL) and cooled to -78 °C. At this temperature *n*-BuLi (1.61 M in hexanes, 3.99 mL, 6.42 mmol, 1.3 equiv) was added dropwise over 40 min (0.1 mL/min) to the colorless solution. After completion of the addition, the resulting yellow solution was stirred at -78 °C for 80 min. Then, a solution of **24** (2.22 g, 4.94 mmol, 1.0 equiv) in dry Et_2O (10 mL) was added dropwise (0.1 mL/min). The resulting pale-yellow solution was stirred at -78 °C for 3.5 h and then allowed to warm to rt. After further 12.5 h (overnight), the reaction mixture was filtered through a pad of silica [~40 mm depth of wetted (Et_2O) silica, using a filter frit connected directly to a round bottom flask] to give a pale-yellow solution. The silica was washed with Et_2O (80 mL). The filtrate was concentrated under reduced pressure to give 3.18 g of a pale-yellow oil. The crude mixture was used directly for step 2.3.6.

2.3.6. (((2*R*,3*S*,4*R*)-3-(dimethyl(phenyl)silyl)-2,4-dimethyl-5-(4,4,5,5-tetramethyl-1,3,2-dioxaborolan-2-yl)pentyl)oxy)triethylsilane (SI-6)

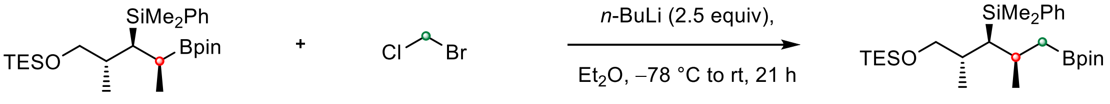

27 bromochloromethane (3.0 equiv) **SI-6**

In a flame-dried Schlenk flask, crude **27** (containing a maximum of 2.35 g, 4.94 mmol, 1.0 equiv) was dissolved in Et_2O (60 mL). Bromochloromethane (0.96 mL, 1.92 g, 14.8 mmol, 3.0 equiv) was added and the resulting colorless solution was cooled to -78 °C. Then *n*-BuLi (1.61 M in hexanes, 7.67 mL, 12.4 mmol, 2.5 equiv) was added slowly (1.7 mL/h). The resulting colorless solution was stirred for another 1.5 h at -78 °C and was then allowed to warm to rt. After further 15 h (overnight), the reaction mixture (pale-yellow suspension) was filtered through pad of silica [~40 mm depth of wetted (Et_2O) silica, using a filter frit connected directly to a round bottom flask] to give a pale-yellow solution. The silica was washed with Et_2O (140 mL). The filtrate was concentrated under reduced pressure to give 3.32 g of a pale-yellow oil. The crude mixture was used directly for step 2.3.7.

2.3.7. (((2*R*,3*S*,4*R*,6*S*)-3-(dimethyl(phenyl)silyl)-2,4-dimethyl-6-(4,4,5,5-tetramethyl-1,3,2-dioxaborolan-2-yl)heptyl)oxy)triethylsilane (SI-7)

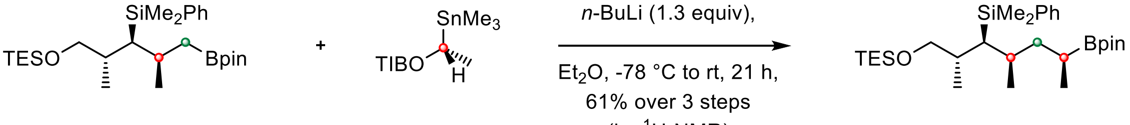

SI-6 **25** (1.3 equiv) **SI-7**

In a flame-dried Schlenk flask, stannane **25**[3] (2.82 g, 6.42 mmol, 1.3 equiv) was dissolved in dry Et_2O (26 mL) and cooled to -78 °C. At this temperature, *n*-BuLi (1.61 M in hexanes, 3.99 mL, 6.42 mmol, 1.3 equiv) was added dropwise over 50 min (0.08 mL/min) to the colorless solution. After completion of the addition, the resulting yellow solution was stirred at -78 °C for 80 min. Then, a solution of crude **SI-6** (containing a maximum

of 2.42 g, 4.94 mmol, 1.0 equiv) in dry Et_2O (10 mL) was added dropwise (0.1 mL/min). The resulting pale-yellow solution was stirred at -78 °C for 4 h and then allowed to warm to rt. After further 13 h (overnight), the reaction mixture was filtered through a pad of silica [~40 mm depth of wetted (Et_2O) silica, using a filter frit connected directly to a round bottom flask] to give a pale-yellow solution. The silica was washed with Et_2O (80 mL). The filtrate was concentrated under reduced pressure to give 4.49 g of a pale-yellow oil. The crude residue was purified by column chromatography (SiO_2, pent:Et_2O 50:1 to 25:1) to afford **SI-7** (1.55 g, 2.99 mmol, 61%) as a colorless oil.

Remark: The purified fraction contained ethyl 2,4,6-triisopropylbenzoate as an inseparable impurity. The purest fractions (prod:TIBOEt 1:0.09) were used to perform the characterization. The purity of the different collected fractions was determined by ^{1}H-NMR analysis. For the following steps, a yield of 100% was assumed. At the end of the sequence (after the Zweifel olefination) the isolated yield over 6 steps was determined and full characterization was performed.

TLC (SiO_2; pent:Et_2O 25:1, $KMnO_4$, UV): R_f = 0.25. **$[\alpha]_D^{20}$** = +2.45 (c = 2.77 in DCM). **^{1}H NMR** (400 MHz, $CDCl_3$) δ 7.58 – 7.49 (m, 2H), 7.36 – 7.27 (m, 3H), 3.43 (dd, *J* = 9.6, 6.2 Hz, 1H), 3.27 (dd, *J* = 9.6, 8.3 Hz, 1H), 1.91 (dqd, *J* = 8.6, 6.7, 2.3 Hz, 1H), 1.86 – 1.75 (m, 1H), 1.45 (ddd, *J* = 12.4, 8.6, 5.5 Hz, 1H), 1.22 (d, *J* = 1.2 Hz, 12H), 1.19 – 1.05 (m, 3H), 1.00 (d, *J* = 6.9 Hz, 3H), 0.94 – 0.85 (m, 15H), 0.51 (q, *J* = 7.8 Hz, 6H), 0.40 (s, 3H), 0.37 (s, 3H). **^{13}C NMR** (101 MHz, $CDCl_3$) δ 141.1, 134.1, 128.5, 127.7, 82.8, 68.1, 42.4, 36.3, 34.2, 31.0, 25.0, 24.9, 21.1, 16.3, 15.9, 14.9, 7.0, 4.6, 0.5, -0.8. **IR** (thin film): υ = 2954, 2875, 1728, 1462, 1379, 1315, 1248, 1144, 1091, 808, 732, 701 cm^{-1}. **HRMS** (MALDI): calc. for $C_{29}H_{55}BO_3Si_2Na$ $[M+Na]^+$: 541.3681 m/z; found: 541.3694 m/z. (see NMR spectra)

2.3.8. (((2*R*,3*S*,4*R*,6*R*)-3-(dimethyl(phenyl)silyl)-2,4,6-trimethyl-7-(4,4,5,5-tetramethyl-1,3,2-dioxaborolan-2-yl)heptyl)oxy)triethylsilane (SI-8)

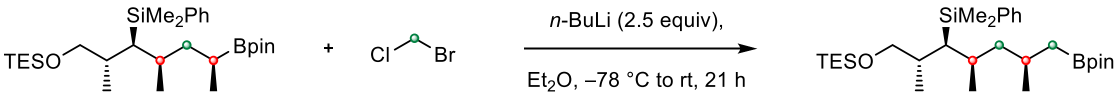

SI-7 bromochloromethane (3.0 equiv) **SI-8**

In a flame-dried Schlenk flask, crude **SI-7** (coevaporated with toluene (50 mL), containing a maximum of 2.56 g, 4.94 mmol, 1.0 equiv) was dissolved under a N_2-atmosphere in Et_2O (60 mL). Bromochloromethane (0.96 mL, 1.92 g, 14.8 mmol, 3.0 equiv) was added and the resulting colorless solution was cooled to -78 °C. Then *n*-BuLi (1.61 M in hexanes, 7.67 mL, 12.4 mmol, 2.5 equiv) was added slowly (1.7 mL/h). The resulting colorless solution was stirred for another 1.5 h at -78 °C and was then allowed to warm to rt. After further 15 h (overnight), the reaction mixture (pale-yellow suspension) was filtered through pad of silica [~40 mm depth of wetted (Et_2O) silica, using a filter frit connected directly to a round bottom flask] to give a pale-yellow solution. The silica was washed with Et_2O (140 mL). The filtrate was concentrated under reduced pressure to give 2.31 g of a pale-yellow oil. The crude mixture was used directly for step 2.3.9.

2.3.9. (((2*R*,3*S*,4*R*,6*R*,8*S*)-3-(dimethyl(phenyl)silyl)-2,4,6-trimethyl-8-(4,4,5,5-tetramethyl-1,3,2-dioxaborolan-2-yl)nonyl)oxy)triethylsilane (28)

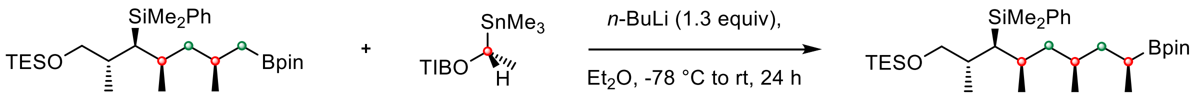

SI-8 **25** (1.3 equiv) **28**

In a flame-dried Schlenk flask, stannane **25**[3] (2.82 g, 6.42 mmol, 1.3 equiv) was dissolved in dry Et_2O (26 mL) and cooled to -78 °C. At this temperature, *n*-BuLi (1.61 M in hexanes, 3.99 mL, 6.42 mmol, 1.3 equiv) was added dropwise over 45 min (0.1 mL/min) to the colorless solution with white particles. After completion of the addition, the resulting yellow solution was stirred at -78 °C for 95 min. Then, a solution of **SI-8** (coevaporated with toluene (50 mL), containing a maximum of 2.63 g, 4.94 mmol, 1.0 equiv) in dry Et_2O (10 mL) was added dropwise (0.1 mL/min). The resulting pale-yellow solution was stirred at -78 °C for 2.5 h and then allowed to warm to rt. After further 18 h (overnight), the reaction mixture was filtered through a pad of silica [~40 mm depth of wetted (Et_2O) silica, using a filter frit connected directly to a round bottom flask] to give a yellow solution. The silica was washed with Et_2O (20 mL). The filtrate was concentrated under reduced pressure to give 3.33 g of a yellow oil. The crude mixture was used directly for step 2.3.10.

2.3.10. (((2*R*,3*S*,4*R*,6*R*,8*S*)-3-(dimethyl(phenyl)silyl)-2,4,6,8-tetramethyldec-9-en-1-yl)oxy)triethylsilane (29)

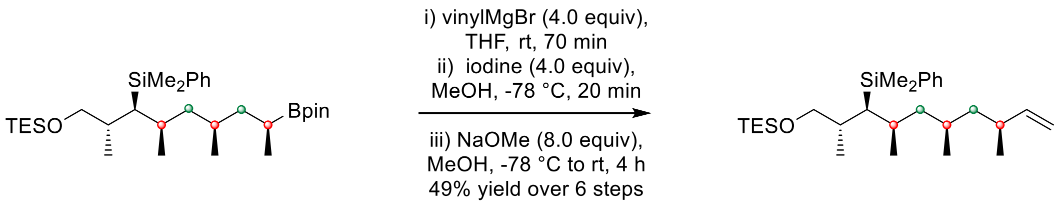

28 **29**

In a flame-dried Schlenk flask, crude **28** (4/5 of the crude material of **28** obtained from the previous step, coevaporated with toluene (40 mL), containing a maximum of 2.22 g, 3.95 mmol, 1.0 equiv) was dissolved in dry THF (40 mL). Vinyl magnesium bromide (0.89 M in THF, 17.8 mL, 15.8 mmol, 4.0 equiv) was added dropwise (1.0 mL/min) at rt to the yellow solution. The resulting red-brown solution was stirred for 70 min at rt and then placed in a cooling bath at -78 °C. After 15 min, a solution of iodine (4.01 g, 15.8 mmol, 4.0 equiv) in dry MeOH (52 mL) was added dropwise (1.0 mL/min) to the reaction mixture. The black solution was stirred for 20 min at -78 °C. Then, NaOMe (0.5 M in MeOH, 63.2 mL, 31.6 mmol, 8.0 equiv) was added dropwise (1.0 mL/min). The black solution was stirred for further 30 min at -78 °C, and for 2.5 h at rt. Then, sat. aq. $Na_2S_2O_3$ solution (35 mL) was added to the reaction mixture. The color changed from dark brown to light yellow. Et_2O (400 mL) and water (400 mL) were added, and the layers were separated. The aq. layer was extracted with Et_2O (3x 400 mL). The org. layers were washed with brine (400 mL), combined, dried over $MgSO_4$, filtrated, and concentrated under reduced pressure to give 2.57 g of a yellow-brown oil. The crude residue was purified by flash chromatography (SiO_2, pentane:Et_2O:NEt_3 100:0:0.5 to 100:1:0.5) to afford **29** (887 mg, 1.92 mmol, 49% over 6 steps) as a yellow oil.

Remark: Without the addition of NEt_3 to the column chromatography eluent, a significant amount of TES-deprotected byproduct was observed.

TLC (SiO_2; Pent:Et_2O 5:1, $KMnO_4$): R_f = 0.27. **$[\alpha]_D^{24}$** = +4.14 (c = 1.45 in MeOH). **^{1}H NMR** (500 MHz, $CDCl_3$) δ 7.57 – 7.48 (m, 2H), 7.34 – 7.29 (m, 3H), 5.62 (ddd, *J* = 17.2, 10.3, 8.0 Hz, 1H), 5.00 – 4.87 (m, 2H), 3.38 (dd, *J* = 9.7, 6.8 Hz, 1H), 3.26 (dd, *J* = 9.7, 7.7 Hz, 1H), 2.25 – 2.14 (m, 1H), 1.95 – 1.85 (m, 1H), 1.85 – 1.76 (m, 1H), 1.52 – 1.44 (m, 1H), 1.23 – 1.10 (m, 3H), 0.98 (d, *J* = 6.7 Hz, 6H), 0.96 – 0.86 (m, 14H), 0.80 (d, *J* = 6.4 Hz, 3H), 0.50 (q, *J* = 8.0 Hz, 6H), 0.39 (s, 3H), 0.37 (s, 3H). **^{13}C NMR** (126 MHz, $CDCl_3$) δ 145.0, 141.0, 134.1, 128.6, 127.7, 112.6, 67.8, 46.9, 44.4, 36.2, 35.6, 32.7, 28.6, 27.8, 22.1, 21.5, 20.4, 15.7, 7.0, 4.6, 0.6, -0.8. **IR** (thin film): υ = 2958, 2875, 1727, 1461, 1353, 1249, 1075, 1011 cm^{-1}. **HRMS** (MALDI): calc. for $C_{28}H_{52}OSi_2Na$ $[M+Na]^+$: 483.3449 m/z; found: 483.3455 m/z. (see NMR spectra)

2.3.11. (((2*R*,3*S*,4*R*,6*R*,8*R*)-3-(dimethyl(phenyl)silyl)-2,4,6,8-tetramethyldecyl)oxy)triethylsilane (SI-9)

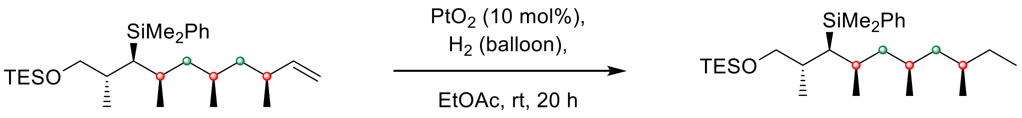

29 **SI-9**

In a 50 mL round bottom flask, olefin **29** (222 mg, 0.481 mmol, 1.0 equiv) was dissolved in dry EtOAc (10 mL) after which PtO_2 (10.9 mg, 0.048 mmol, 10 mol%) was added. The reaction vessel was then subjected to three cycles of evacuation followed by venting with N_2. Then, the reaction vessel was subjected to three cycles of evacuation followed by venting with H_2 (balloon). The black suspension was subsequently stirred under a H_2 atmosphere (balloon) for 20 h, before the mixture was filtered through a Celite pad (2 cm). The residue was washed with EtOAc (200 mL). The filtrate was concentrated under reduced pressure to give crude **SI-9** (385 mg) as a pale-yellow oil. The crude mixture was used directly for step 2.3.12.

2.3.12. (2*R*,3*S*,4*R*,6*R*,8*R*)-2,4,6,8-tetramethyldecane-1,3-diol (30)

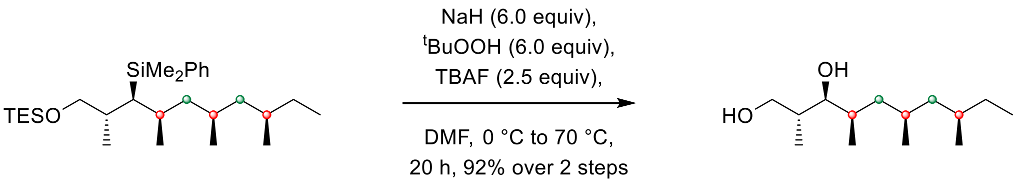

SI-9 **30**

In a flame-dried Schlenk flask, NaH (60%, 116 mg, 2.89 mmol, 6.0 equiv) was suspended in dry DMF (6 mL) and cooled to 0 °C. ^{t}BuOOH (5.0 - 6.0 M in decane, 577 μL, 2.89 mmol, 6.0 equiv) was added dropwise (gas evolution). After the addition, the reaction mixture was allowed to warm to rt, before a solution of **SI-9** (containing a maximum of 223 mg, 0.481 mmol, 1.0 equiv) in DMF (12 mL) was added dropwise at rt. After 10 min, TBAF (1 M in THF, 1.20 mL, 1.20 mmol, 2.5 equiv) was added dropwise at rt. The reaction mixture was heated to 70 °C with an oil bath and stirred at that temperature overnight (Schlenk tap closed). After a total of 20 h, the pale-yellow solution was cooled to 0 °C, quenched with sat. aq. $Na_2S_2O_3$ solution (60 mL) and diluted with EtOAc (200 mL) and water (100 mL). The layers were separated. The aq. layer was extracted with EtOAc (2x 200 mL). The org. layers were washed with water (3x 150 mL), then with brine (150 mL), combined, dried over

$MgSO_4$, filtered, and concentrated under reduced pressure to give 411 mg of a yellow oil. The crude residue was purified by flash chromatography (SiO_2, pentane:Et_2O 1:2) to afford **30** (102 mg, 0.441 mmol, 92% over 2 steps) as a pale-yellow oil.

TLC (SiO_2; Pent:Et_2O 1:3, $KMnO_4$): R_f = 0.36. $[\alpha]_D^{24}$ = -14.0 (c = 3.14 in $CHCl_3$). **^{1}H NMR** (500 MHz, CD_3OD) δ 3.73 (ddd, *J* = 10.5, 5.2, 2.1 Hz, 1H), 3.59 – 3.52 (m, 1H), 3.35 – 3.30 (m, 1H), 1.76 (dddd, *J* = 16.3, 11.1, 8.1, 4.8 Hz, 2H), 1.63 (tdd, *J* = 12.0, 5.9, 2.9 Hz, 1H), 1.53 – 1.36 (m, 3H), 1.27 (ddd, *J* = 15.5, 7.5, 5.8 Hz, 1H), 1.15 – 1.04 (m, 1H), 1.02 – 0.96 (m, 1H), 0.97 – 0.92 (m, 1H), 0.92 – 0.84 (m, 15H). **^{13}C NMR** (126 MHz, CD_3OD) δ 77.2, 66.9, 46.3, 43.2, 39.6, 33.0, 32.9, 30.0, 28.5, 21.1, 20.4, 14.1, 13.6, 11.5. **IR** (thin film): υ = 3342, 2958, 2912, 2875, 1461, 1379, 1097, 1026, 978 cm^{-1}. **HRMS** (ESI): calc. for $C_{14}H_{30}O_2Na$ $[M+Na]^+$: 253.2143 m/z; found: 253.2148 m/z. (see NMR spectra)

2.3.13. (2*S*,3*S*,4*R*,6*R*,8*R*)-3-hydroxy-2,4,6,8-tetramethyldecanal (SI-10)

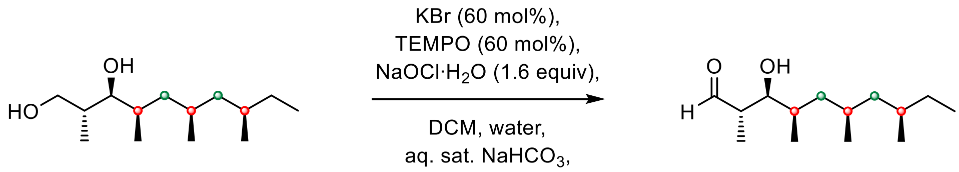

30 **SI-10**

In a 250 mL round bottom flask, diol **30** (55.6 mg, 0.241 mmol, 1.0 equiv) was dissolved in DCM (18 mL). To the pale-yellow solution KBr (8.6 mg, 0.072 mmol, 30 mol%) and aq. sat $NaHCO_3$ (9.0 mL) were added. The reaction mixture was cooled to 0 °C, before TEMPO (11.3 mg, 0.072 mmol, 30 mol%) was added, followed by NaOCl·$5H_2O$ (43.7 mg, 0.265 mmol, 1.1 equiv) dissolved in water (27 mL). The resulting emulsion was stirred vigorously at 0 °C. After 19 h, additional KBr (8.6 mg, 0.072 mmol, 30 mol%) and TEMPO (11.3 mg, 0.072 mmol, 30 mol%), followed by NaOCl·$5H_2O$ (19.8 mg, 0.121 mmol, 0.5 equiv) dissolved in water (5 mL) was added to the emulsion. After a total of 21 h, sat. aq. $Na_2S_2O_3$ (20 mL), sat. aq. $NaHCO_3$ (30 mL), and additional DCM (40 mL) were added to the pale-yellow emulsion and the layers were separated. The aq. layer was extracted with DCM (2x 40 mL). The org. layers were washed with brine (1x 40 mL), combined, dried over $MgSO_4$, filtered, and concentrated under reduced pressure to give crude **SI-10** (87.4 mg) as a pale-yellow-orange oil. The crude mixture was used directly for step 2.3.14.

2.3.14. (2*S*,3*S*,4*R*,6*R*,8*R*)-3-hydroxy-2,4,6,8-tetramethyldecanoic acid (SI-11)

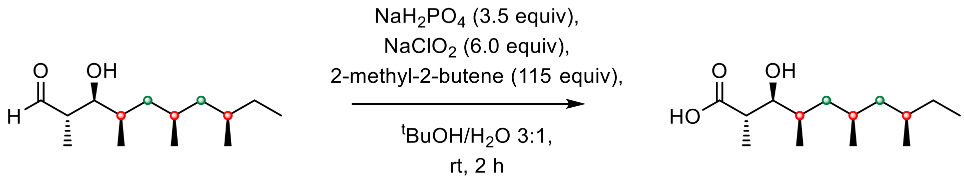

SI-10 **SI-11**

In a 25 mL round bottom flask, NaH_2PO_4 (101 mg, 0.844 mmol, 3.5 equiv) and $NaClO_2$ (131 mg, 1.45 mmol, 6.0 equiv) were suspended in ^{t}BuOH (8.0 mL). 2-Methyl-2-butene (2.94 mL, 1.95 g, 27.7 mmol, 115 equiv), followed by water (3 mL) was added and the resulting colorless solution was stirred at rt for 10 min. Then the solution was transferred to another 25 mL flask containing crude **SI-10** (containing a maximum of 55.1 mg, 0.241 mmol, 1.0 equiv). The resulting yellow solution was stirred at rt for 2 h, before EtOAc (40 mL) and water

(40 mL) were added, and the layers were separated. The aq. layer was extracted with EtOAc (2x 40 mL). The org. layers were washed with brine (40 mL), combined, dried over $MgSO_4$, filtered, and concentrated under reduced pressure to obtain crude **SI-11** (80.8 mg) as a yellow oil. The crude mixture was used directly for step 2.3.15.

2.3.15. allyl (2*S*,3*S*,4*R*,6*R*,8*R*)-3-hydroxy-2,4,6,8-tetramethyldecanoate (31)

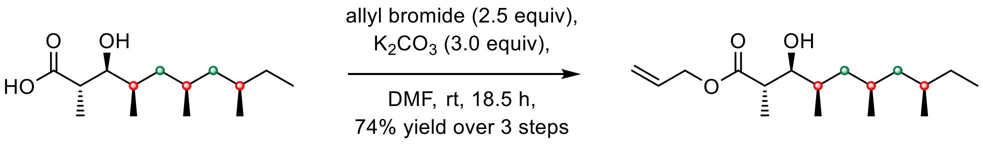

SI-11 **31**

In a 25 mL round bottom flask, crude **SI-11** (containing a maximum of 59 mg, 0.241 mmol, 1.0 equiv) was dissolved in dry DMF (6 mL). K_2CO_3 (101 mg, 0.724 mmol, 3.0 equiv), followed by allyl bromide (52 µL, 73 mg, 0.604 mmol, 2.5 equiv) was added to the pale-yellow solution and the resulting suspension was stirred at rt overnight (18.5 h), before water (30 mL) and EtOAc (30 mL) were added to the reaction mixture and the layers were separated. The aq. layer was extracted with EtOAc (2x 40 mL). The org. layers were washed with water (40 mL), brine (40 mL), combined, dried over $MgSO_4$, filtered, and concentrated under reduced pressure to obtain 188.9 mg of an orange-brown oil. The crude residue was purified by flash chromatography (SiO_2, pentane:Et_2O 6:1) to afford **31** (50.5 mg, 0.178 mmol, 74% over 3 steps) as a colorless oil.

TLC (SiO_2; Pent:Et_2O 4:1, CAM): R_f = 0.37. **$[\alpha]_D^{22}$** = +9.58 (c = 2.71 in $CHCl_3$). **^{1}H NMR** (400 MHz, $CDCl_3$) δ 5.92 (ddt, *J* = 17.2, 10.4, 5.7 Hz, 1H), 5.33 (dq, *J* = 17.2, 1.5 Hz, 1H), 5.24 (dq, *J* = 10.4, 1.3 Hz, 1H), 4.61 (dt, *J* = 5.8, 1.4 Hz, 2H), 3.61 (ddd, *J* = 8.3, 5.9, 3.3 Hz, 1H), 2.65 (dq, *J* = 8.3, 7.2 Hz, 1H), 2.39 (d, *J* = 6.0 Hz, 1H), 1.71 (hd, *J* = 6.9, 3.3 Hz, 1H), 1.65 – 1.52 (m, 1H), 1.49 – 1.29 (m, 3H), 1.26 – 1.18 (m, 1H), 1.16 (d, *J* = 7.2 Hz, 3H), 1.10 – 0.88 (m, 3H), 0.88 – 0.81 (m, 12H). **^{13}C NMR** (101 MHz, $CDCl_3$) δ 176.3, 132.1, 118.6, 75.2, 65.4, 44.9, 43.4, 41.6, 31.9, 31.6, 29.1, 27.3, 20.8, 20.0, 14.5, 13.5, 11.3. **IR** (thin film): υ = 3522, 2959, 2925, 2876, 1720, 1459, 1379, 1252, 1171, 984, 929 cm^{-1}. **HRMS** (ESI): calc. for $C_{17}H_{32}O_3Na$ $[M+Na]^+$: 307.2244 m/z; found: 307.2252 m/z. (see NMR spectra)

2.4. Building Block Assembly

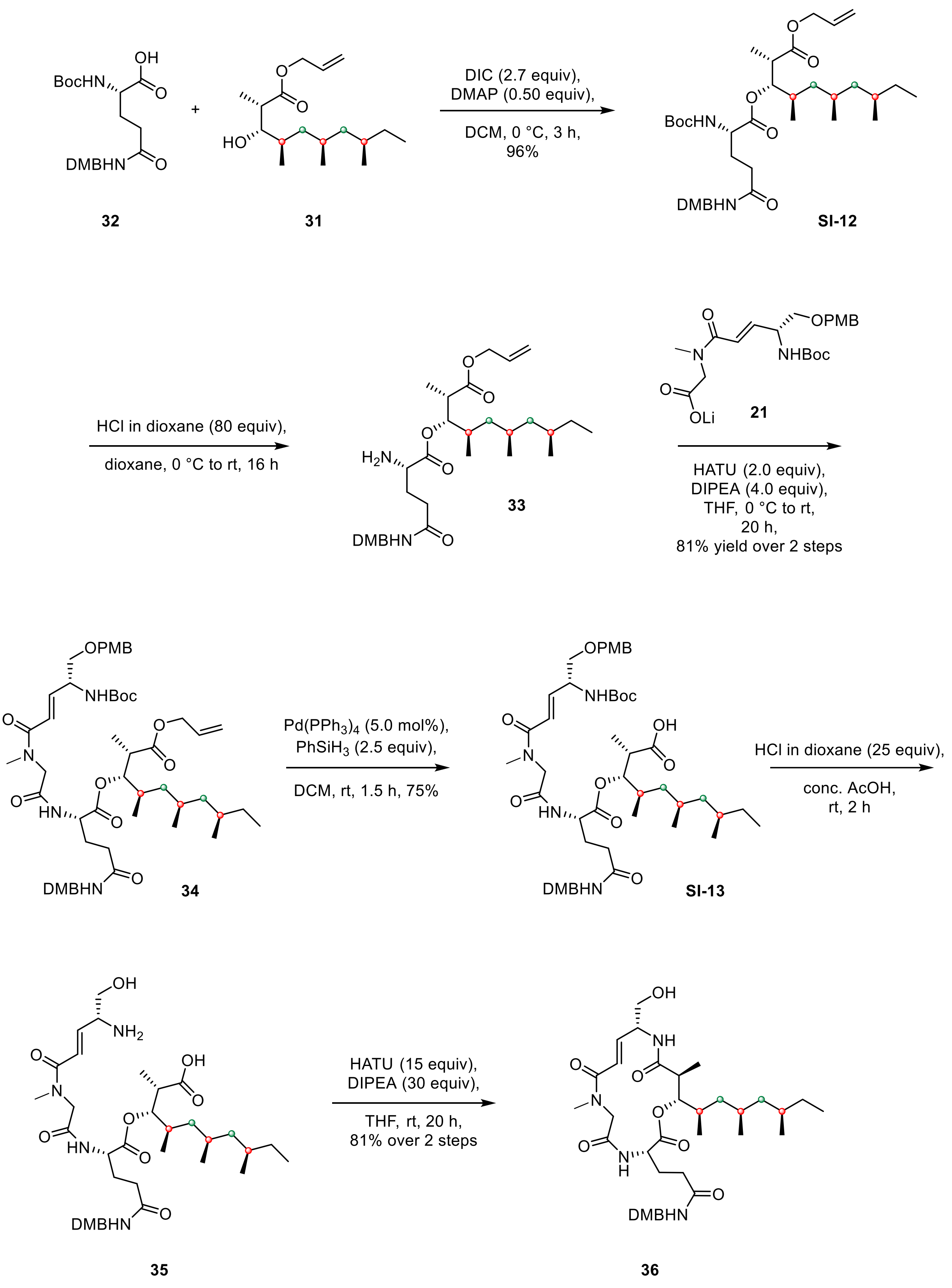

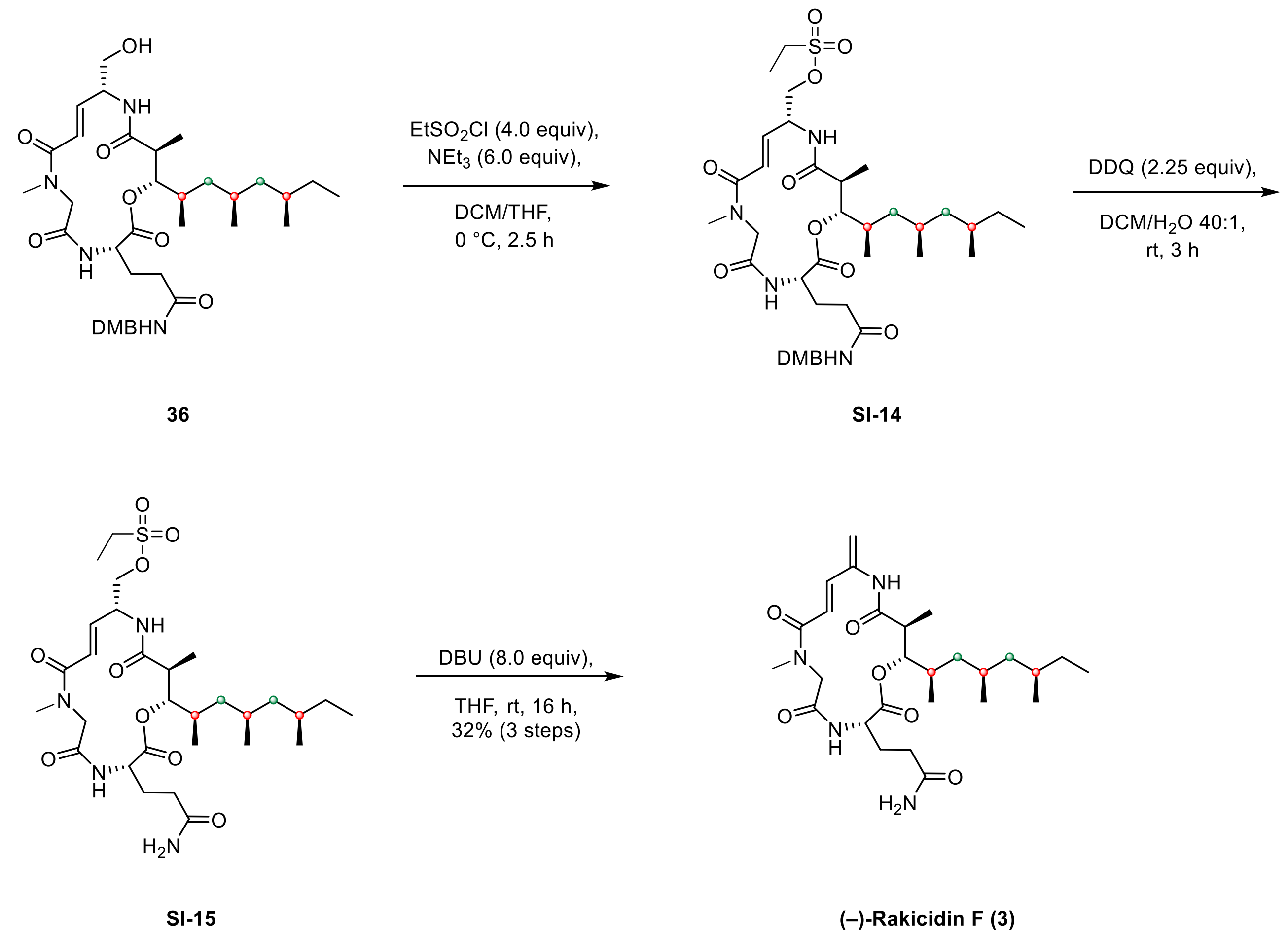

2.4.1. allyl (2*S*,3*S*,4*R*,6*R*,8*R*)-3-((*N*[2]-(*tert*-butoxycarbonyl)-*N*[5]-(2,4-dimethoxybenzyl)-*L*-glutaminyl)oxy)-2,4,6,8-tetramethyldecanoate (SI-12)

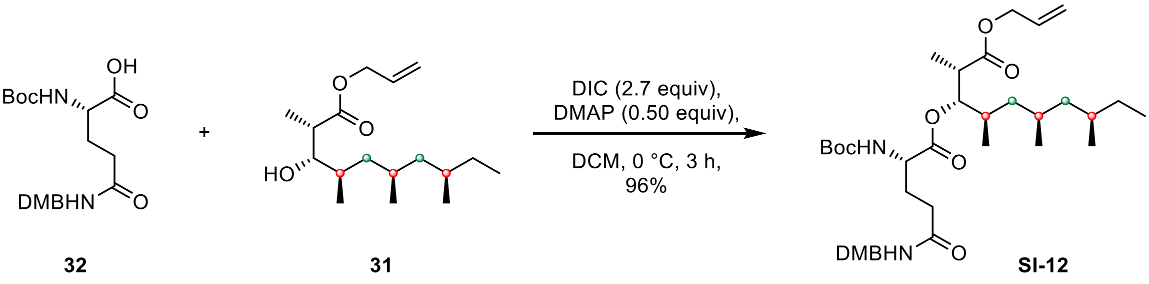

In a 25 mL round bottom flask, alcohol **31** (48.8 mg, 172 µmol, 1.0 equiv) and acid **32** (170 mg, 429 µmol, 2.5 equiv) were dissolved in dry DCM (3.0 mL). DMAP (10.5 mg, 85.8 µmol, 0.5 equiv) was added and the resulting solution was cooled to 0 °C. Then, DIC (72 µL, 58.5 mg, 463 µmol, 2.7 equiv) was added. The resulting pale-yellow solution was stirred at 0 °C for 3 h, before DCM (40 mL) and sat. aq. $NaHCO_3$ (40 mL) were added to the resulting white suspension. The layers were separated. The aq. layer was extracted with DCM (3x 40 mL). The org. layers were washed with brine (40 mL), combined, dried over $MgSO_4$, filtered, and concentrated under reduced pressure to give 242 mg of an off-white oily solid. The crude residue was purified by column chromatography (SiO_2, pent:Et_2O 1:2) to afford **SI-12** (109 mg, 0.164 mmol, 96%) as a pale-yellow oil.

TLC (SiO_2; pent:Et_2O 1:3, CAM, UV): R_f = 0.29. **$[\alpha]_D^{22}$** = +4.82 (c = 5.31 in $CHCl_3$). **^{1}H NMR** (400 MHz, $CDCl_3$) δ 7.17 (d, *J* = 8.1 Hz, 1H), 6.45 – 6.38 (m, 2H), 6.35 (s, 1H), 5.92 – 5.78 (m, 1H), 5.28 (dq, *J* = 17.2, 1.5 Hz, 1H), 5.24 – 5.17 (m, 2H), 5.12 (dd, *J* = 8.9, 3.1 Hz, 1H), 4.53 – 4.41 (m, 2H), 4.35 (d, *J* = 5.7 Hz, 2H), 4.27 – 4.18 (m, 1H), 3.81 (s, 3H), 3.78 (s, 3H), 2.80 (dq, *J* = 9.0, 7.2 Hz, 1H), 2.31 – 2.04 (m, 3H), 1.97 – 1.80 (m, 2H), 1.62 (dq, *J* = 14.4, 7.3 Hz, 1H), 1.42 (s, 9H), 1.40 – 1.29 (m, 2H), 1.27 – 1.18 (m, 1H), 1.18 – 1.10 (m, 1H), 1.15 (d, *J* = 7.1 Hz, 3H), 1.07 – 0.95 (m, 1H), 0.94 – 0.76 (m, 14H). **^{13}C NMR** (101 MHz, $CDCl_3$) δ 173.8, 171.6 (2x), 160.6, 158.7, 155.6, 132.1, 130.6, 119.1, 118.7, 104.0, 98.7, 79.9, 77.9, 65.6, 55.5, 55.4, 53.2, 44.7, 42.3, 41.6, 39.0, 32.7, 31.5, 31.1, 29.0, 28.7, 28.4, 27.1, 20.7, 20.1, 14.3, 14.0, 11.2. **IR** (thin film): υ = 3321, 2961, 2930, 1715, 1650, 1507, 1457, 1366, 1288, 1253, 1208, 1157 cm^{-1}. **HRMS** (ESI): calc. for $C_{36}H_{59}N_2O_9$ $[M+H]^+$: 663.4215 m/z; found: 663.4223 m/z. (see NMR spectra)

2.4.2. (*S*)-1-(((2*S*,3*S*,4*R*,6*R*,8*R*)-1-(allyloxy)-2,4,6,8-tetramethyl-1-oxodecan-3-yl)oxy)-5-((3,4-dimethylbenzyl)amino)-1,5-dioxopentan-2-aminium chloride (33)

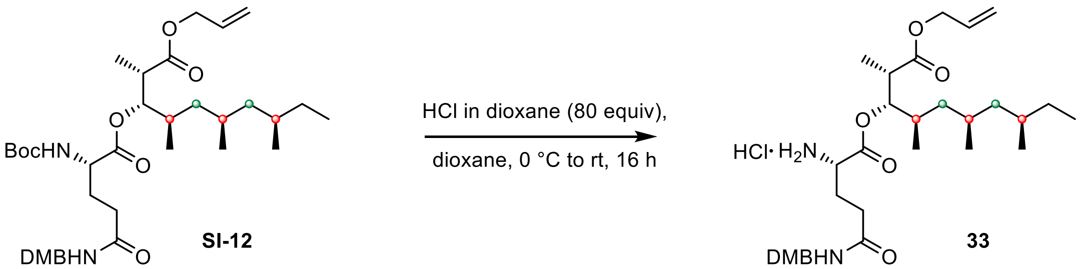

SI-12 **33**

In a 25 mL round bottom flask, **SI-12** (150 mg, 226 μmol, 1.0 equiv) was dissolved in dry dioxane (13 mL) and cooled to 0 °C. Then, HCl (4N in dioxane, 4.53 mL, 18.1 mmol, 80 equiv) was added dropwise. The resulting colorless solution was stirred at 0 °C for 15 min and then allowed to warm to rt. After a total 16 h, the reaction mixture was concentrated under reduced pressure to obtain crude **33** (207 mg) as a colorless oil. The crude mixture was used directly for step 2.4.3.

2.4.3. (2*S*,3*S*,4*R*,6*R*,8*R*)-1-(allyloxy)-2,4,6,8-tetramethyl-1-oxodecan-3-yl (6*R*,14*S*,*E*)-14-(3-((2,4-dimethoxybenzyl)amino)-3-oxopropyl)-6-(((4-methoxybenzyl)oxy)methyl)-2,2,10-trimethyl-4,9,12-trioxo-3-oxa-5,10,13-triazapentadec-7-en-15-oate (34)

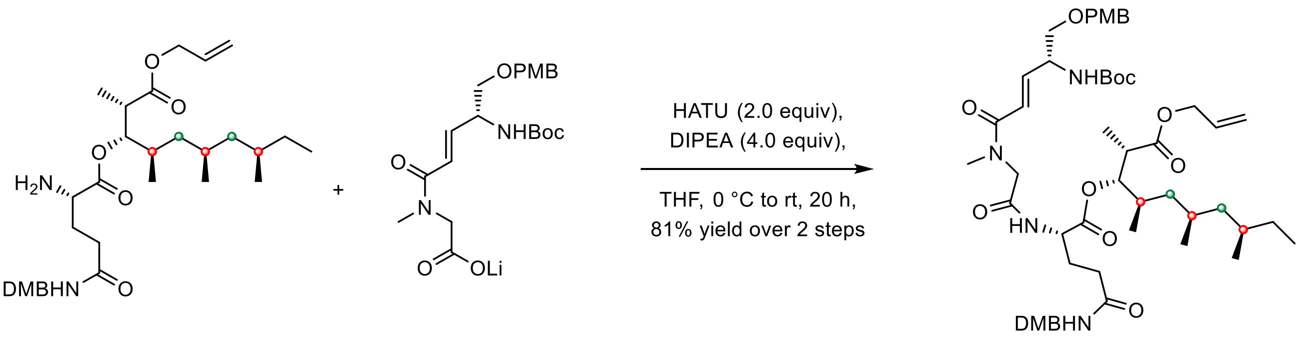

33 **21** **34**

In a 25 mL round bottom flask, carboxylate **21** (145.4 mg, 339 μmol, 1.5 equiv) and crude **33** (containing a maximum of 127 mg, 226 μmol, 1.0 equiv) were dissolved in dry THF (10 mL). DIPEA (158 μL, 117 mg, 905 μmol, 4.0 equiv) was added and the resulting colorless solution was cooled to 0 °C. HATU (172 mg, 452 μmol,

2.0 equiv) was added. After stirring for 30 min at 0 °C, the colorless solution was allowed to warm to rt. After a total of 20 h, sat. aq. $NaHCO_3$ (40 mL) and EtOAc (40 mL) were added to the reaction mixture and the layers were separated. The aq. layer was extracted with EtOAc (2x 40 mL). The org. layers were washed with brine (1x 40 mL), combined, dried over $MgSO_4$, filtered, and concentrated under reduced pressure to give 462 mg of an orange oil. The crude residue was purified by column chromatography (SiO_2, DCM:MeOH 98:2 to 95:5) to afford **34** (177 mg, 0.183 mmol, 81% over 2 steps) as a yellow-orange foam.

TLC (SiO_2; DCM:MeOH 10:1, CAM, UV): R_f = 0.38. **$[\alpha]_D^{22}$** = -4.98 (c = 4.58 in MeOH). Mixture of rotamers, ^1H-NMR data is reported for both rotamers. **^{1}H NMR** (500 MHz, CD_3OD) δ 7.29 – 7.18 (m, 2H), 7.15 – 7.09 (m, 1H), 6.91 – 6.83 (m, 2H), 6.78 – 6.63 (m, 1H), 6.60 – 6.32 (m, 3H), 5.97 – 5.84 (m, 1H), 5.36 – 5.26 (m, 1H), 5.25 – 5.16 (m, 1H), 5.16 – 5.07 (m, 1H), 4.59 – 4.33 (m, 6H), 4.31 – 4.22 (m, 2H), 4.21 – 3.97 (m, 2H), 3.82 – 3.73 (m, 9H), 3.47 (d, *J* = 6.0 Hz, 2H), 3.15 (s, 2H), 3.03 – 2.95 (m, 1H), 2.91 – 2.80 (m, 1H), 2.37 – 2.24 (m, 2H), 2.22 – 1.79 (m, 3H), 1.76 – 1.60 (m, 1H), 1.51 – 1.34 (m, 11H), 1.33 – 1.25 (m, 1H), 1.23 – 1.17 (m, 1H), 1.15 (d, *J* = 7.1 Hz, 3H), 1.10 – 0.99 (m, 1H), 0.95 – 0.77 (m, 14H). Mixture of rotamers, ^{13}C-NMR data is reported for the major rotamer. **^{13}C NMR** (126 MHz, CD_3OD) δ 175.3, 174.3, 172.3, 170.9, 169.1, 162.0, 160.8, 159.8, 157.7, 144.8, 133.5, 131.3, 130.8, 130.6, 122.0, 119.7, 118.8, 114.8, 105.3, 99.3, 80.5, 78.7, 73.8, 72.2, 66.6, 55.9, 55.8, 55.7, 53.3, 53.0, 52.2, 46.1, 43.4, 42.7, 39.3, 37.7, 33.2, 32.6, 32.2, 29.8, 28.7, 28.6, 28.2, 20.7, 20.6, 14.6, 14.5, 11.5. **IR** (thin film): υ = 3301, 2961, 2932, 1662, 1614, 1511, 1248, 1172, 1035 cm^{-1}. **HRMS** (ESI): calc. for $C_{52}H_{79}N_4O_{13}$ $[M+H]^+$: 967.5638 m/z; found: 967.5657 m/z. (see NMR spectra)

2.4.4. (6*R*,14*S*,17*S*,18*S*,*E*)-14-(3-((2,4-dimethoxybenzyl)amino)-3-oxopropyl)-17-((2*R*,4*R*,6*R*)-4,6-dimethyloctan-2-yl)-6-(((4-methoxybenzyl)oxy)methyl)-2,2,10,18-tetramethyl-4,9,12,15-tetraoxo-3,16-dioxa-5,10,13-triazanonadec-7-en-19-oic acid (SI-13)

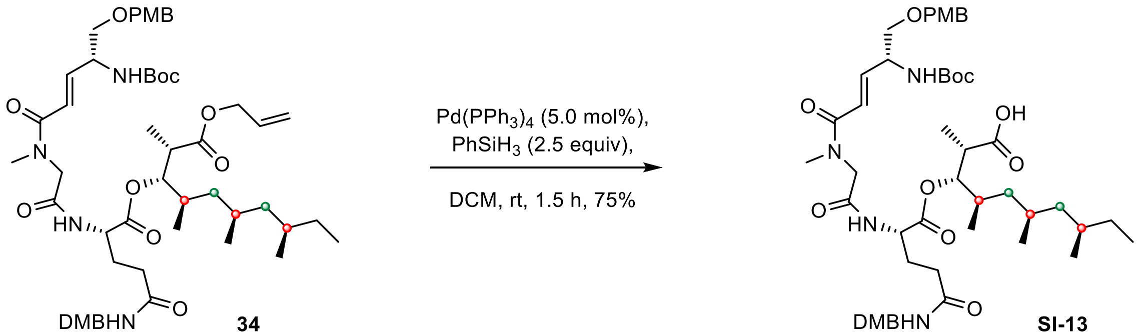

34 **SI-13**

In a 25 mL round bottom flask, allyl ester **34** (66.5 mg, 68.7 μmol, 1.0 equiv) was dissolved in dry DCM (3 mL). Phenylsilane (21.2 μL, 18.6 mg, 172 μmol, 2.5 equiv), followed by $Pd(PPh_3)_4$ (4.0 mg, 3.4 μmol, 5.0 mol%) dissolved in DCM (1 mL) was added. The resulting yellow solution was stirred at rt for 90 min and then directly purified by column chromatography (SiO_2, DCM:MeOH 10:1) to afford **SI-13** (48.0 mg, 51.8 μmol, 75%) as a colorless oil.

TLC (SiO_2; DCM:MeOH 10:1, CAM, UV): R_f = 0.33. **$[\alpha]_D^{22}$** = +5.67 (c = 1.98 in CH_3Cl). Mixture of rotamers, ^1H-NMR data is reported for both rotamers. **^{1}H NMR** (500 MHz, CD_3OD) δ 7.26 – 7.21 (m, 2H), 7.15 – 7.09 (m, 1H), 6.89 – 6.83 (m, 2H), 6.76 – 6.63 (m, 1H), 6.59 – 6.35 (m, 3H), 5.15 (dd, *J* = 9.8, 2.2 Hz, 1H), 4.47 – 4.35 (m, 4H), 4.31 – 4.25 (m, 2H), 4.21 – 4.01 (m, 2H), 3.80 (s, 3H), 3.77 (s, 3H), 3.76 (s, 3H), 3.51 – 3.44 (m, 2H), 3.20 – 2.94 (m, 3H), 2.79 – 2.67 (m, 1H), 2.41 – 2.24 (m, 2H), 2.24 – 2.10 (m, 1H), 2.05 – 1.86 (m, 2H), 1.80 –

1.65 (m, 1H), 1.47 – 1.35 (m, 11H), 1.35 – 1.26 (m, 1H), 1.23 – 1.15 (m, 1H), 1.13 (d, *J* = 6.9 Hz, 3H), 1.10 – 1.01 (m, 1H), 0.94 – 0.81 (m, 14H). Mixture of rotamers, ^{13}C-NMR data is reported for both rotamers separately (major rotamer: MA; minor rotamer: MI). **^{13}C NMR** (126 MHz, CD_3OD) δ 181.0 (MA&MI), 174.8 (MA), 174.6 (MI), 172.2 (MI), 172.2 (MA), 171.0 (MA), 170.5 (MI), 169.5 (MI), 169.2 (MA), 162.1 (MA&MI), 160.9 (MA&MI), 159.8 (MI), 159.8 (MA), 157.8 (MA&MI), 145.0 (MA), 144.4 (MI), 131.3 (MI), 131.3 (MA), 130.9 (MI), 130.8 (MA), 130.6 (MA&MI), 122.4 (MI), 121.9 (MA), 119.6 (MI), 119.5 (MI), 114.8 (MA&MI), 105.3 (MA), 99.3 (MA), 80.5 (MA&MI), 79.8 (MI), 79.5(MA), 73.8 (MA&MI), 72.3 (MI), 72.2 (MA), 55.9 (MA&MI), 55.8 (MA&MI), 55.7 (MA&MI), 53.8 (MI), 53.6 (MA), 53.1 (MA&MI), 52.3 (MA&MI), 46.3 (MA), 46.1 (MI), 44.6 (MA), 44.4 (MI), 43.0 (MA), 39.4 (MA), 39.4 (MI), 37.7 (MA), 35.6 (MI), 33.2 (MI), 33.0 (MA), 32.7 (MA&MI), 32.2 (MI), 32.0 (MI), 29.8 (MA), 29.8 (MI), 28.8 (MI), 28.7 (MA), 28.6 (MA&MI), 28.2 (MI), 28.2 (MA), 20.7 (MI), 20.7 (MI), 20.6 (MA), 20.6 (MA), 14.9 (MA&MI), 14.4 (MA), 14.4 (MI), 11.5 (MA&MI). (MA = major rotamer, MI = minor rotamer) **IR** (thin film): υ = 3302, 2961, 2927, 2873, 1661, 1612, 1510, 1456, 1247, 1158, 1116, 1035 cm^{-1}. **HRMS** (ESI): calc. for $C_{49}H_{75}N_4O_{13}$ $[M+H]^+$: 927.5315 m/z; found: 927.5335 m/z. (see NMR spectra)

2.4.5. 2.4.5. (*R*,*E*)-5-((2-(((*S*)-1-(((2*S*,3*S*,4*R*,6*R*,8*R*)-2-carboxy-4,6,8-trimethyldecan-3-yl)oxy)-5-((3,4-dimethylbenzyl)amino)-1,5-dioxopentan-2-yl)amino)-2-oxoethyl)(methyl)amino)-1-hydroxy-5-oxopent-3-en-2-aminium chloride (35)

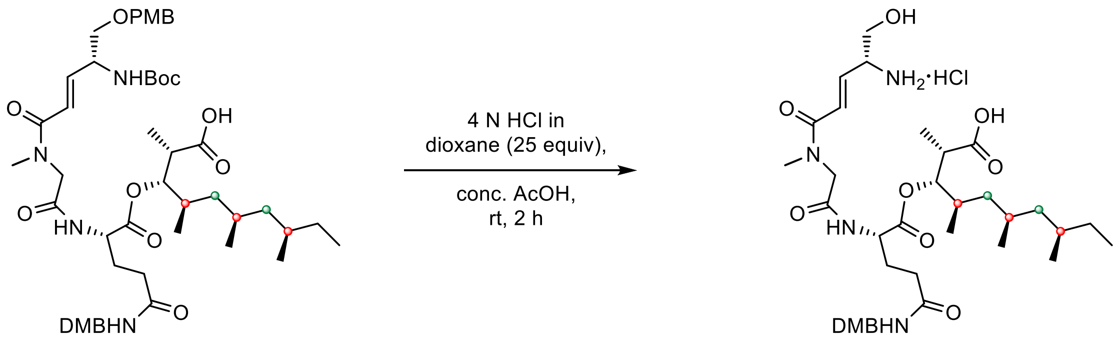

SI-13 **35**

In a 25 mL round bottom flask, **SI-13** (27.6 mg, 29.8 µmol, 1.0 equiv) was dissolved in conc. AcOH (3 mL). To the resulting pale-yellow solution, HCl (4N in dioxane, 186 µL, 0.744 mmol, 25 equiv) was added dropwise. The resulting pale-yellow solution was stirred at rt for 2 h and then concentrated under reduced pressure, re-dissolved twice in dry toluene (10 mL), and concentrated again to obtain crude **35** (26.7 mg) as a pale-yellow oil. The crude mixture was used directly for step 2.4.6.

2.4.6. *N*-(2,4-dimethoxybenzyl)-3-((3*S*,11*R*,14*S*,15*S*,*E*)-15-((2*R*,4*R*,6*R*)-4,6-dimethyloctan-2-yl)-11-(hydroxymethyl)-7,14-dimethyl-2,5,8,13-tetraoxo-1-oxa-4,7,12-triazacyclopentadec-9-en-3-yl)propanamide (36)

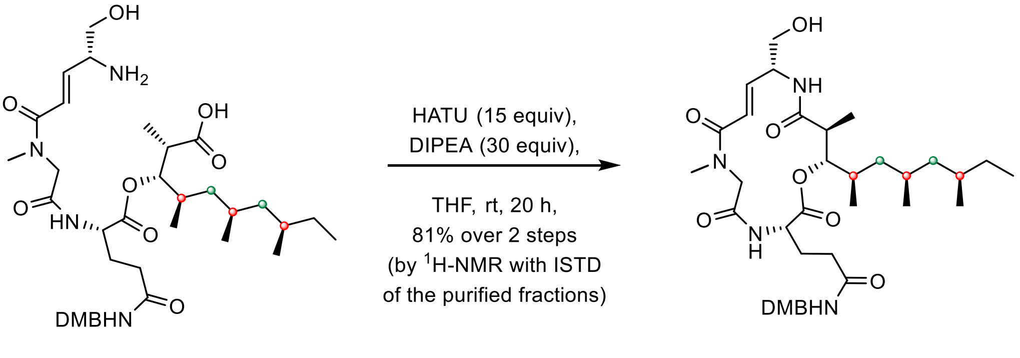

In a 100 mL round bottom flask, HATU (169 mg, 446 µmol, 15 equiv) and DIPEA (155 µL, 891 µmol, 30 equiv) were suspended in dry THF (30 mL). Then, crude **35** (containing a maximum of 21.0 mg, 29.7 µmol, 1.0 equiv) dissolved in THF (15 mL) was added dropwise over 3 h, using a syringe pump (0.08 mL/min). The resulting white suspension was stirred at rt for additional 17 h, before the reaction mixture was concentrated under reduced pressure. To the residue, EtOAc (40 mL) and sat. aq. $NaHCO_3$ (40 mL) were added, and the layers were separated. The aq. layer was extracted with EtOAc (2x 40 mL). The org. layers were washed with brine (40 mL), combined, dried over $MgSO_4$, filtered, and concentrated under reduced pressure to give 92.9 mg of a pale-yellow oil. The crude residue was purified by column chromatography (SiO_2, DCM:MeOH 10:1 to 85:15) to afford **36** (35.2 mg, 47% pure (by ^{1}H-NMR with ISTD (dibromomethane), 24.0 µmol, 81% over 2 steps) as a white solid.

Remark: The impurity could not be seen in the ^{1}H-NMR and ^{13}C-NMR spectra. By ^{19}F-NMR and ^{31}P-NMR analysis, it could be assigned as an inorganic PF_6 salt, probably $NaPF_6$. Analytical data were obtained using the mixture. For the determination of the $[\alpha]_D^{21}$, the calculated purity (by ^{1}H-NMR with ISTD) was used to determine the concentration.

TLC (SiO_2; DCM:MeOH 85:15, CAM, UV): R_f = 0.45. $[\alpha]_D^{21}$ = -67.69 (c = 1.56 in MeOH). **^{1}H NMR** (500 MHz, DMSO-*d*6) δ 8.38 (d, *J* = 9.4 Hz, 1H), 8.01 (t, *J* = 5.8 Hz, 1H), 7.79 (d, *J* = 8.7 Hz, 1H), 7.03 (d, *J* = 8.3 Hz, 1H), 6.76 (dd, *J* = 14.9, 2.9 Hz, 1H), 6.53 (d, *J* = 2.4 Hz, 1H), 6.43 (dd, *J* = 8.3, 2.4 Hz, 1H), 5.95 (dd, *J* = 14.9, 2.3 Hz, 1H), 5.16 (dd, *J* = 10.4, 1.4 Hz, 1H), 4.90 (t, *J* = 5.7 Hz, 1H), 4.41 (dtt, *J* = 8.8, 6.0, 2.4 Hz, 1H), 4.36 – 4.26 (m, 2H), 4.12 (d, *J* = 5.8 Hz, 2H), 3.82 (d, *J* = 17.9 Hz, 1H), 3.76 (s, 3H), 3.73 (s, 3H), 3.43 – 3.35 (m, 2H), 2.95 (s, 3H), 2.81 (dq, *J* = 10.3, 6.9 Hz, 1H), 2.26 – 2.12 (m, 2H), 2.03 – 1.75 (m, 3H), 1.74 – 1.64 (m, 1H), 1.42 – 1.30 (m, 2H), 1.18 – 1.07 (m, 2H), 1.06 – 0.98 (m, 1H), 1.03 (d, *J* = 7.0 Hz, 3H), 0.93 (d, *J* = 6.8 Hz, 3H), 0.81 (q, *J* = 6.8 Hz, 11H). **^{13}C NMR** (126 MHz, DMSO-*d*6) δ 173.0, 170.9, 169.8, 167.9, 165.5, 159.7, 157.7, 143.8, 128.7, 119.0, 118.4, 104.3, 98.2, 75.4, 63.0, 55.4, 55.2, 52.1 (2x), 51.6, 44.7, 41.6, 41.4, 36.8, 36.5, 31.8, 30.9, 30.3, 29.3, 28.2, 26.5, 20.1 (2x), 15.9, 13.8, 10.9. **IR** (thin film): υ = 3646, 3412, 2959, 1634, 1538 cm^{-1}. **HRMS** (ESI): calc. for $C_{36}H_{57}N_4O_9$ $[M+H]^+$: 689.4120 m/z; found: 689.4122 m/z. (see NMR spectra)

2.4.7. ((3*S*,11*R*,14*S*,15*S*,*E*)-3-(3-((2,4-dimethoxybenzyl)amino)-3-oxopropyl)-15-((2*R*,4*R*,6*R*)-4,6-dimethyloctan-2-yl)-7,14-dimethyl-2,5,8,13-tetraoxo-1-oxa-4,7,12-triazacyclopentadec-9-en-11-yl)methyl ethanesulfonate (SI-14)

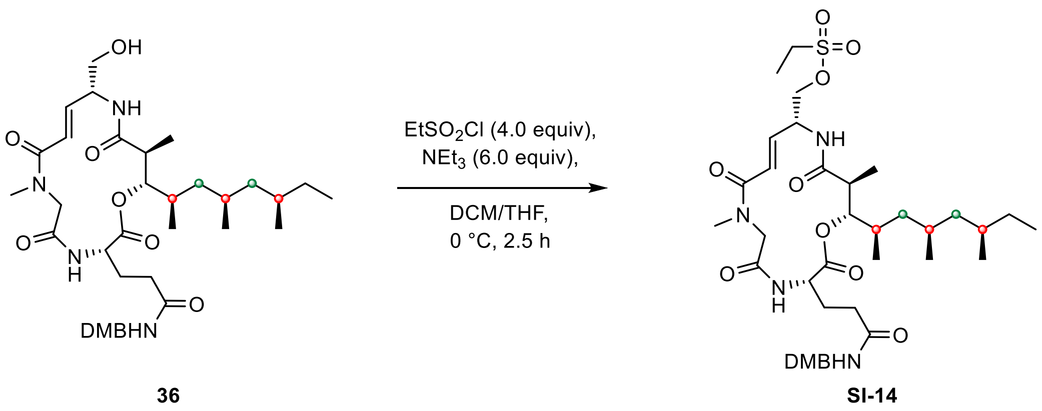

In a 10 mL round bottom flask, a solution of **36** (7.8 mg, 11.4 µmol, 1.0 equiv) and NEt_3 (4.8 µL, 34.1 µmol, 3.0 equiv) in DCM (0.8 mL) and THF (0.5 mL) was cooled to 0 °C. $EtSO_2Cl$ (2.2 µL, 22.7 µmol, 2.0 equiv) dissolved in THF (0.30 mL) was added and the resulting colorless solution was stirred at 0 °C. After 1 h 45 min, additional NEt_3 (4.8 µL, 34.1 µmol, 3.0 equiv) dissolved in THF (0.30 mL) and $EtSO_2Cl$ (2.2 µL, 22.7 µmol, 2.0 equiv) dissolved in THF (0.30 mL) were added. After a total of 2.5 h, sat. aq. $NaHCO_3$ (40 mL) and EtOAc (40 mL) were added to the reaction mixture and the layers were separated. The aq. layer was extracted with EtOAc (2x 40 mL). The org. layers were washed with brine (40 mL), combined, dried over $MgSO_4$, filtered, and concentrated under reduced pressure to give crude **SI-14** (20.1 mg) as a white solid. The crude mixture was used directly for step 2.4.8.

2.4.8. ((3*S*,11*R*,14*S*,15*S*,*E*)-3-(3-amino-3-oxopropyl)-15-((2*R*,4*R*,6*R*)-4,6-dimethyloctan-2-yl)-7,14-dimethyl-2,5,8,13-tetraoxo-1-oxa-4,7,12-triazacyclopentadec-9-en-11-yl)methyl ethanesulfonate (SI-15)

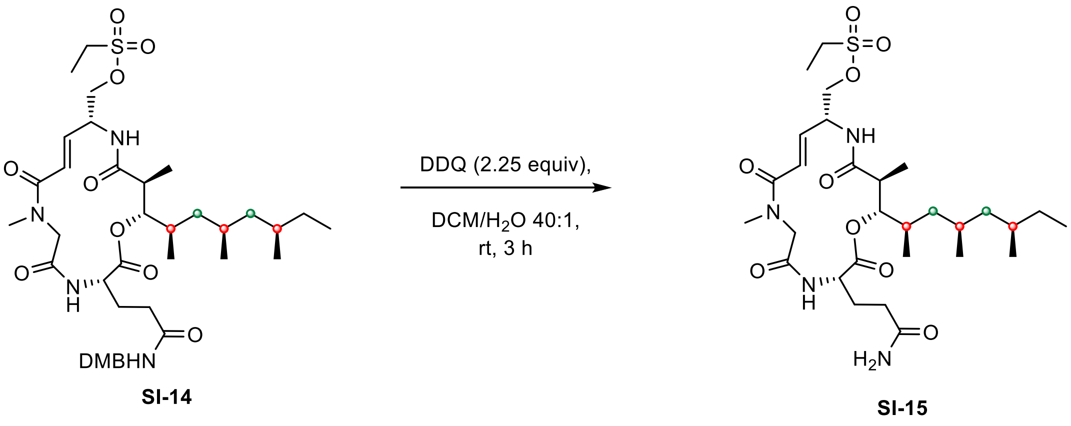

In a 20 mL round bottom flask, crude **SI-14** (containing a maximum of 8.9 mg, 11.4 µmol, 1.0 equiv) was dissolved in DCM (0.8 mL) and water (40 µL). To the colorless emulsion, DDQ (3.9 mg, 17.1 µmol, 1.5 equiv) dissolved in DCM (0.8 mL, pale-yellow solution) was added. The resulting pale-yellow solution was stirred at rt. After 2 h, additional DDQ (1.9 mg, 8.5 µmol, 1.5 equiv) dissolved in DCM (0.8 mL) was added. After a total of 3

h, dry toluene (10 mL) was added, and the resulting mixture was concentrated under reduced pressure to give crude **SI-15** (25.0 mg) as a brown solid. The crude mixture was used directly for step 2.4.9.

2.4.9. 3-((3*S*,14*S*,15*S*,*E*)-15-((2*R*,4*R*,6*R*)-4,6-dimethyloctan-2-yl)-7,14-dimethyl-11-methylene-2,5,8,13-tetraoxo-1-oxa-4,7,12-triazacyclopentadec-9-en-3-yl)propanamide (3)

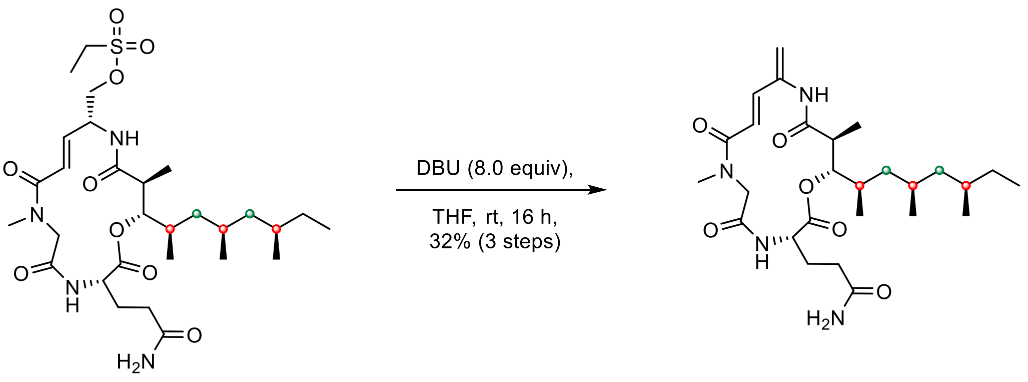

SI-15 **(–)-Rakicidin F (3)**

In a 25 mL round bottom flask, crude **SI-15** (containing a maximum of 7.2 mg, 11.4 µmol, 1.0 equiv) was dissolved in THF (3 mL) (rose solution). DBU (13.6 µL, 13.9 µg, 91.3 µmol, 8.0 equiv) was added at rt, resulting in a color change from rose to dark brown. After 16 h of stirring, sat. aq. $NaHCO_3$ (40 mL) and EtOAc (40 mL) were added to the reaction mixture and the layers were separated. The aq. layer was extracted with EtOAc (4x 20 mL). The org. layers were washed with $NaHCO_3$ (40 mL), brine (40 mL), combined, dried over $MgSO_4$, filtered, and concentrated under reduced pressure to give 11.8 mg of a pale-yellow oil (^{1}H-NMR with ISTD (benzene): 41% over 3 steps). The crude residue was purified by a pipette column chromatography (SiO_2, EtOAc:MeOH 95:5 to 5:1). The product-containing fractions were combined and concentrated partially (about 5 mL solvent remained). *t*-BuOH (10 mL) was added, and the resulting solution was concentrated again partially (about 5 mL solvent remained). The residue was frozen and lyophilized to afford (–)-rakicidin F (**3**) (1.9 mg, 3.65 µmol, 32% over 3 steps) as an off-white foam.

TLC (SiO_2; DCM:MeOH 10:1, CAM, UV): R_f = 0.16. **$[\alpha]_D^{18}$** = -78.80 (c = 0.20 in MeOH). **^{1}H NMR** (500 MHz, CD_3OD) δ 7.12 (d, *J* = 14.9 Hz, 1H), 6.19 (d, *J* = 14.9 Hz, 1H), 5.54 (s, 1H), 5.47 (s, 1H), 5.38 (dd, *J* = 10.3, 1.8 Hz, 1H), 4.59 (dd, *J* = 10.3, 4.7 Hz, 1H), 4.46 (d, *J* = 17.8 Hz, 1H), 3.99 (d, *J* = 17.8 Hz, 1H), 3.13 (s, 3H), 2.91 (dq, *J* = 10.4, 7.0 Hz, 1H), 2.34 – 2.25 (m, 2H), 2.17 – 2.07 (m, 1H), 2.06 – 1.88 (m, 2H), 1.78 (dt, *J* = 14.0, 6.9 Hz, 1H), 1.48 – 1.35 (m, 2H), 1.28 (ddd, *J* = 14.0, 8.6, 5.7 Hz, 1H), 1.22 (d, *J* = 7.0 Hz, 3H), 1.21 – 1.14 (m, 1H), 1.12 – 0.99 (m, 1H), 1.04 (d, *J* = 6.8 Hz, 3H), 0.96 – 0.81 (m, 11H). **^{13}C NMR** (126 MHz, CD_3OD) δ 177.2, 175.7, 170.9, 170.2, 168.8, 141.3, 139.3, 120.3, 118.9, 78.0, 54.0, 53.8, 46.5, 44.2, 43.2, 37.6, 33.0, 32.8, 32.1, 30.9, 29.8, 28.2, 20.61, 20.56, 15.8, 14.2, 11.5. **IR** (thin film): υ = 3370, 2958, 2921, 2851, 1727, 1661, 1614, 1541, 1461, 1260, 1095, 1034 cm^{-1}. **HRMS** (ESI): calc. for $C_{27}H_{44}N_4O_6Na$ $[M+Na]^+$: 543.3153 m/z; found: 543.3153 m/z. (see NMR spectra)

2.5. Alternative Route

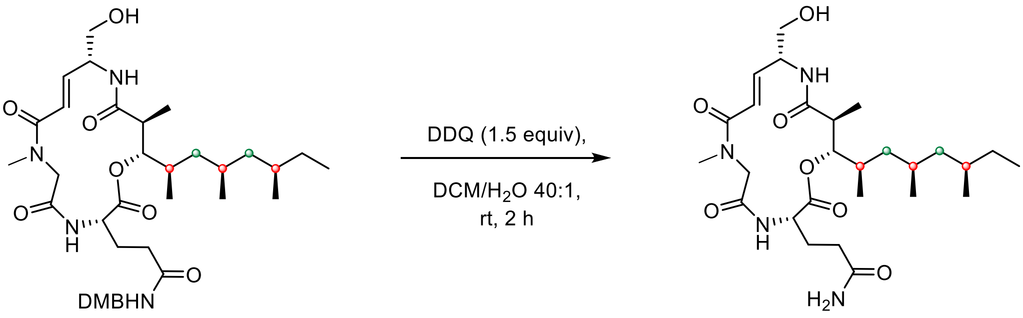

36 **SI-16**

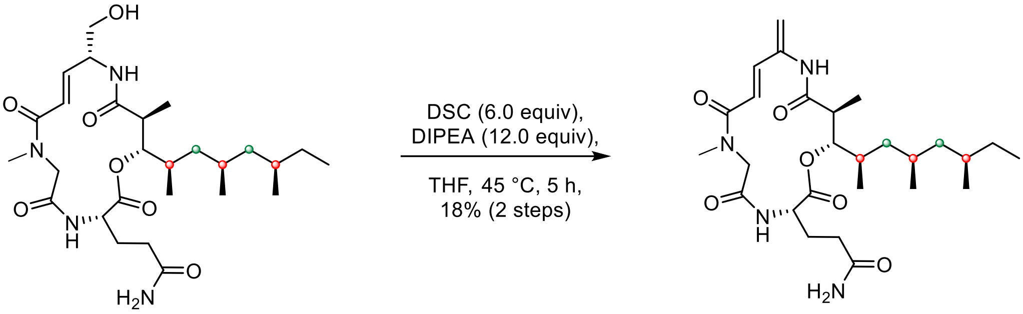

SI-16 **(–)-Rakicidin F (3)**

2.5.1. 3-((3*S*,11*R*,14*S*,15*S*,*E*)-15-((2*R*,4*R*,6*R*)-4,6-dimethyloctan-2-yl)-11-(hydroxymethyl)-7,14-dimethyl-2,5,8,13-tetraoxo-1-oxa-4,7,12-triazacyclopentadec-9-en-3-yl)propanamide (SI-16)

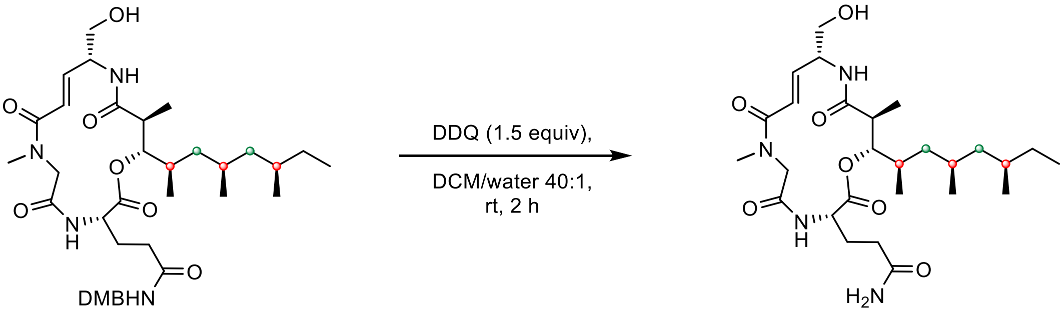

36 **SI-16**

In a 10 mL round bottom flask, primary alcohol **36** (8.3 mg, 12.0 µmol, 1.0 equiv) was dissolved in DCM (1 mL) and water (50 µL). To the white suspension, DDQ (4.1 mg, 18 µmol, 1.5 equiv) dissolved in DCM (1 mL, pale-yellow solution) was added. The resulting red suspension was stirred at rt for 2 h. The resulting dark-red suspension was then concentrated under reduced pressure to obtain crude **SI-16** (17.6 mg) as a brown solid. The crude mixture was used directly for step 2.5.2.

2.5.2. 3-((3*S*,14*S*,15*S*,*E*)-15-((2*R*,4*R*,6*R*)-4,6-dimethyloctan-2-yl)-7,14-dimethyl-11-methylene-2,5,8,13-tetraoxo-1-oxa-4,7,12-triazacyclopentadec-9-en-3-yl)propanamide (3)

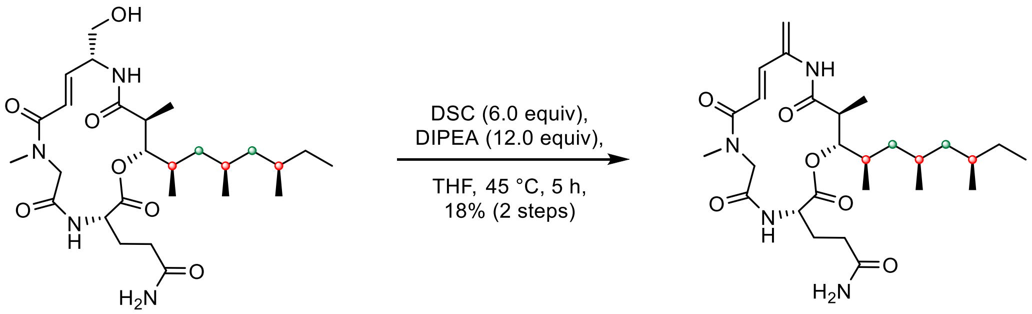

SI-16 **(–)-Rakicidin F (3)**

In a 10 mL round bottom flask, crude **SI-16** (half of the material of **SI-16** obtained in the previous step, containing a maximum of 3.3 mg, 6.0 μmol, 1.0 equiv) was dissolved in THF (1 mL). To the resulting red solution, DIPEA (13.0 μL, 9.4 mg, 72.4 μmol, 12 equiv), followed by N,N'-disuccinimidyl carbonate (DSC) (9.3 mg, 36.2 μmol, 6.0 equiv) was added. The resulting red solution was stirred at 45 °C (oil bath) for 5 h, before sat. aq. $NaHCO_3$ (20 mL) and EtOAc (20 mL) were added, and the layers were separated. The aq. layer was extracted with EtOAc (2x 20 mL). The org. layers were washed with $NaHCO_3$ (20 mL), brine (20 mL), combined, dried over $MgSO_4$, filtered, and concentrated under reduced pressure to give 3.5 mg of a pale-yellow greenish oil. The crude residue was purified by a pipette column chromatography (SiO_2, 100% EtOAc to EtOAc:MeOH 5:1) to afford **3** (small impurities, 560 μg, 1.08 μmol, 18% over 2 steps) as a pale-yellow oil.

Remark: After pipette column chromatography, the product-containing fractions were combined and fully concentrated. Unfortunately, upon concentration, partial decomposition occurred. To avoid this, *t*-BuOH is added to the combined fractions and the solvent is removed by lyophilization (see 2.4.9.)

2.6. Data Comparison of Synthesized (–)-Rakicidin F and Natural (+)-Rakicidin F[4]

2.6.1. ^{1}H NMR Data Comparison (CD_3OD)

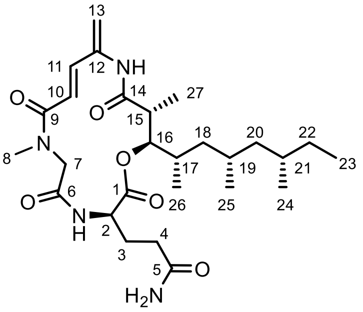

(+)-Rakicidin F (2)

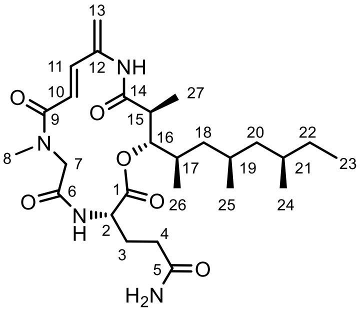

(–)-Rakicidin F (3)

position	**Natural (+)-Rakicidin F (2)** **δ_H mult (*J* in Hz)[4]** (600 MHz)	**(–)-Rakicidin F (3)** **δ_H mult (*J* in Hz)** (500 MHz)	**$\Delta\delta_H$/ppm**
**2, CH**	4.59, dd (10.2, 4.2)	4.59, dd (10.3, 4.7)	0.00
**3, CH_2**	2.12, m; 1.96, m	2.12, m; 1.96, m	0.00; 0.00
**4, CH_2**	2.29, t (7.8)	2.29, m	0.00
**7, CH_2**	4.47, d (17.4); 3.99, d (18.0)	4.46, d (17.8); 3.99, d (17.8)	-0.01; 0.00
**8, CH_3**	3.13, s	3.13, s	0.00
**10, CH**	6.20, d (15.0)	6.19, d (14.9)	-0.01
**11, CH**	7.12, d (14.4)	7.12, d (14.9)	0.00
**13, CH_2**	5.54, s; 5.46, s	5.54, s; 5.47, s	0.00; +0.01
**15, CH**	2.92, m	2.91, dq (10.4, 7.0)	-0.01
**16, CH**	5.38, dd (10.2, 1.2)	5.38, dd (10.3, 1.8)	0.00
**17, CH**	2.02, m	2.02, m	0.00
**18, CH_2**	1.28, m; 0.92, m	1.29, m; 0.92, m	+0.01; 0.00
**19, CH**	1.79, m	1.78, m	-0.01
**20, CH_2**	1.18, m; 0.92, m	1.18, m; 0.92, m	0.00; 0.00
**21, CH**	1.42, m	1.42, m	0.00
**22, CH_2**	1.42, m; 1.07, m	1.42, m; 1.07, m	0.00; 0.00
**23, CH_3**	0.88, m	0.88, m	0.00
**24, CH_3**	0.88, m	0.88, m	0.00
**25, CH_3**	0.88, m	0.88, m	0.00
**26, CH_3**	1.04, d (6.6)	1.04, d (6.8)	0.00
**27, CH_3**	1.22, d (7.2)	1.22, d (7.0)	0.00

2.6.2. ^{13}C NMR Data Comparison (CD_3OD)

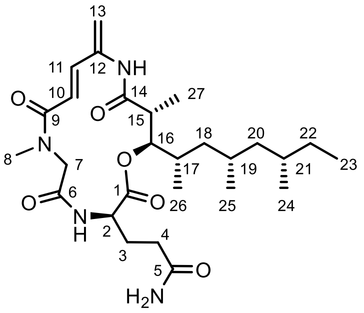

(+)-Rakicidin F (2)

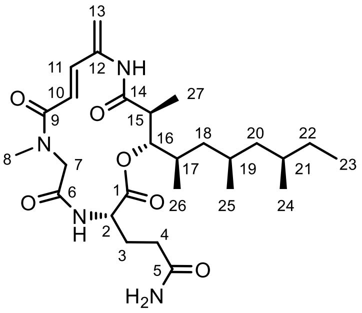

(–)-Rakicidin F (3)

position	Natural (+)-Rakicidin F (2)[4] δ_C (125 MHz)	(–)-Rakicidin F (3) δ_C (126 MHz)	$\Delta\delta_C$/ppm
**1, C**	171.1	170.9	-0.2
**2, CH**	53.9	53.8	-0.1
**3, CH$_2$**	31.0	30.9	-0.1
**4, CH$_2$**	33.1	33.0	-0.1
**5, C**	177.3	177.2	-0.1
**6, C**	170.3	170.2	-0.1
**7, CH$_2$**	54.1	54.0	-0.1
**8, CH$_3$**	37.8	37.6	-0.2
**9, C**	168.9	168.8	-0.1
**10, CH**	119.0	118.9	-0.1
**11, CH**	141.4	141.3	-0.1
**12, C**	139.5	139.3	-0.2
**13, CH$_2$**	120.5	120.3	-0.2
**14, C**	175.9	175.7	-0.2
**15, CH**	44.3	44.2	-0.1
**16, CH**	78.1	78.0	-0.1
**17, CH**	32.2	32.1	-0.1
**18, CH$_2$**	43.3	43.2	-0.1
**19, CH**	28.3	28.2	-0.1
**20, CH$_2$**	46.6	46.5	-0.1
**21, CH**	32.9	32.8	-0.1
**22, CH$_2$**	30.0	29.8	-0.2
**23, CH$_3$**	11.7	11.5	-0.2
**24, CH$_3$**	20.7	20.6	-0.1
**25, CH$_3$**	20.8	20.6	-0.2
**26, CH$_3$**	14.4	14.2	-0.2
**27, CH$_3$**	16.0	15.8	-0.2

2.6.3. HRMS, IR, $[\alpha]_D$ Data Comparison

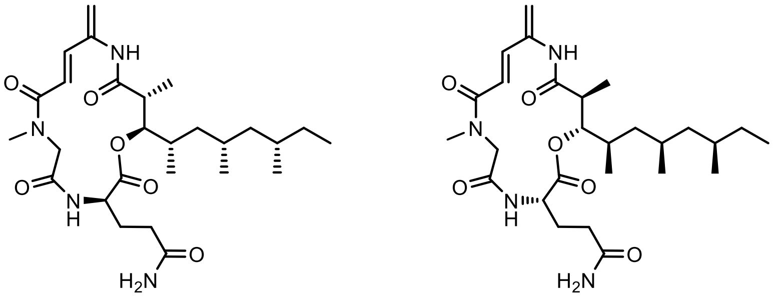

(+)-Rakicidin F (2) **(–)-Rakicidin F (3)**

	Natural (+)-Rakicidin F (2)[4]	(–)-Rakicidin F (3)
**HRMS (*m/z*)**	FAB	ESI
calculated	521.3334 for $C_{27}H_{45}N_4O_6$ [M+H]$^+$	543.3153 for $C_{27}H_{44}N_4O_6$ [M+Na]$^+$
found	521.3345	543.3153
**$[\alpha]_D$ (MeOH)**	+ 108 (c = 0.21)	- 79 (c = 0.20)
**IR (neat) cm^{-1}**	3372, 2962, 1655, 1616	3370, 2958, 1661, 1614

2.7. Screening of Conditions

2.7.1. Reduction Methyl ester 18

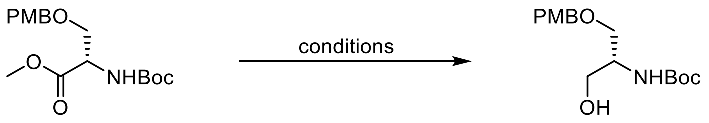

18 **SI-1**

Entry	Scale 18	Conditions	Yield /Observations
1	150 mg	$LiBH_4$ (1.2 equiv), THF, 0 °C to rt, 24 h	after 24 h: only traces of product, mainly starting material
2	150 mg	$NaBH_4$ (2.0 equiv), LiCl (2.0 equiv), MeOH, THF, -10 °C to rt, 24 h	after 24 h: only traces of product, mainly starting material
3	150 mg	$NaBH_4$ (2.0 equiv), $CaCl_2$ (2.0 equiv), EtOH, THF, 0 °C to rt, 2 h	quant. yield
4	2.0 g	$NaBH_4$ (2.0 equiv), $CaCl_2$ (2.0 equiv), EtOH, THF, 0 °C to rt, 2 h	98%

2.7.2. Horner-Wadsworth-Emmons Reaction

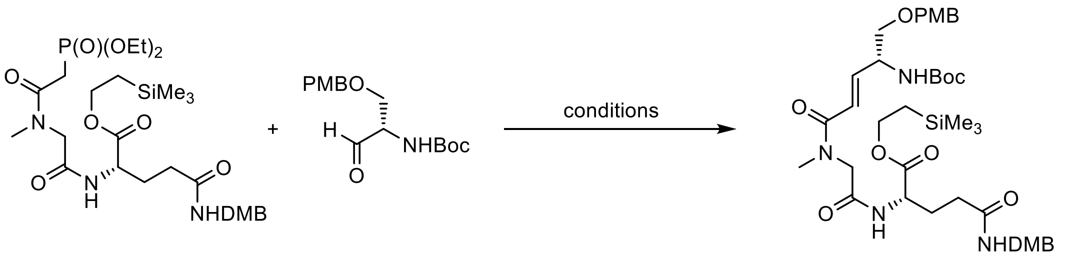

SI-17 **19** **SI-18**

Entry	Scale SI-17	Conditions	Yield /Observations
1	62 mg	**19** (1.1 equiv), $MgBr_2 \cdot Et_2O$ (1.2 equiv), NEt_3 (1.2 equiv), THF, rt, 18 h	42% yield (*E*:*Z* 1:0.04) 27% rec. **SI-17**
2	64 mg	**19** (1.1 equiv), $MgBr_2$ (1.2 equiv), NEt_3 (1.2 equiv), THF, rt, 18 h	50% yield (*E*:*Z* 1:0.05) 27% rec. **SI-17**
3[5]	64 mg	**19** (1.1 equiv), LiCl (2.0 equiv), DIPEA (1.1 equiv), MeCN, 0 °C to rt, 18 h	52% yield (*E*:*Z* 1:0.16) 42% rec. **SI-17**
4[5]	72 mg	**19** (2.0 equiv), LiCl (2.0 equiv), DIPEA (2.0 equiv), MeCN, 0 °C to rt, 18 h	62% yield (*E*:*Z* 1:0.16) 30% rec. **SI-17**
5	35 mg	**19** (1.5 equiv), NaH (1.0 equiv), THF, 0 °C to -78 °C to rt, 2.5 h	complex mixture; not pure after column chromatography; <77% isolated yield
6[6]	43 mg	**19** (1.2 equiv), $Ba(OH)_2 \cdot 8H_2O$ (0.8 equiv), THF/H_2O 80:1, rt, 20 min	full conversion, 76% yield (*E*:*Z* 1:0.08)

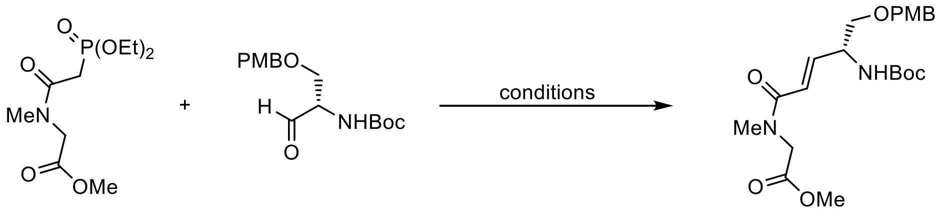

20 **19** **SI-3**

Entry	Scale 20	Conditions	Yield /Observations
1	184 mg	**19** (1.2 equiv), $MgBr_2 \cdot Et_2O$ (1.3 equiv), NEt_3 (1.3 equiv), THF, rt, 90 min	31% yield (*E*:*Z* 1:0.04)
2[6]	500 mg	**19** (1.2 equiv), $Ba(OH)_2 \cdot 8H_2O$ (0.8 equiv), THF/H_2O 80:1, 0 °C, 100 min	65% yield (*E*:*Z* 1:0.18)

2.7.3. Allyl Group Cleavage

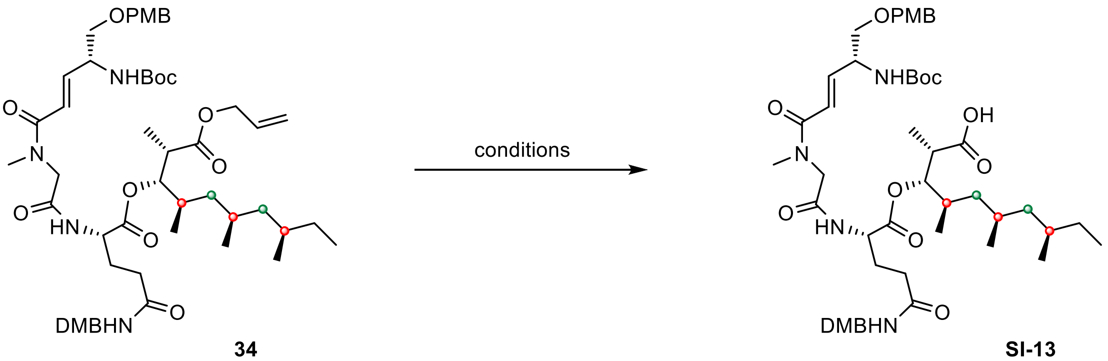

34 **SI-13**

Entry	Scale 34	Conditions	Yield /Observations
1	8.0 mg	$Pd(PPh_3)_4$ (45 mol%), *N*-Me-aniline (2.5 equiv), THF, rt, 23 h	incomplete conversion, 57%
2	21.6 mg	$Pd(PPh_3)_4$ (20 mol%), *N*-Me-aniline (4.0 equiv), THF, rt, 5 h	incomplete conversion, 62% (78% BRSM)
3	21.6 mg	$Pd(PPh_3)_4$ (1.0 equiv), *N*-Me-aniline (10 equiv), THF, rt, 30 min	full conversion, 56%
4	21.6 mg	$Pd(PPh_3)_4$ (20 mol%), $PhSiH_3$ (2.5 equiv), DCM, rt, 1 h	full conversion, 77%
5	66.5 mg	$Pd(PPh_3)_4$ (5 mol%), $PhSiH_3$ (2.5 equiv), DCM, rt, 1 h	full conversion, 75%

2.8. Comparison of the ^{13}C NMR Data of Rakicidin F with syn- and anti-configured Lipophilic Side Chains 30 and epi-30.

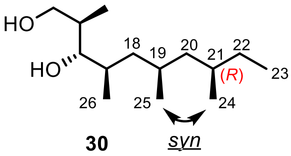

30

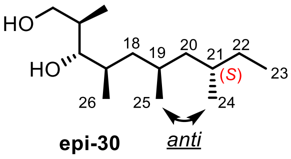

epi-30

position	Natural Rakicidin F[4] δC, type	30 (syn) δC, type	Δ	epi-30 (anti) δC, type	Δ
**18**	43.3, CH_2	43.2, CH_2	-0.1	43.9, CH_2	+0.6
**19**	28.3, CH	28.4, CH	+0.1	28.3, CH	+0.0
**20**	46.6, CH_2	46.2, CH_2	-0.4	45.6, CH_2	-1.0
**21**	32.9, CH	32.9, CH	+0.0	33.0, CH	+0.1
**22**	30.0, CH_2	30.0, CH_2	+0.0	31.8, CH_2	+1.8
**23**	11.7, CH_3	11.6, CH_3	-0.1	11.9, CH_3	+0.2
**24**	20.7, CH_3	20.5, CH_3	-0.2	19.4, CH_3	-1.3
**25**	20.8, CH_3	21.1, CH_3	+0.3	20.4, CH_3	-0.4

^1H NMR (500 MHz, CD_3OD) of mixture **30/epi-30**

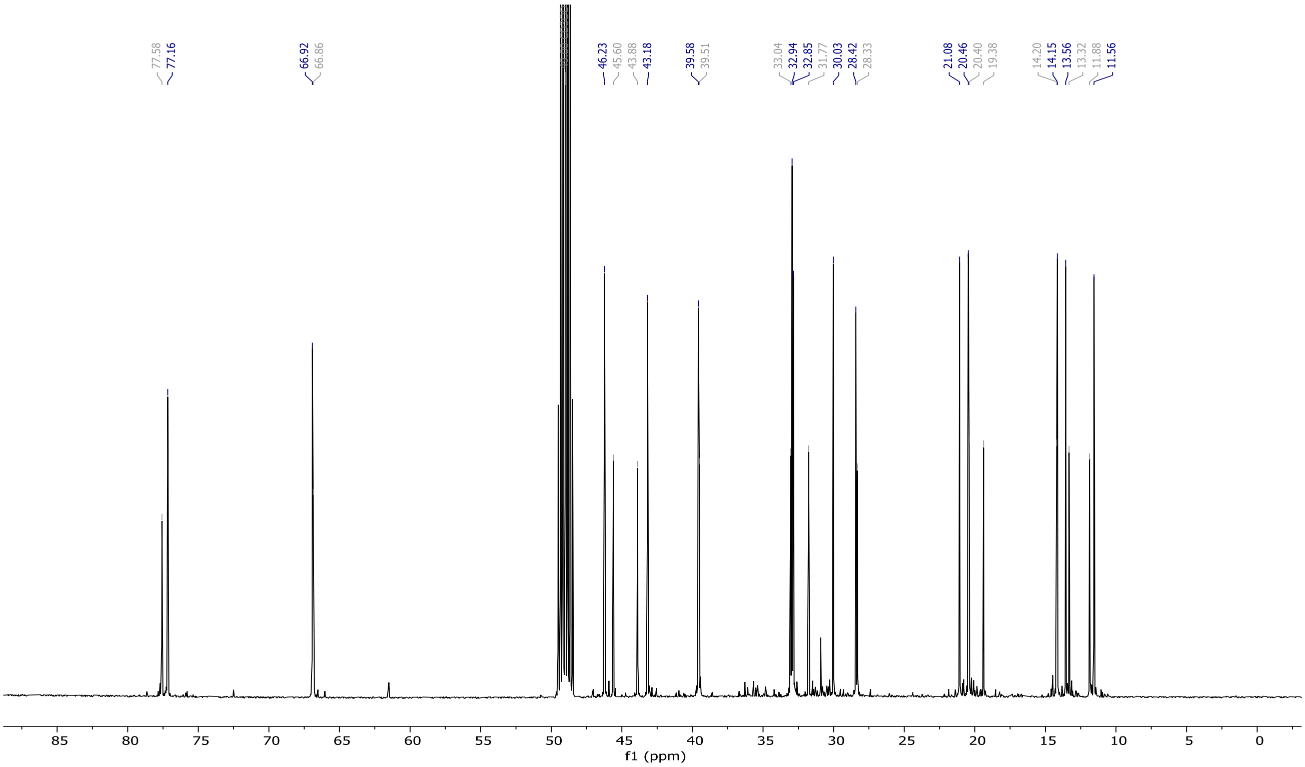

Blue numbers: major diastereomer (**30**); grey numbers: minor diastereomer (epi-**30**).

3. SPECTROSCOPIC DATA

^{1}H NMR (400 MHz, $CDCl_3$) of **18** (see procedure)

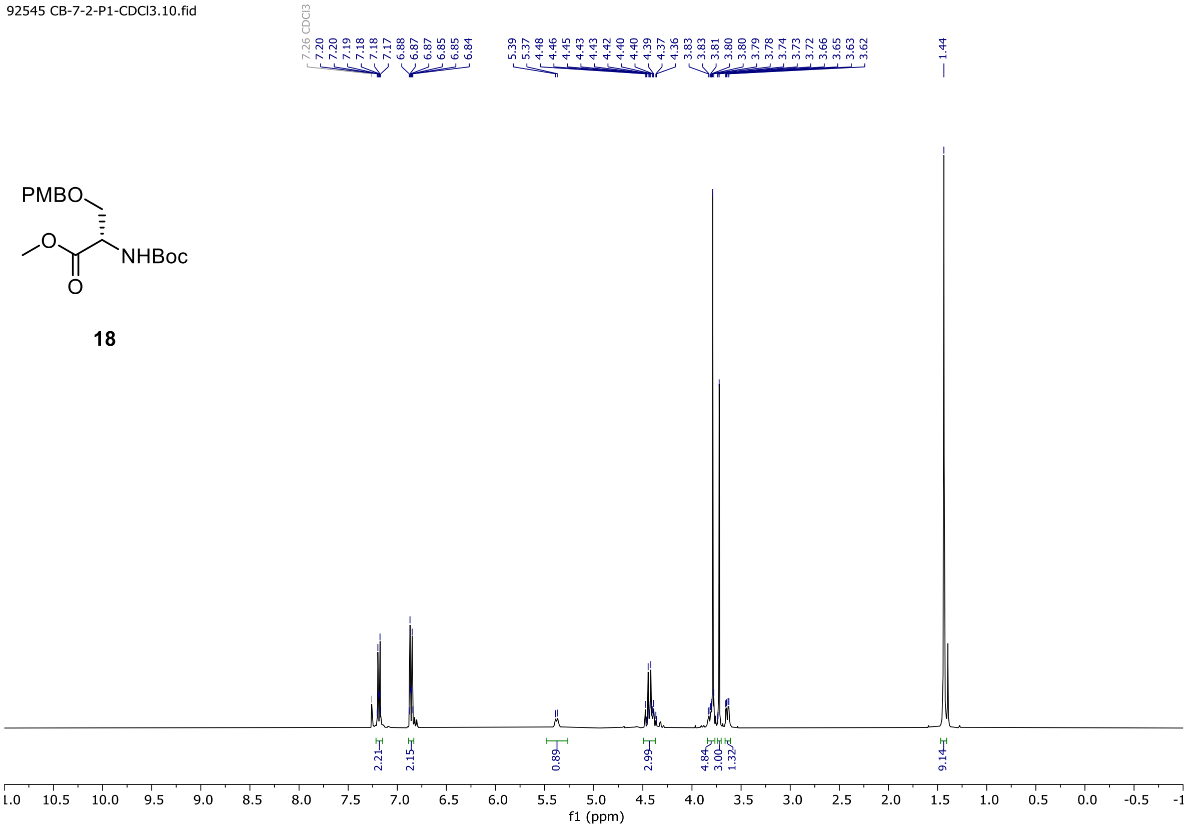

^{13}C NMR (101 MHz, $CDCl_3$) of **18**

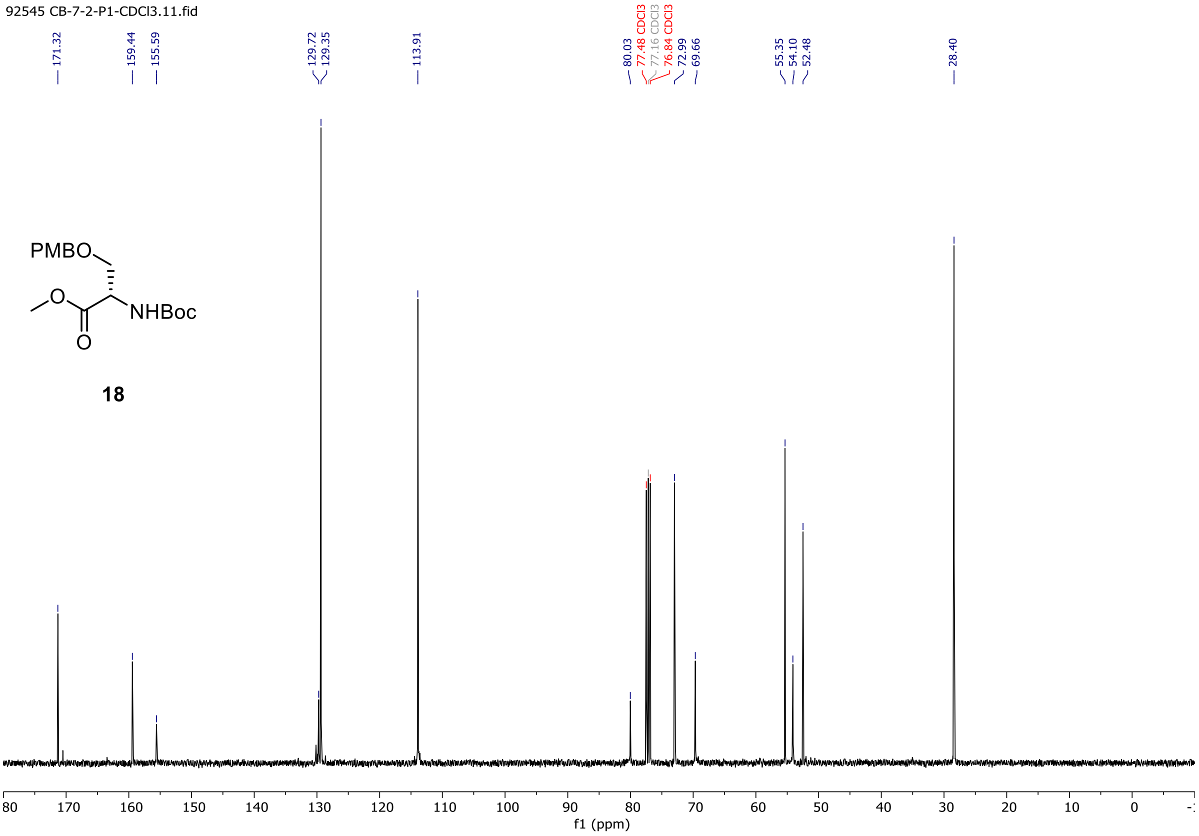

^{1}H NMR (500 MHz, $CDCl_3$) of **SI-1** (see procedure)

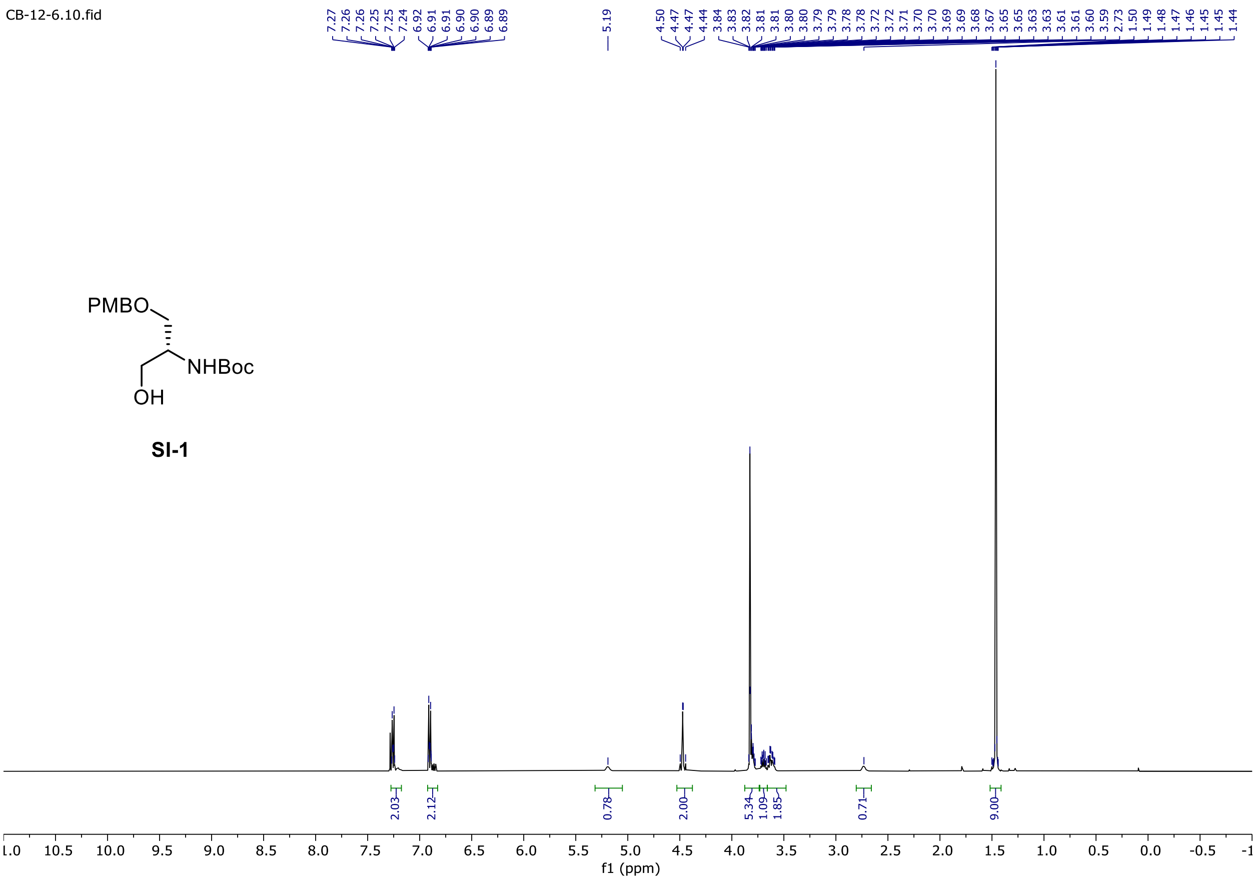

^{13}C NMR (126 MHz, $CDCl_3$) of **SI-1**

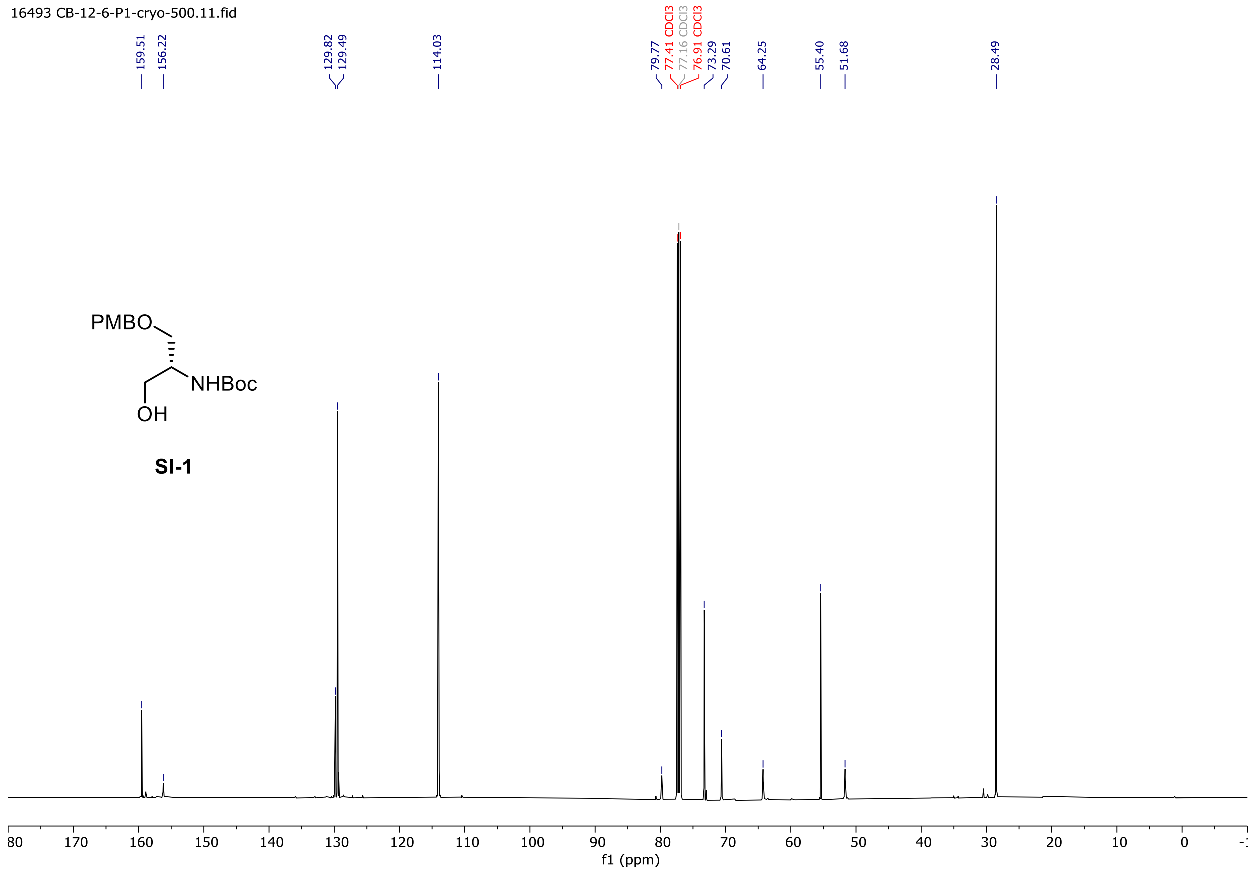

^{1}H NMR (400 MHz, $CDCl_3$) of **SI-2** (*see procedure*)

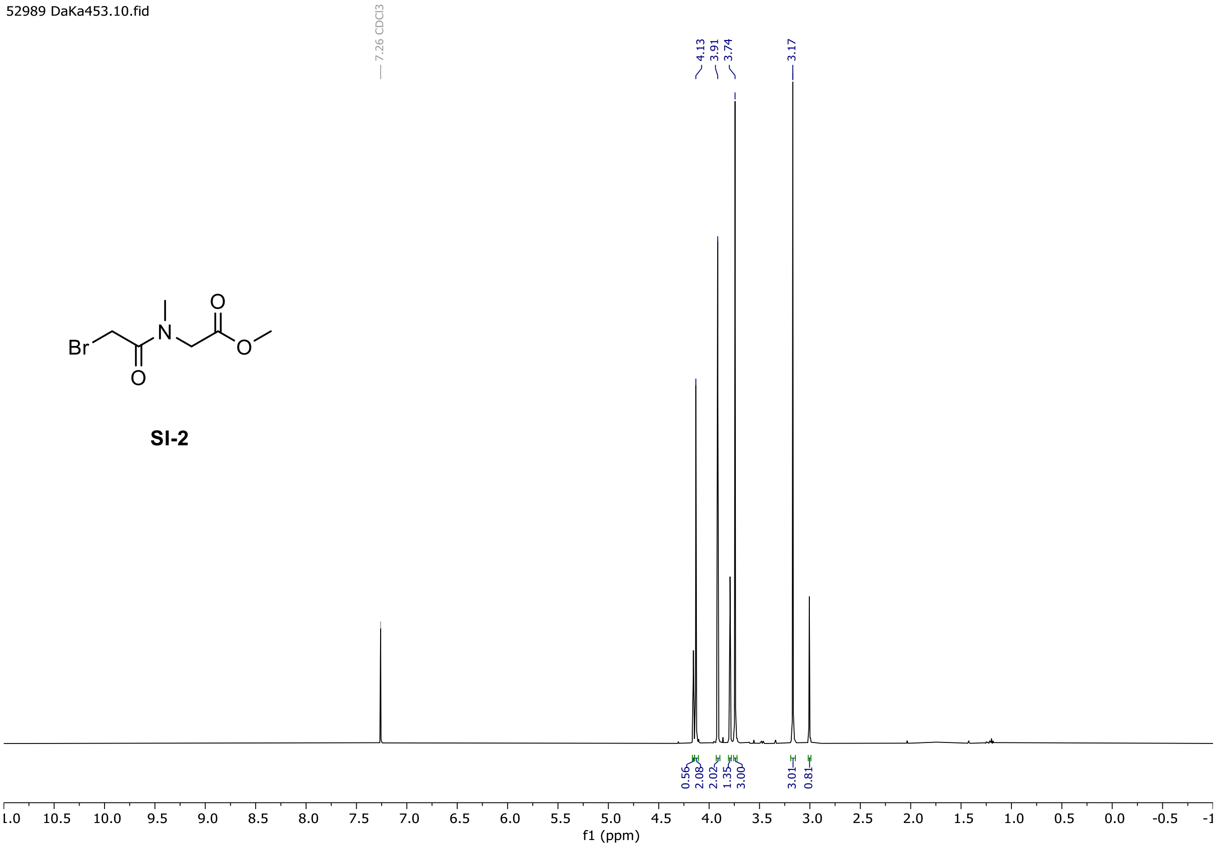

^{1}H NMR (400 MHz, $CDCl_3$) of **20** (*see procedure*)

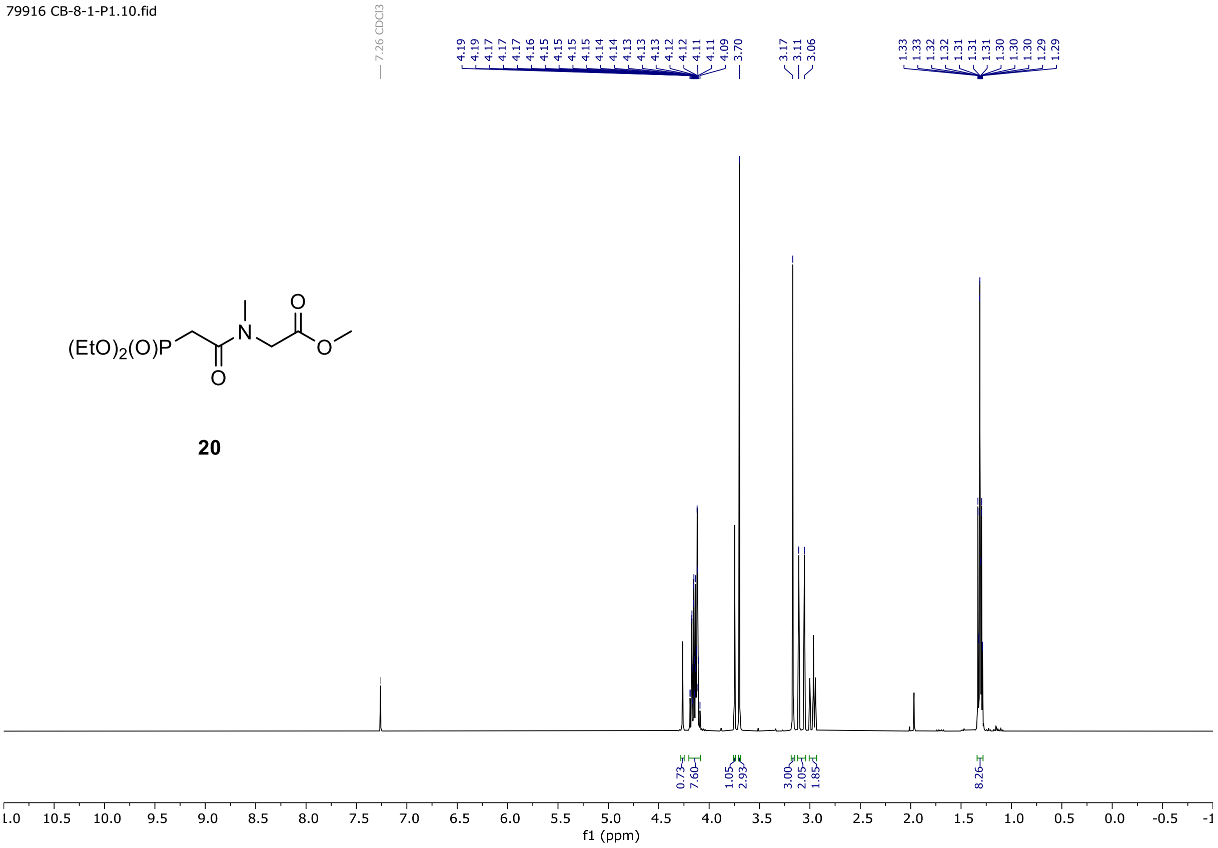

^{13}C NMR (101 MHz, $CDCl_3$) of **20**

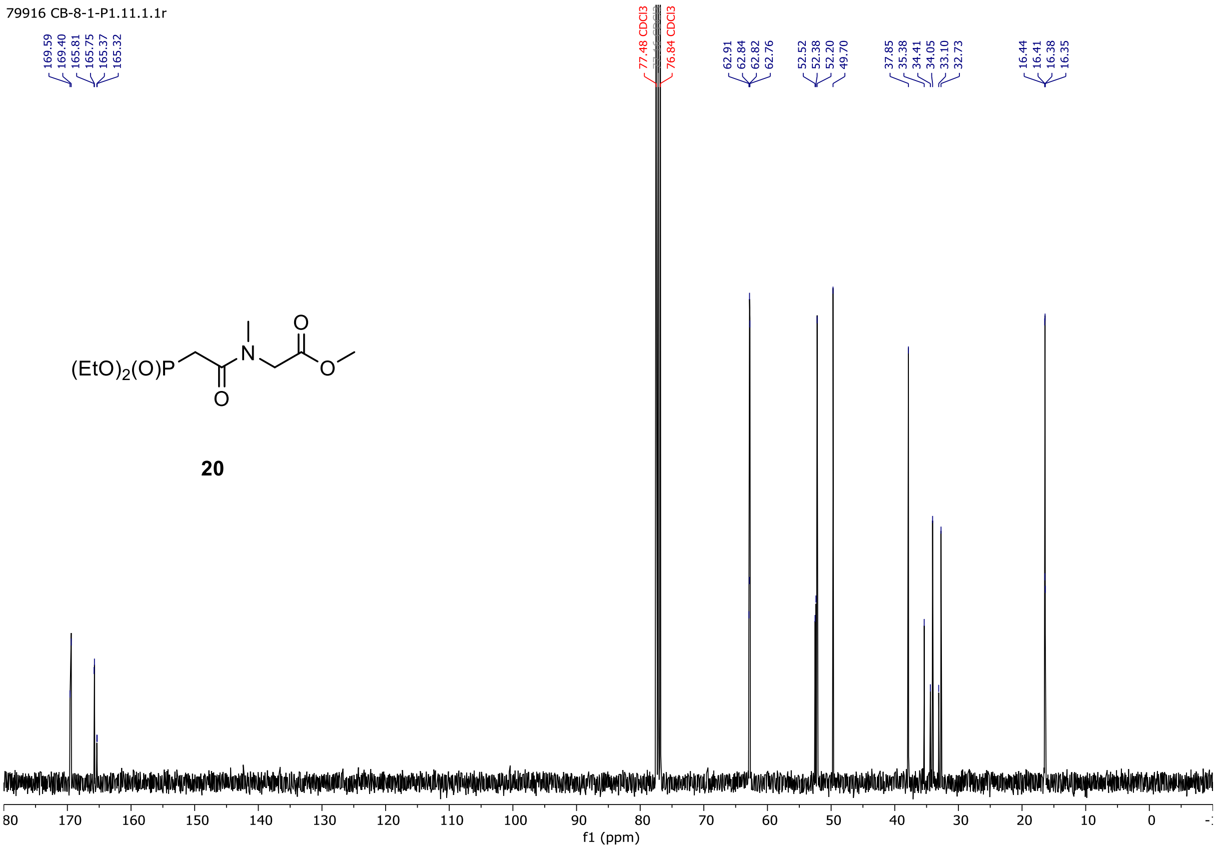

^{1}H NMR (400 MHz, CD_3OD) of **SI-3** (*see procedure*)

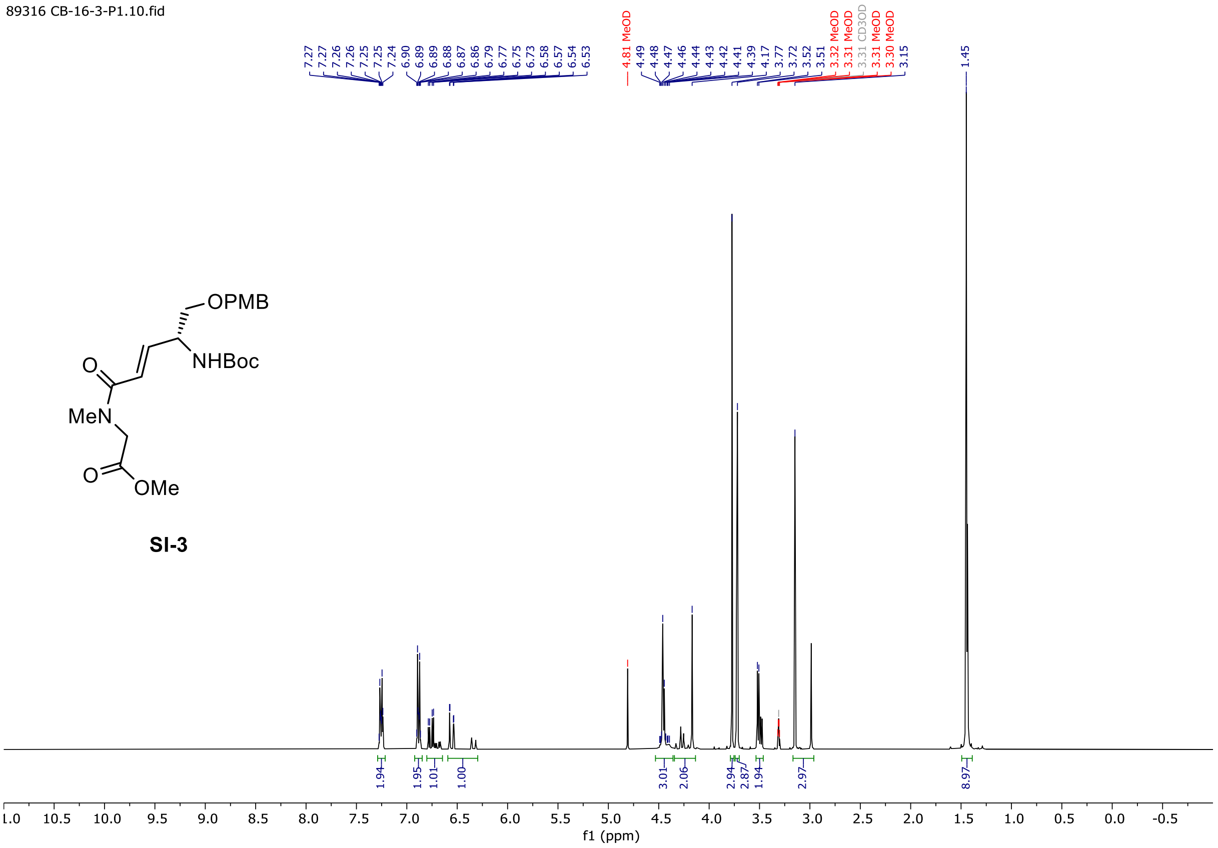

^{13}C NMR (101 MHz, CD_3OD) of **SI-3**

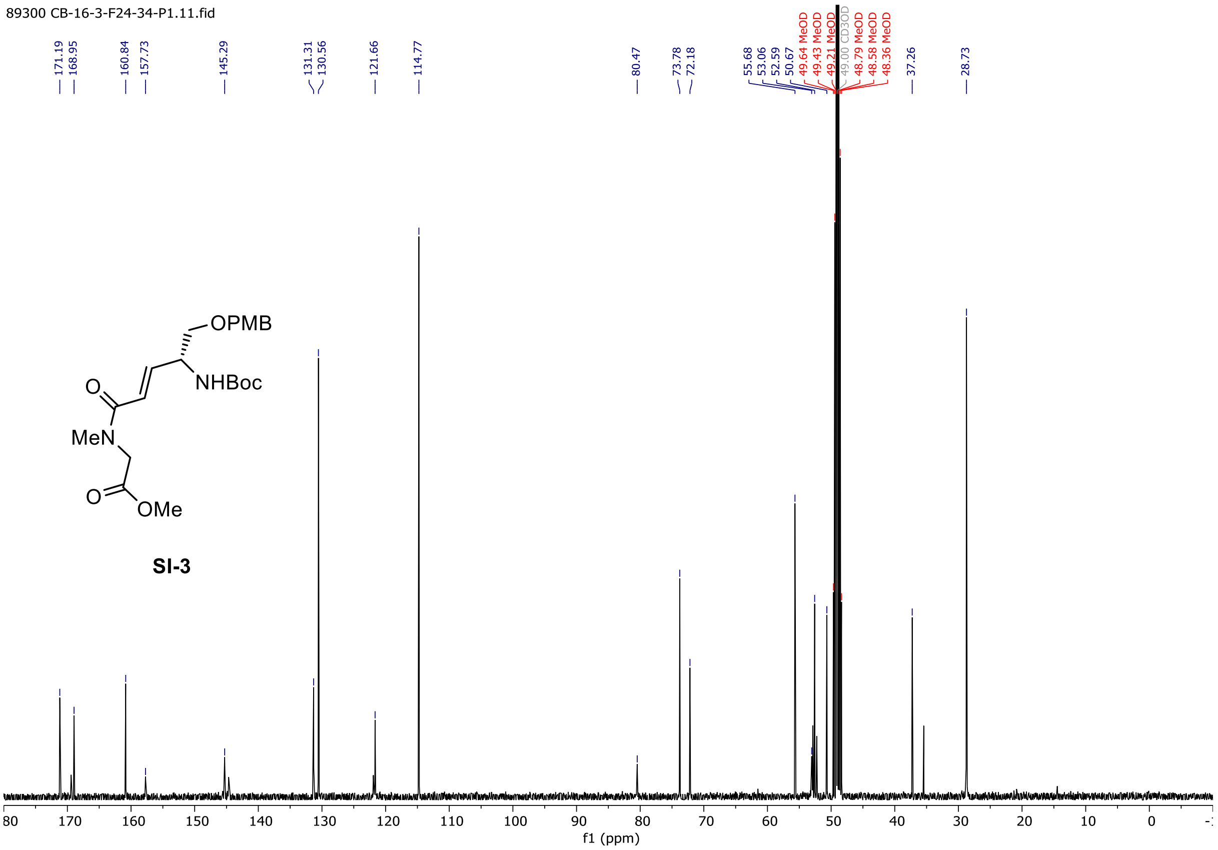

^{1}H NMR (400 MHz, $CDCl_3$) of **SI-4** (*see procedure*)

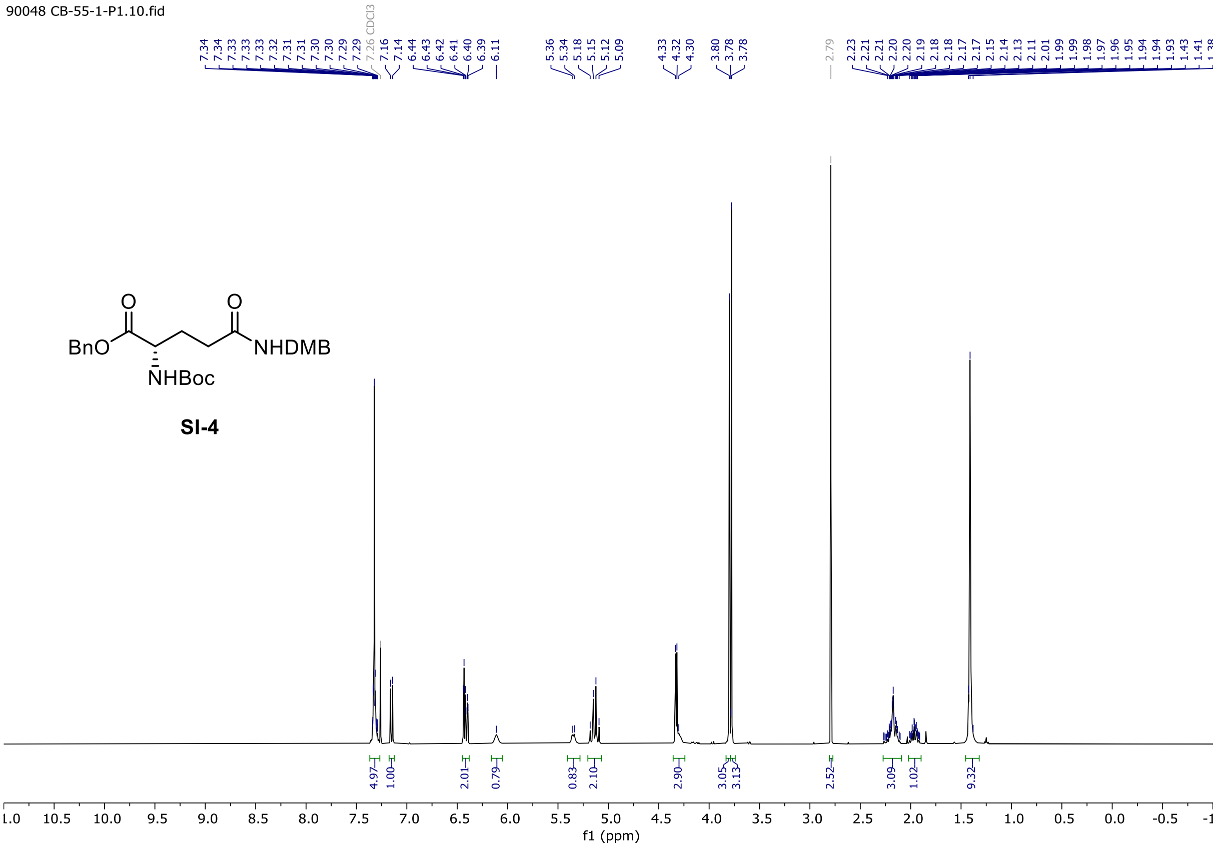

^{13}C NMR (101 MHz, $CDCl_3$) of **SI-4**

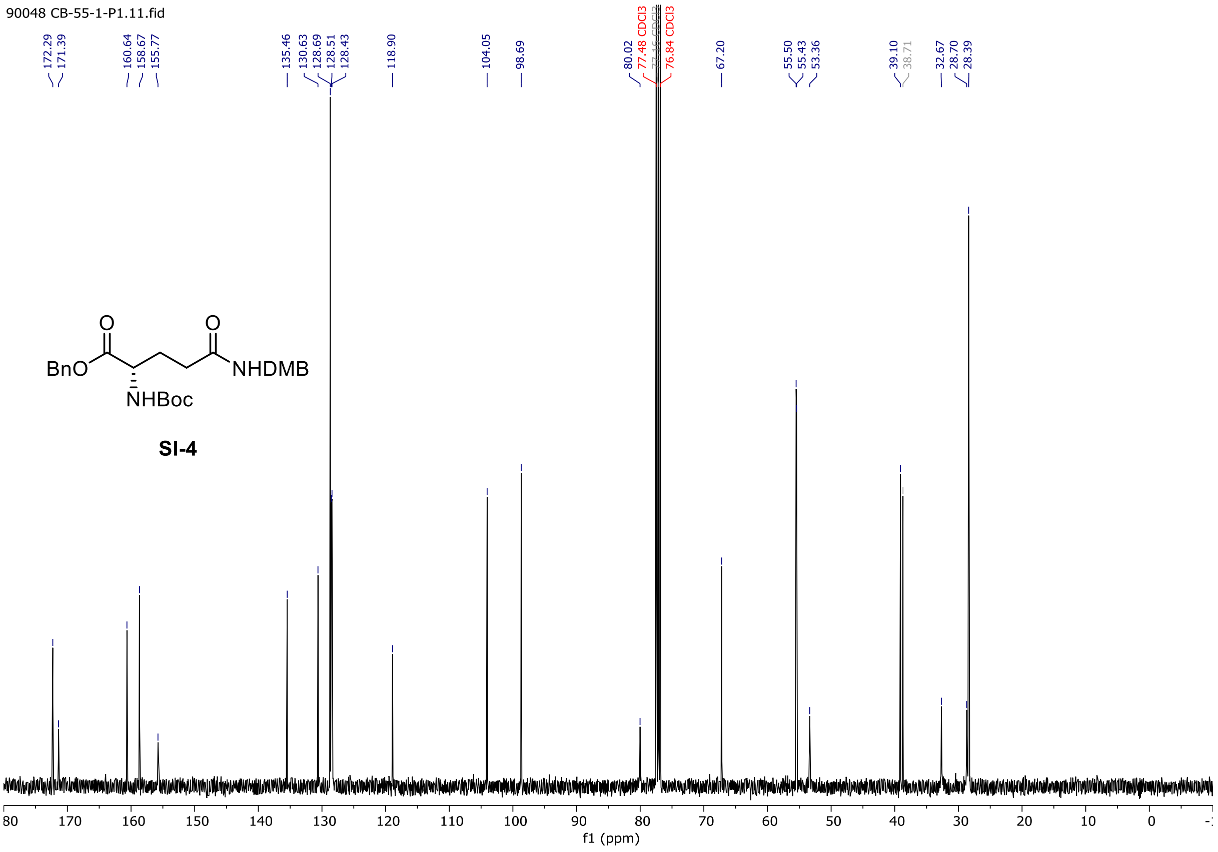

^{1}H NMR (400 MHz, $CDCl_3$) of **32** (see procedure)

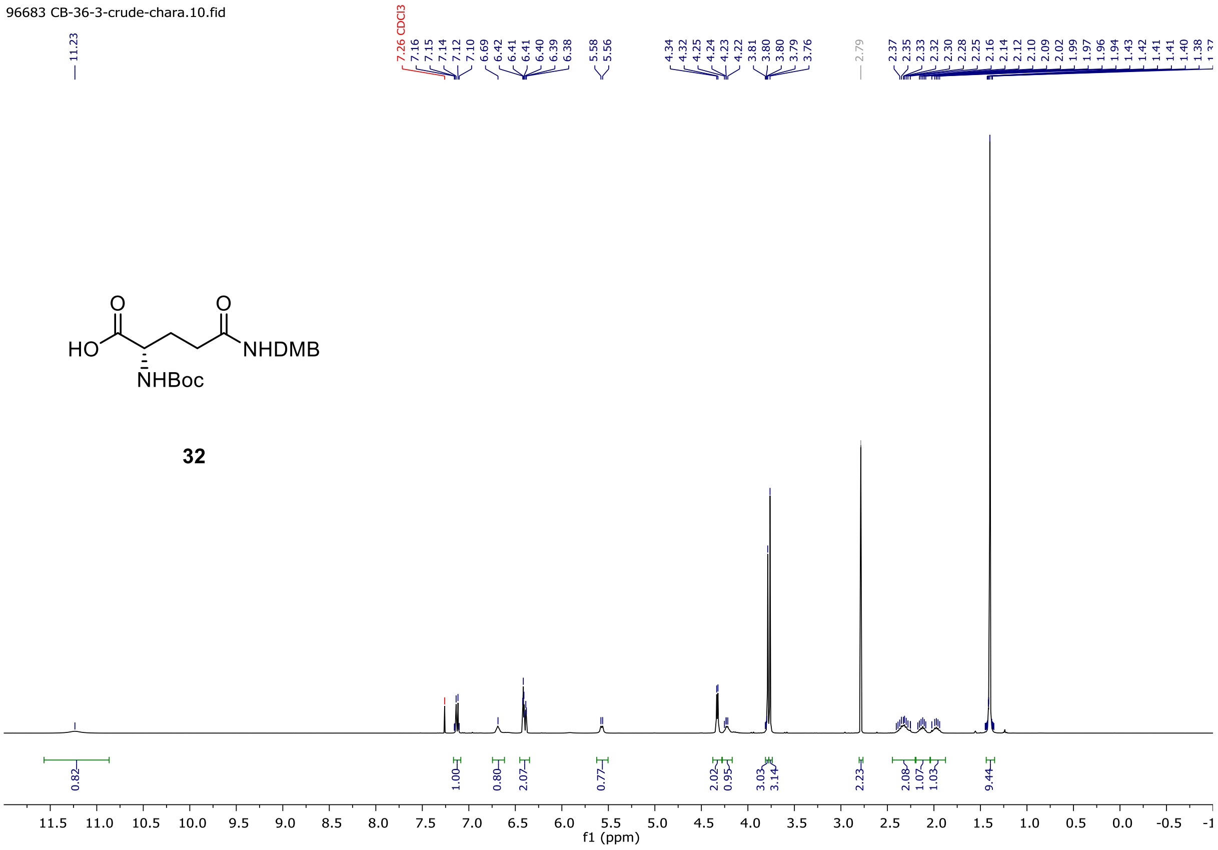

^{13}C NMR (101 MHz, $CDCl_3$) of **32**

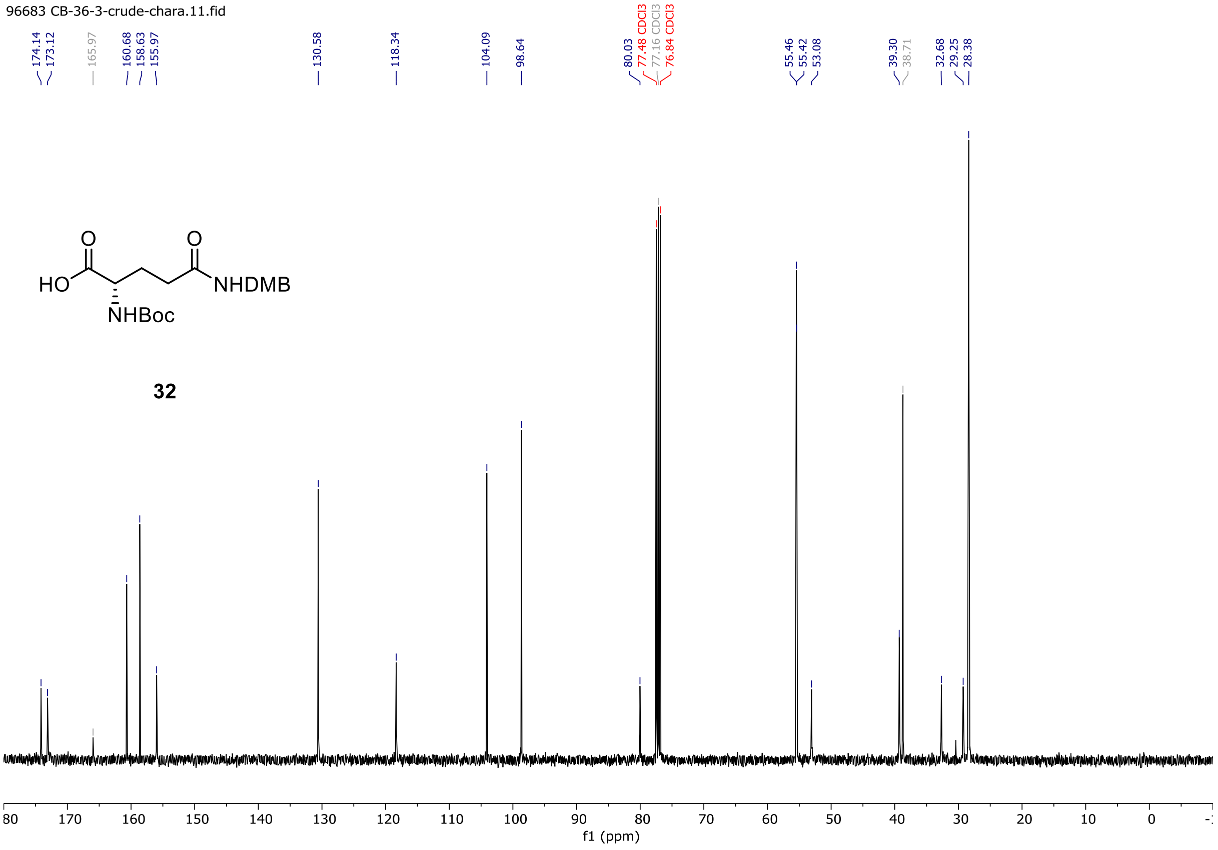

^{1}H NMR (500 MHz, $CDCl_3$) of **22** (*see procedure*)

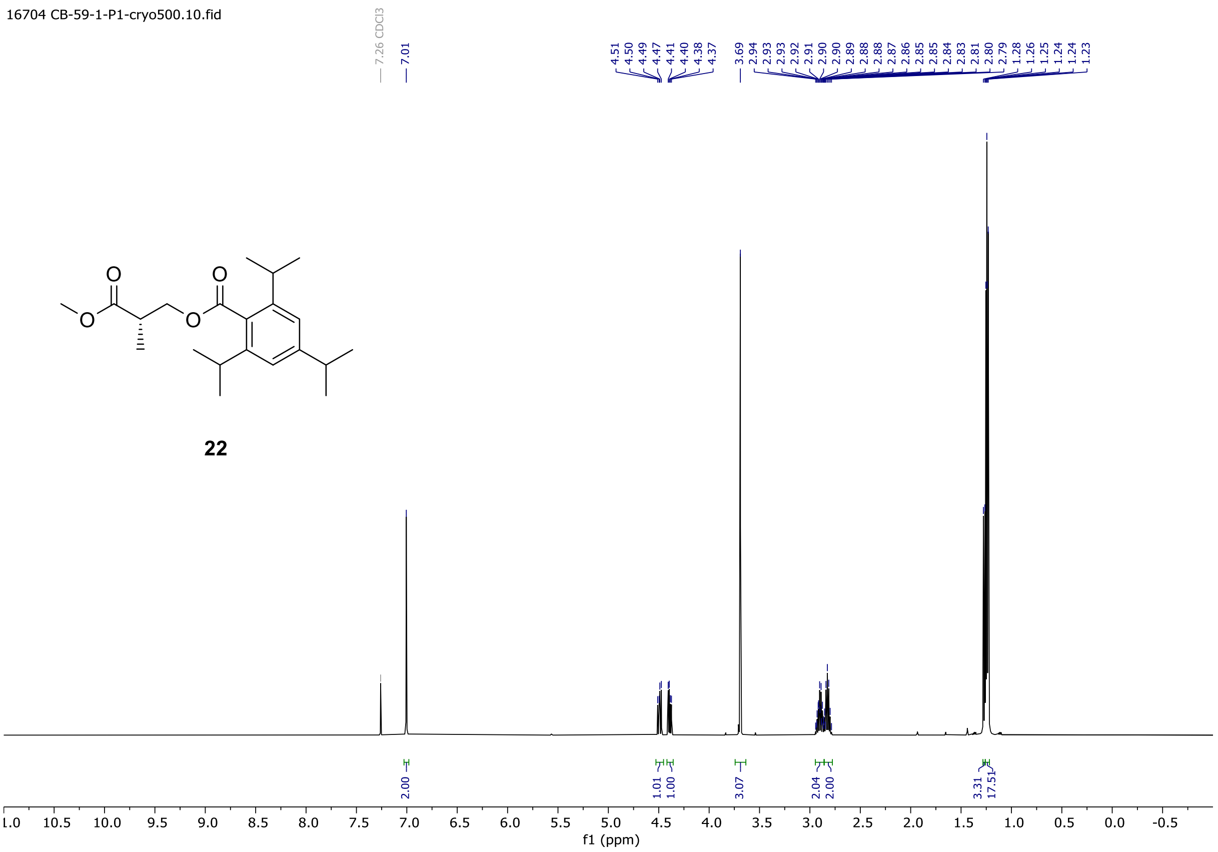

^{13}C NMR (101 MHz, $CDCl_3$) of **22**

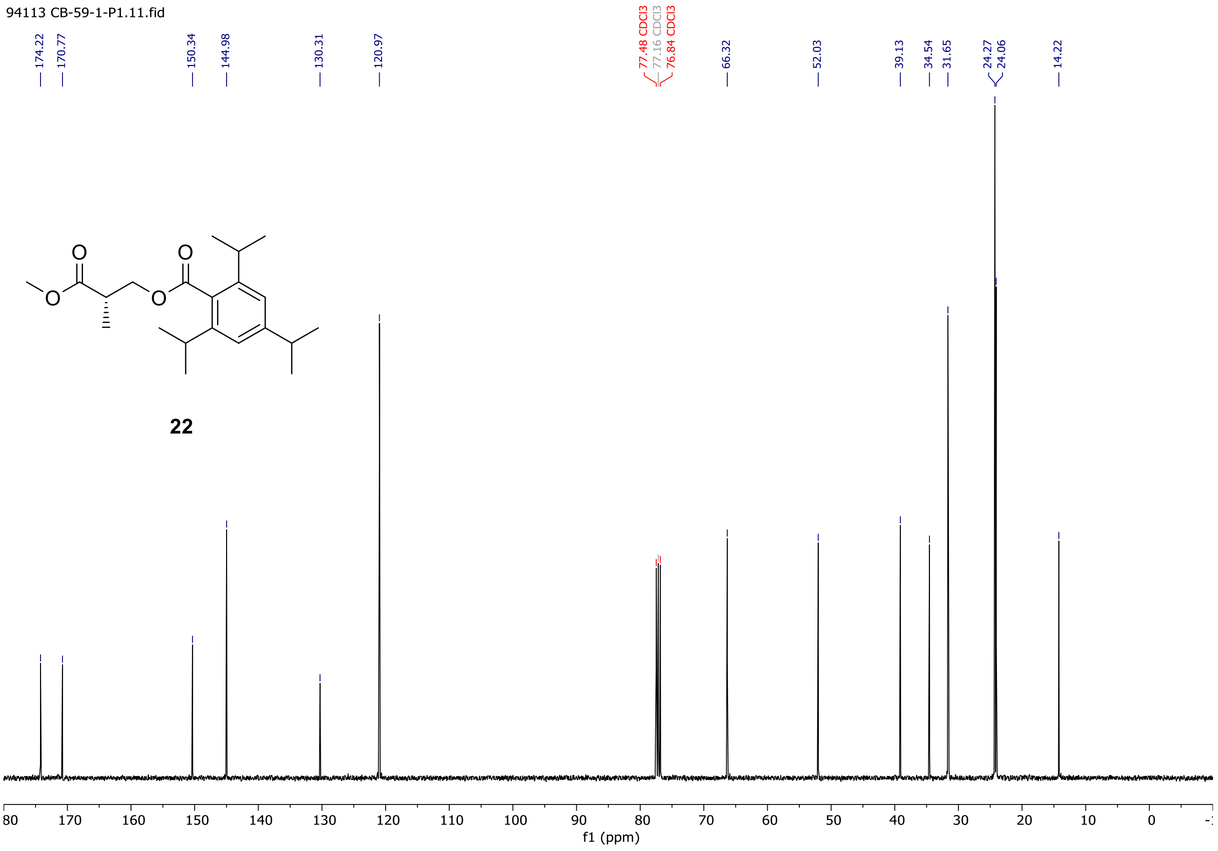

^{1}H NMR (400 MHz, $CDCl_3$) of **SI-5** (*see procedure*)

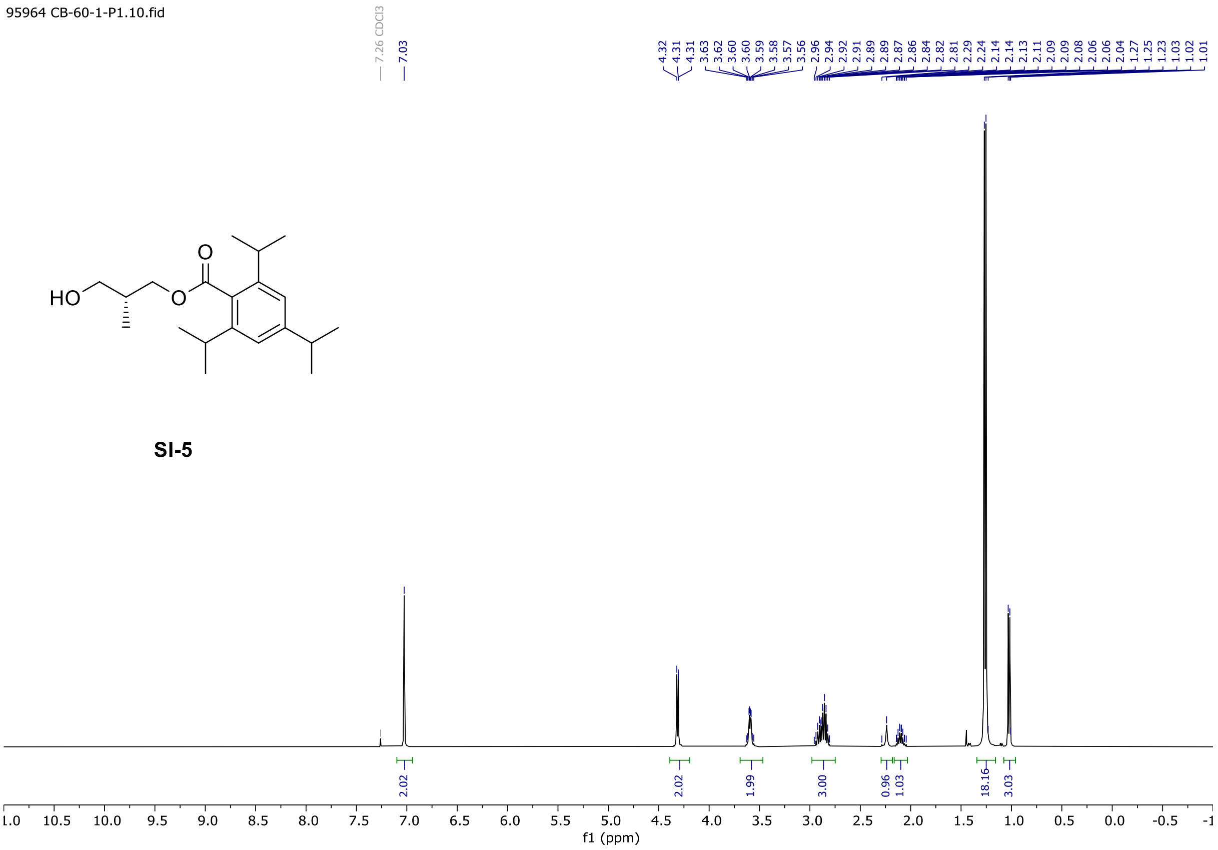

^{13}C NMR (101 MHz, $CDCl_3$) of **SI-5**

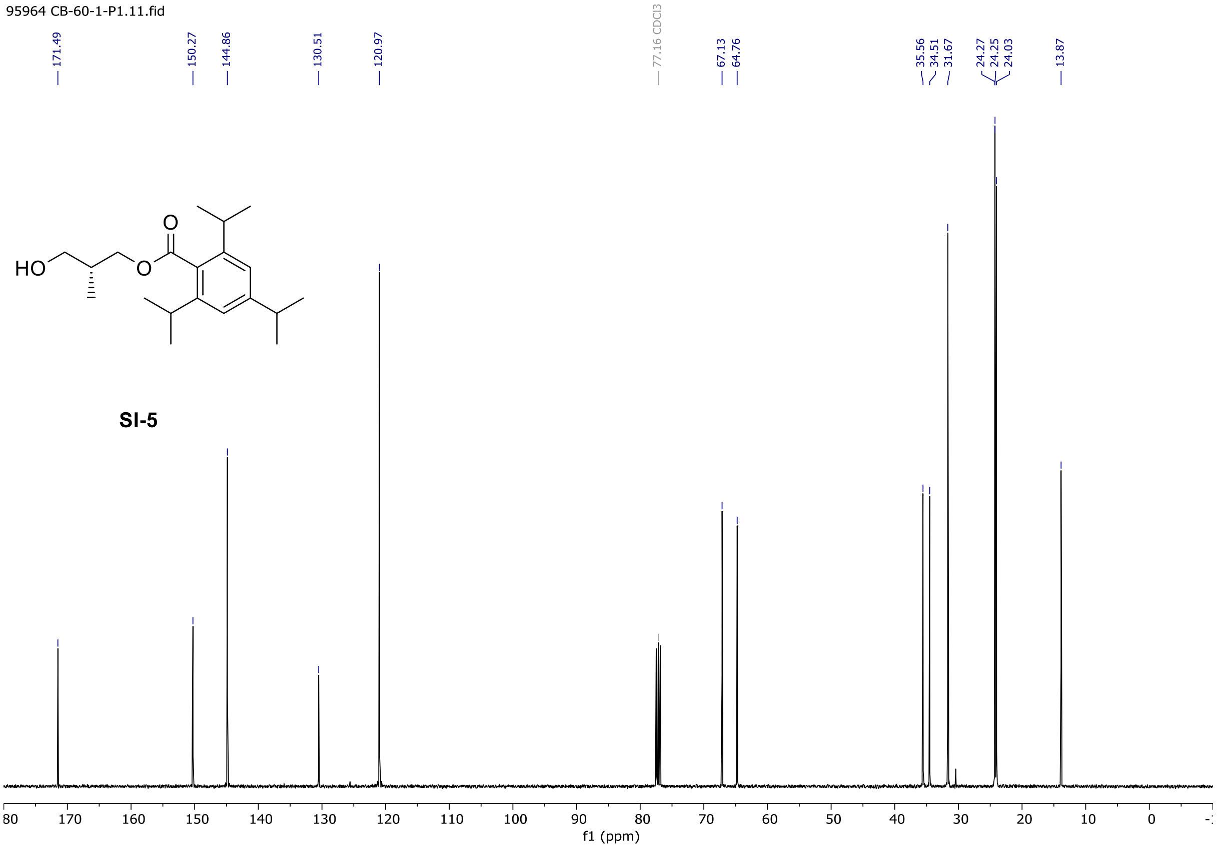

^{1}H NMR (400 MHz, $CDCl_3$) of **23** (*see procedure*)

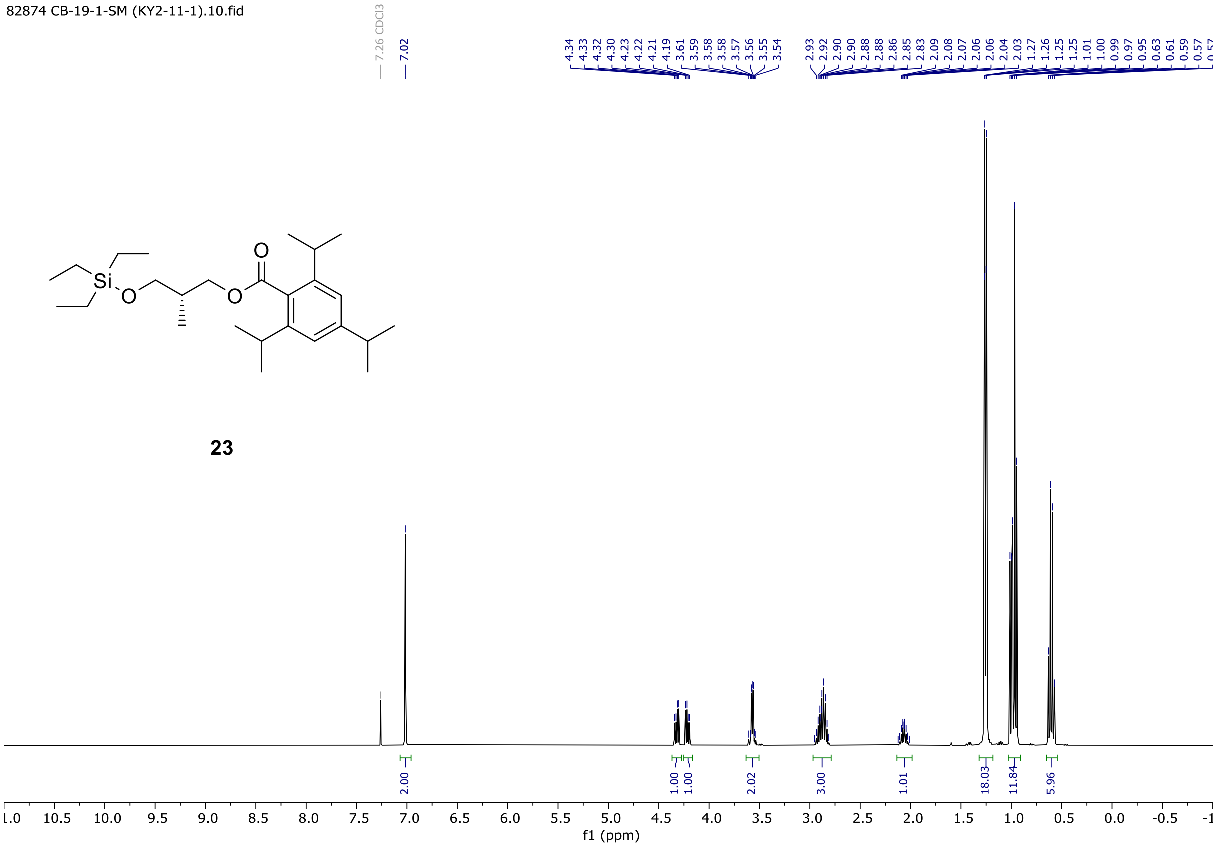

^{13}C NMR (101 MHz, $CDCl_3$) of **23**

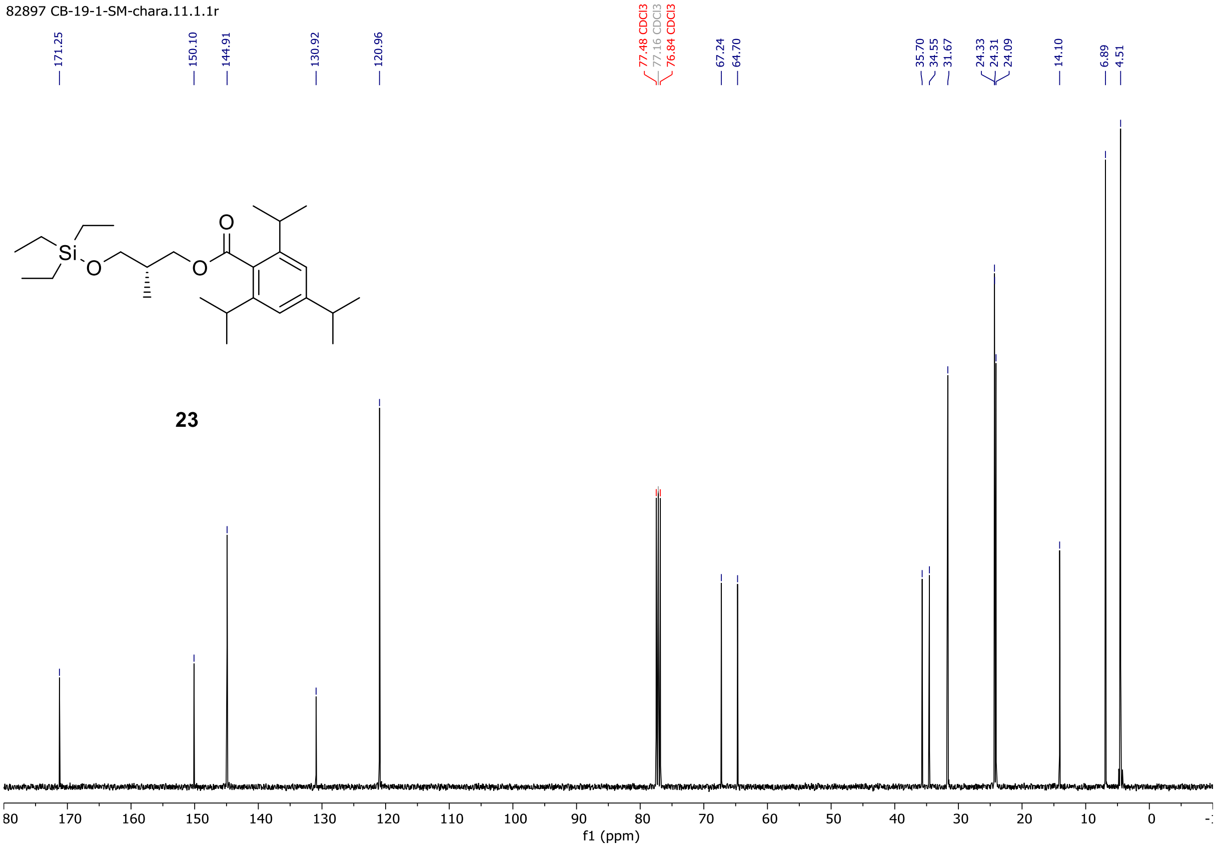

^{1}H NMR (400 MHz, $CDCl_3$) of **24** (*see procedure*)

82896 CB-19-1-P1.10.fid

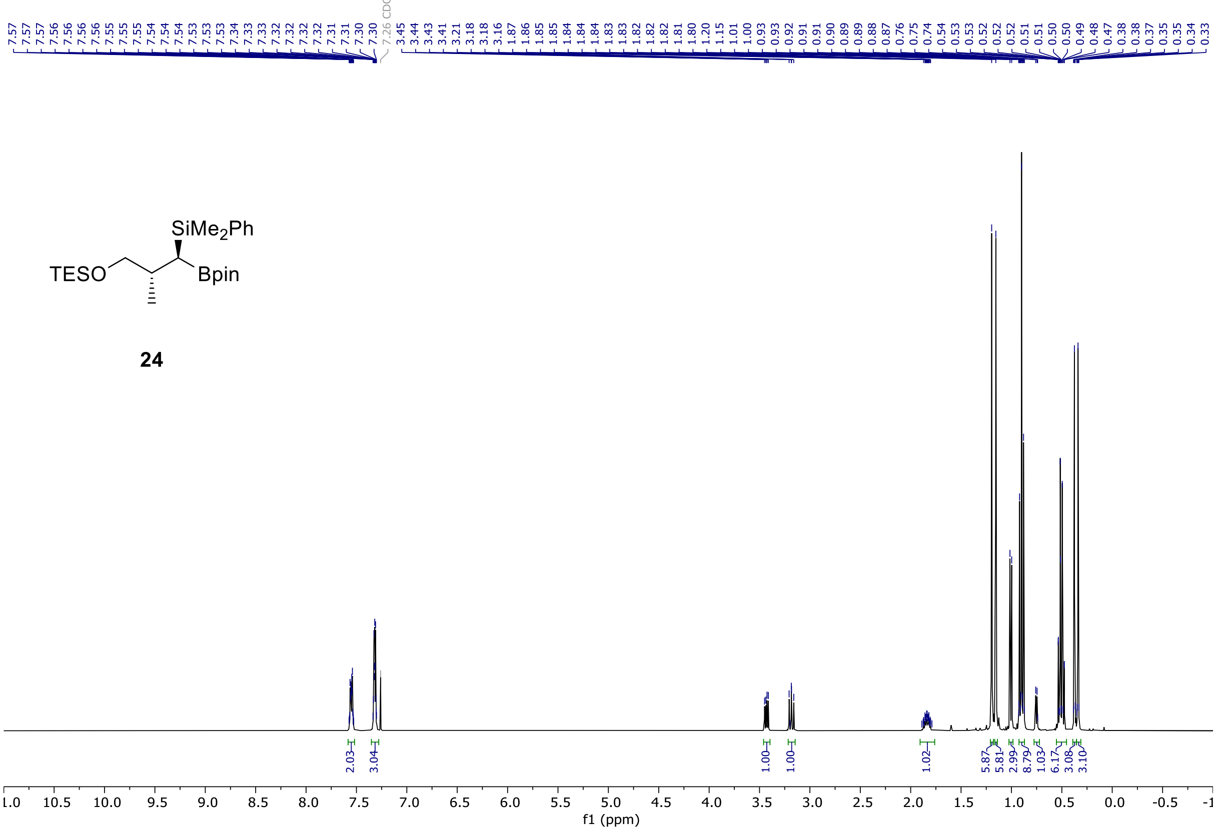

^{13}C NMR (101 MHz, $CDCl_3$) of **24**

82896 CB-19-1-P1.11.1.1r

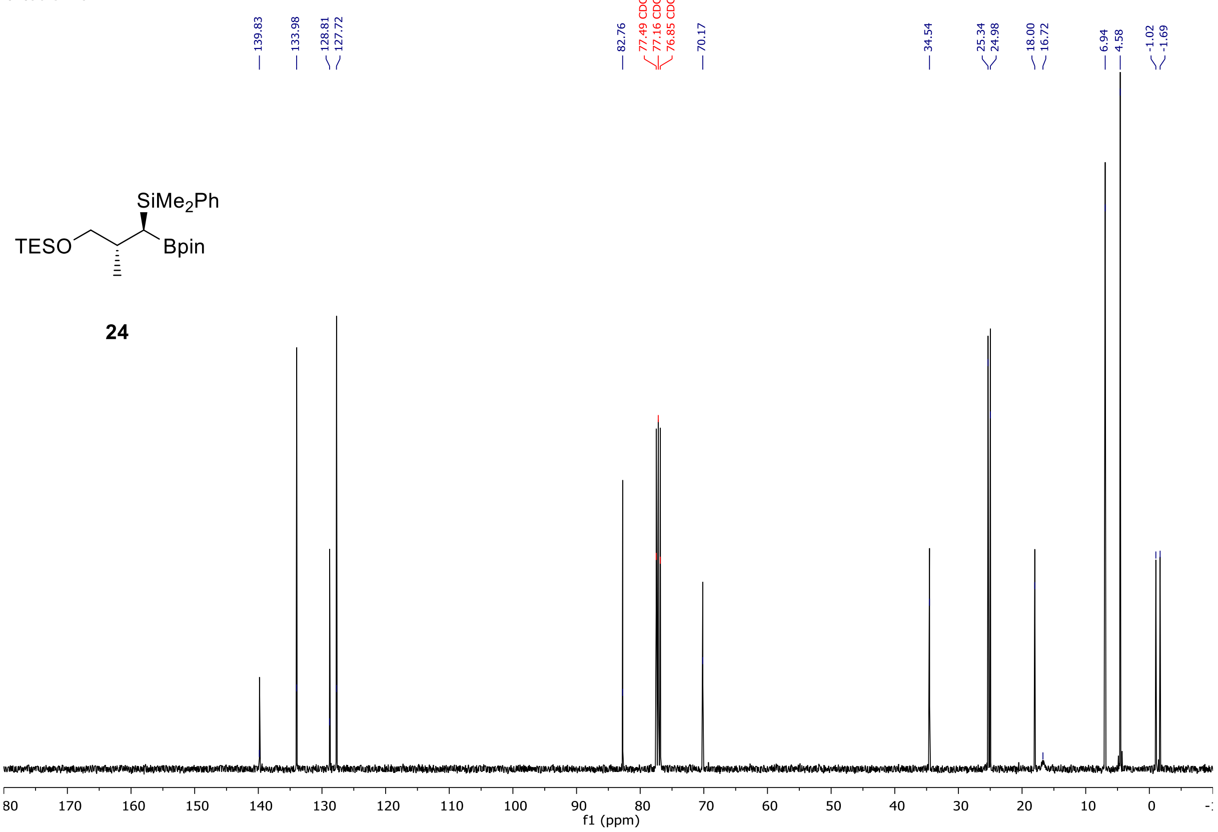

^{1}H NMR (400 MHz, $CDCl_3$) of **SI-7** (*see procedure*)

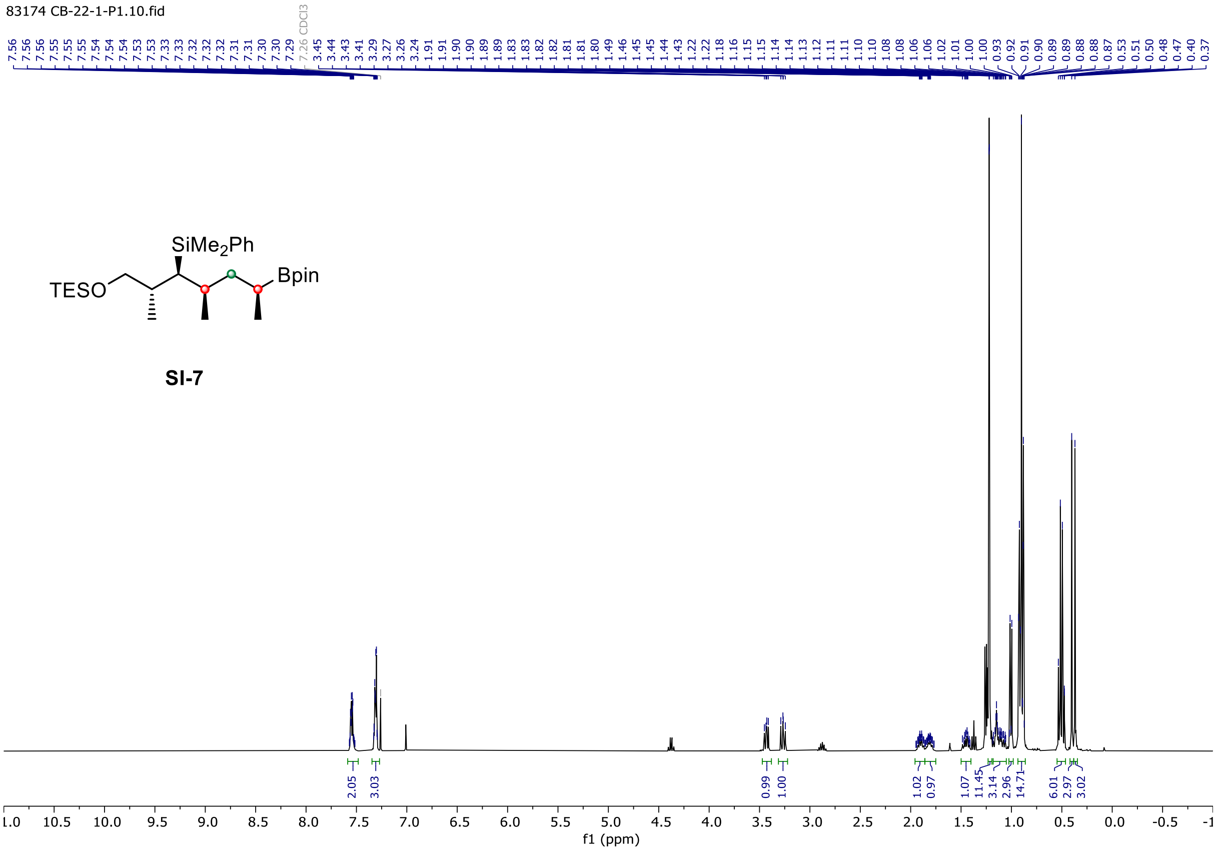

^{13}C NMR (101 MHz, $CDCl_3$) of **SI-7**

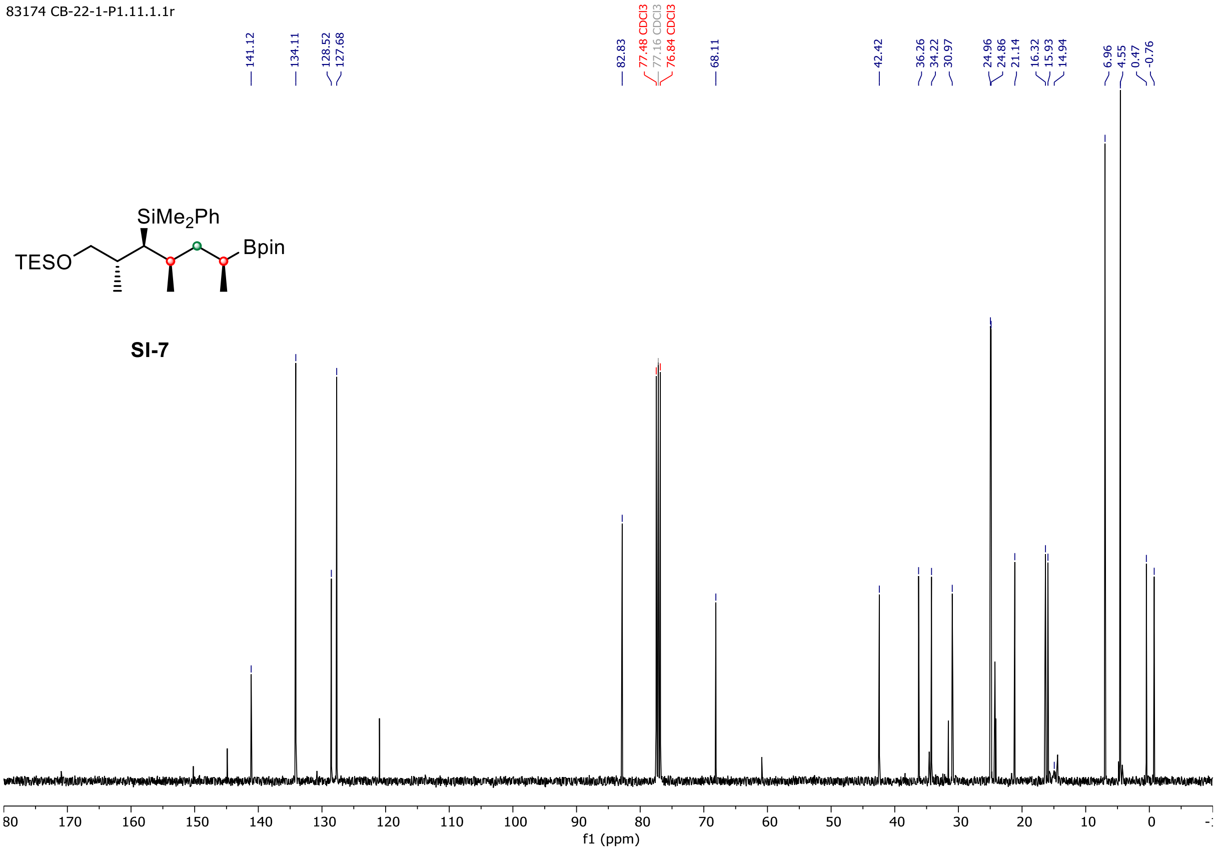

^{1}H NMR (500 MHz, $CDCl_3$) of **29** (see procedure)

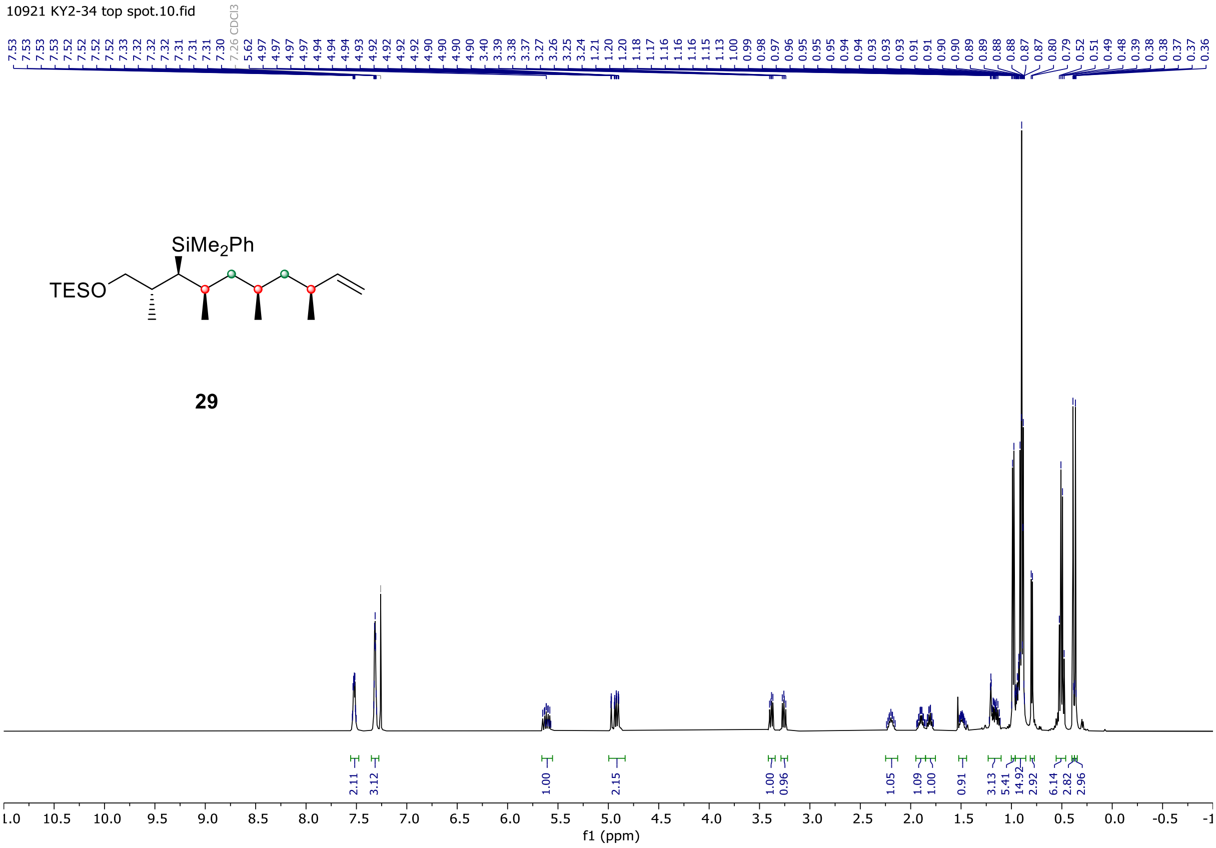

^{13}C NMR (126 MHz, $CDCl_3$) of **29**

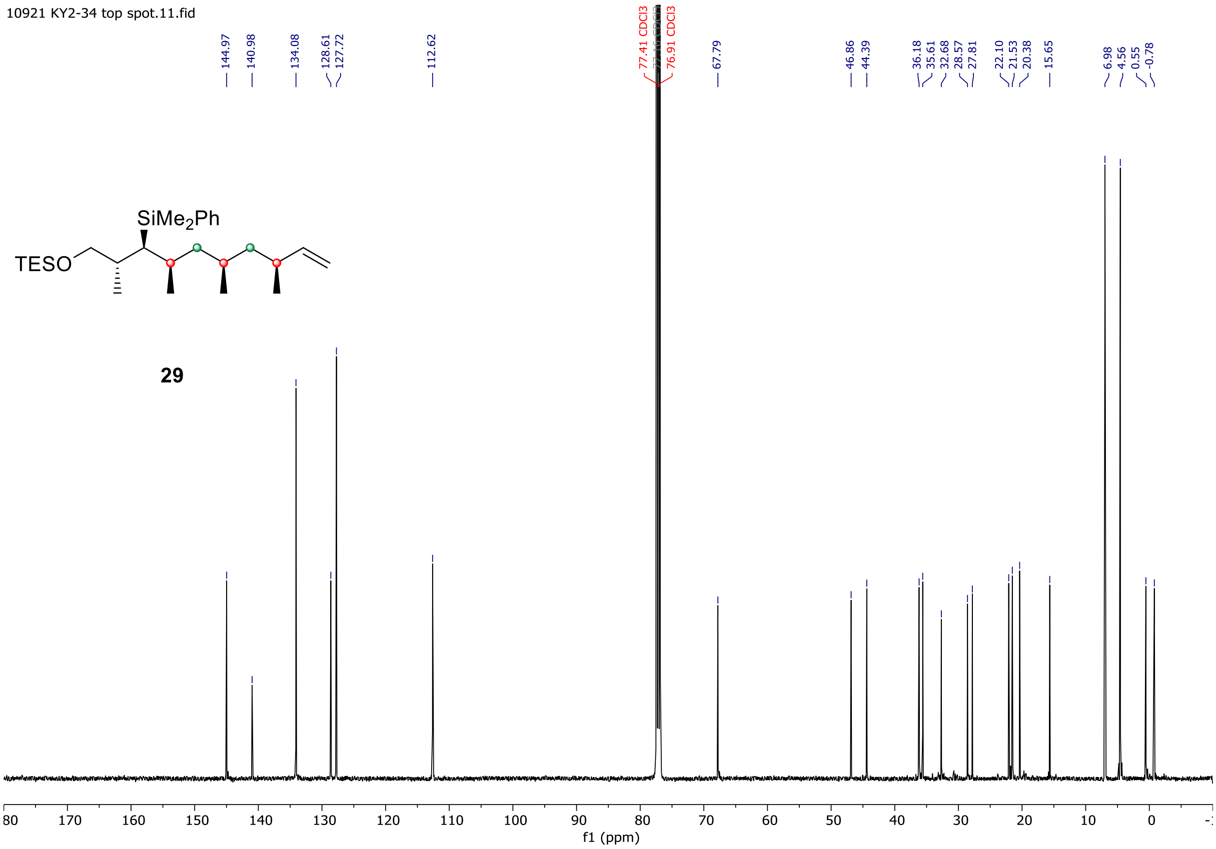

^{1}H NMR (500 MHz, CD_3OD) of **30** (*see procedure*)

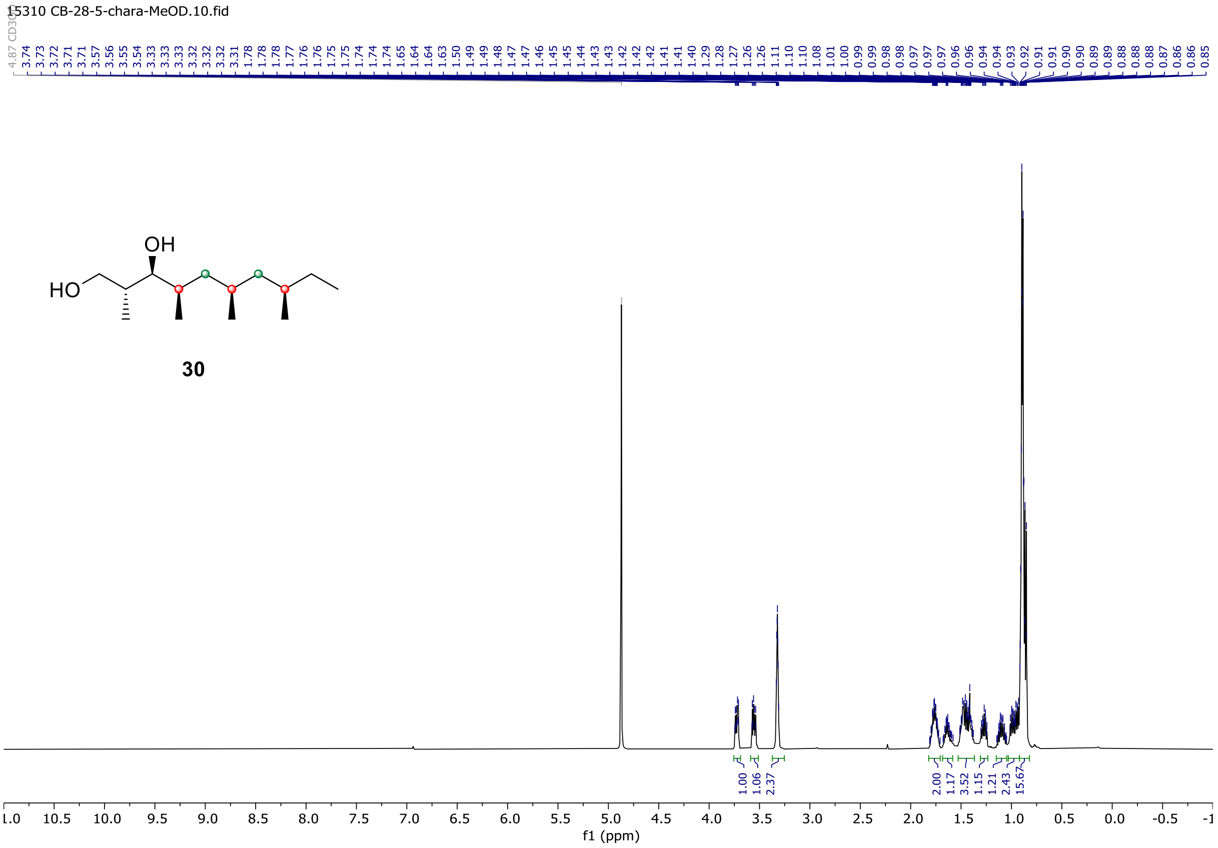

^{13}C NMR (126 MHz, CD_3OD) of **30**

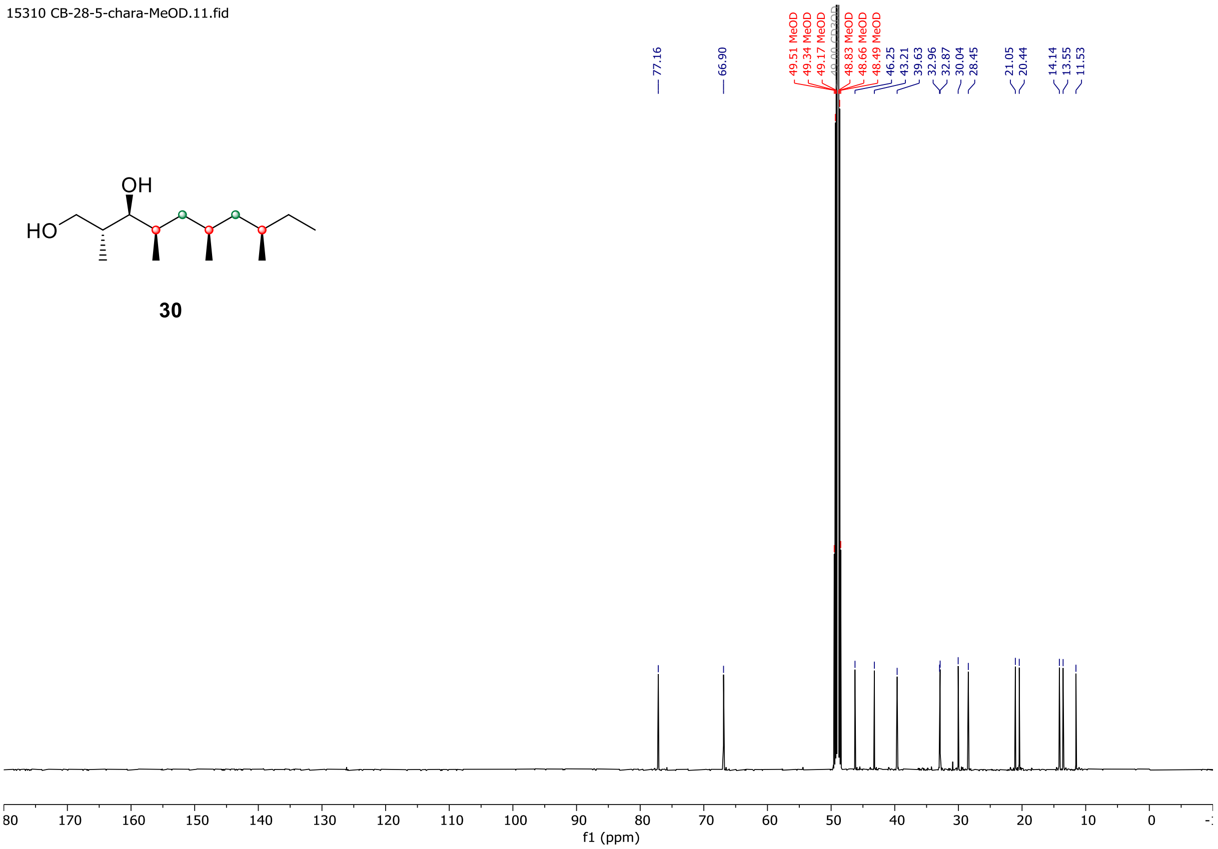

^{1}H NMR (400 MHz, $CDCl_3$) of **31** (*see procedure*)

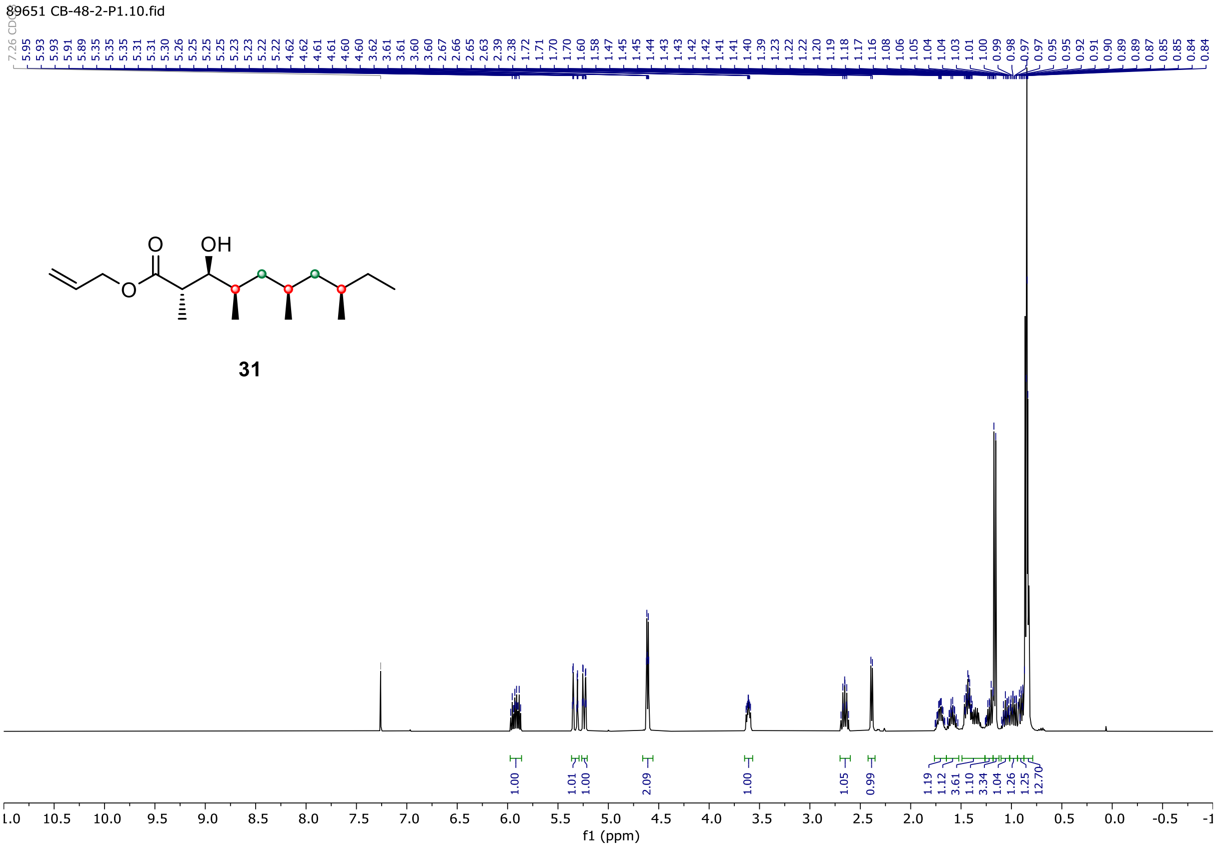

^{13}C NMR (101 MHz, $CDCl_3$) of **31**

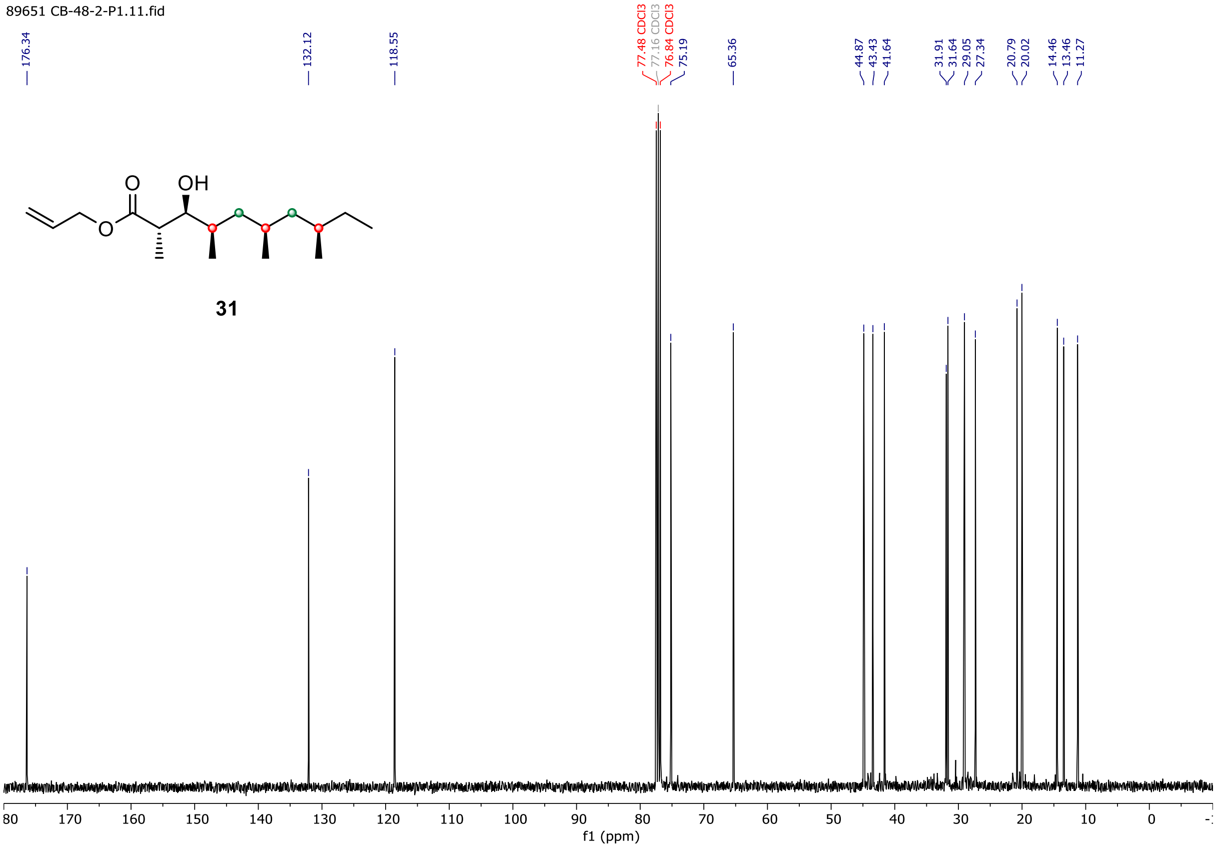

^{1}H NMR (400 MHz, $CDCl_3$) of **SI-12** (*see procedure*)

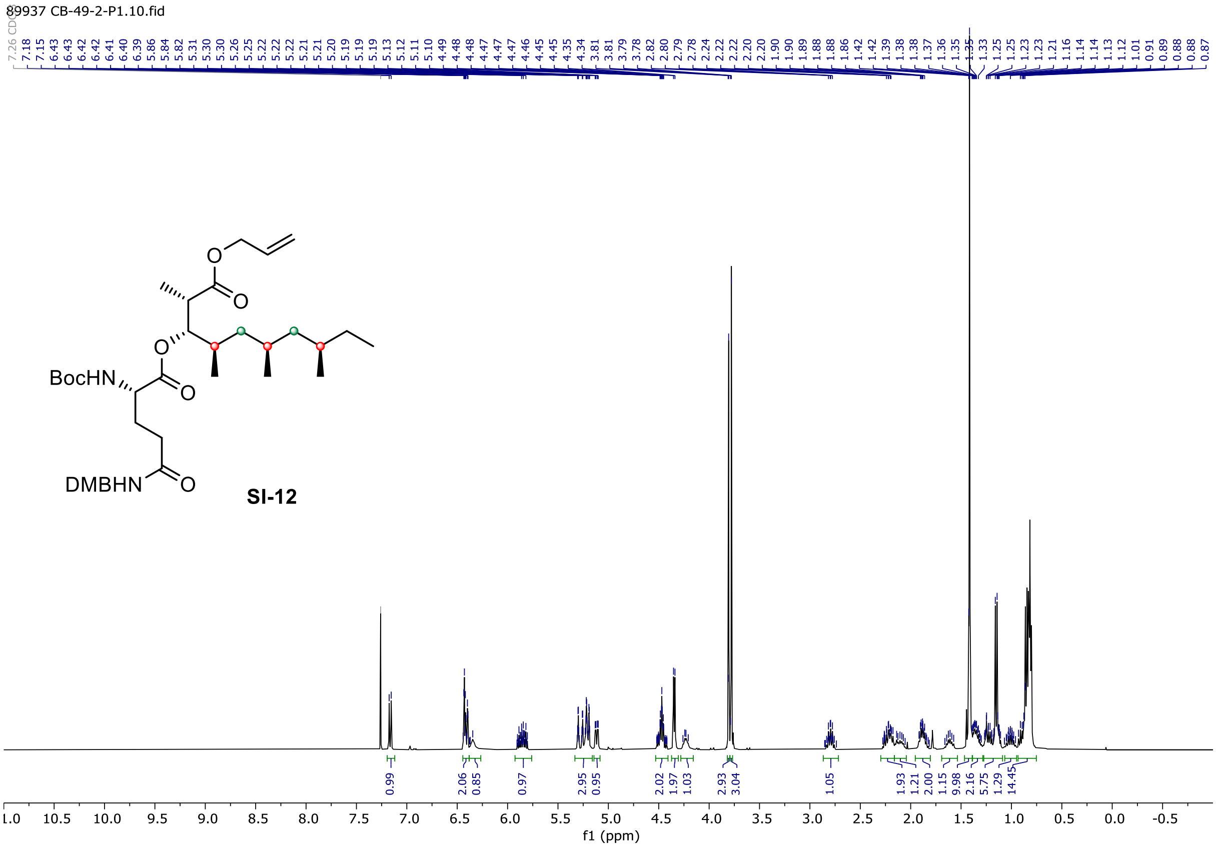

^{13}C NMR (101 MHz, $CDCl_3$) of **SI-12**

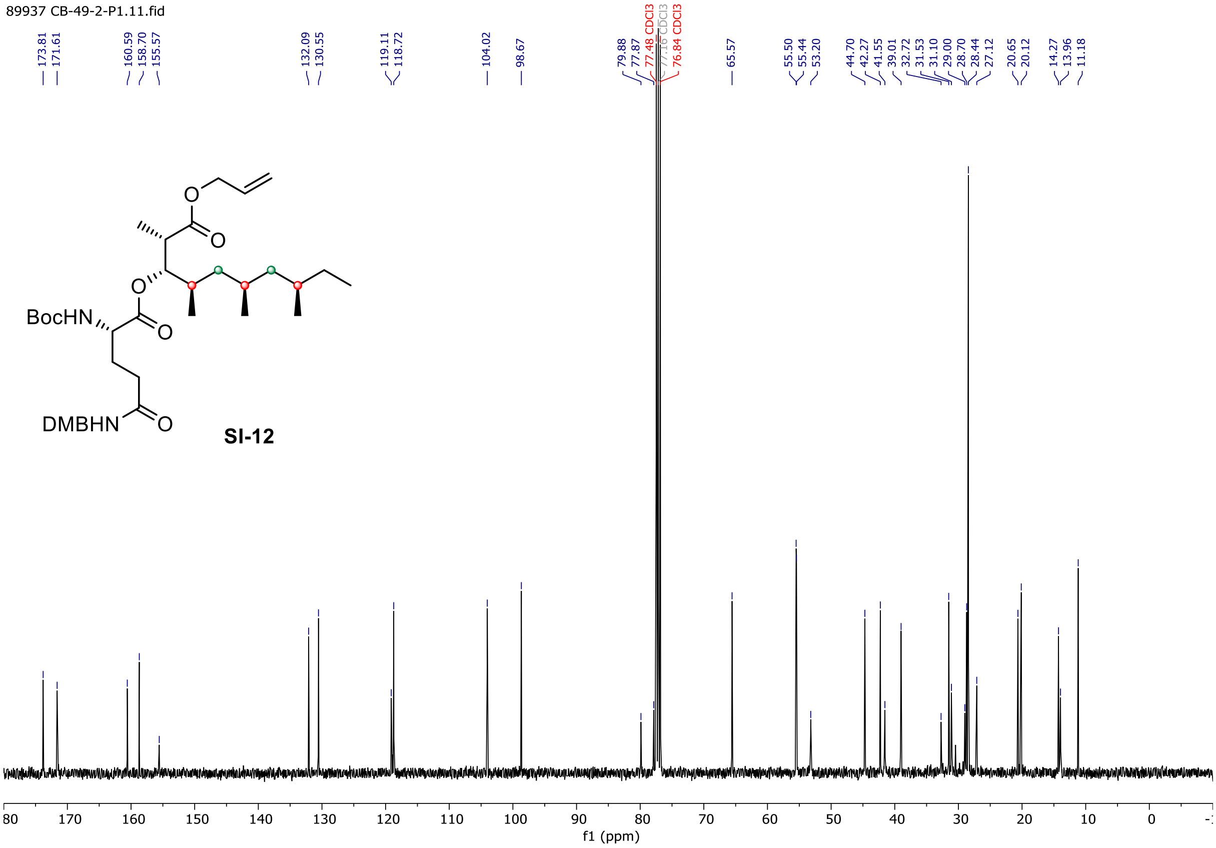

^{1}H NMR (500 MHz, CD_3OD) of **34** (*see procedure*)

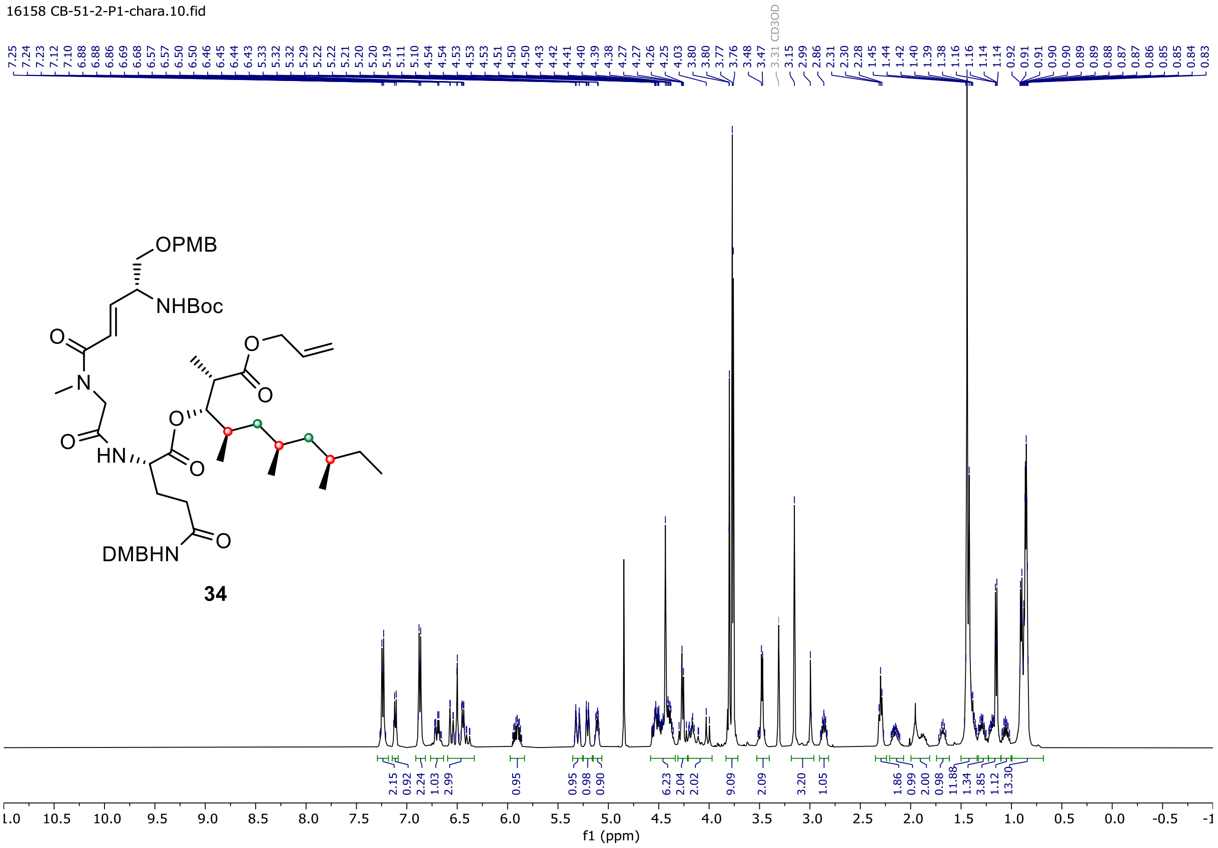

34

^{13}C NMR (126 MHz, CD_3OD) of **34**

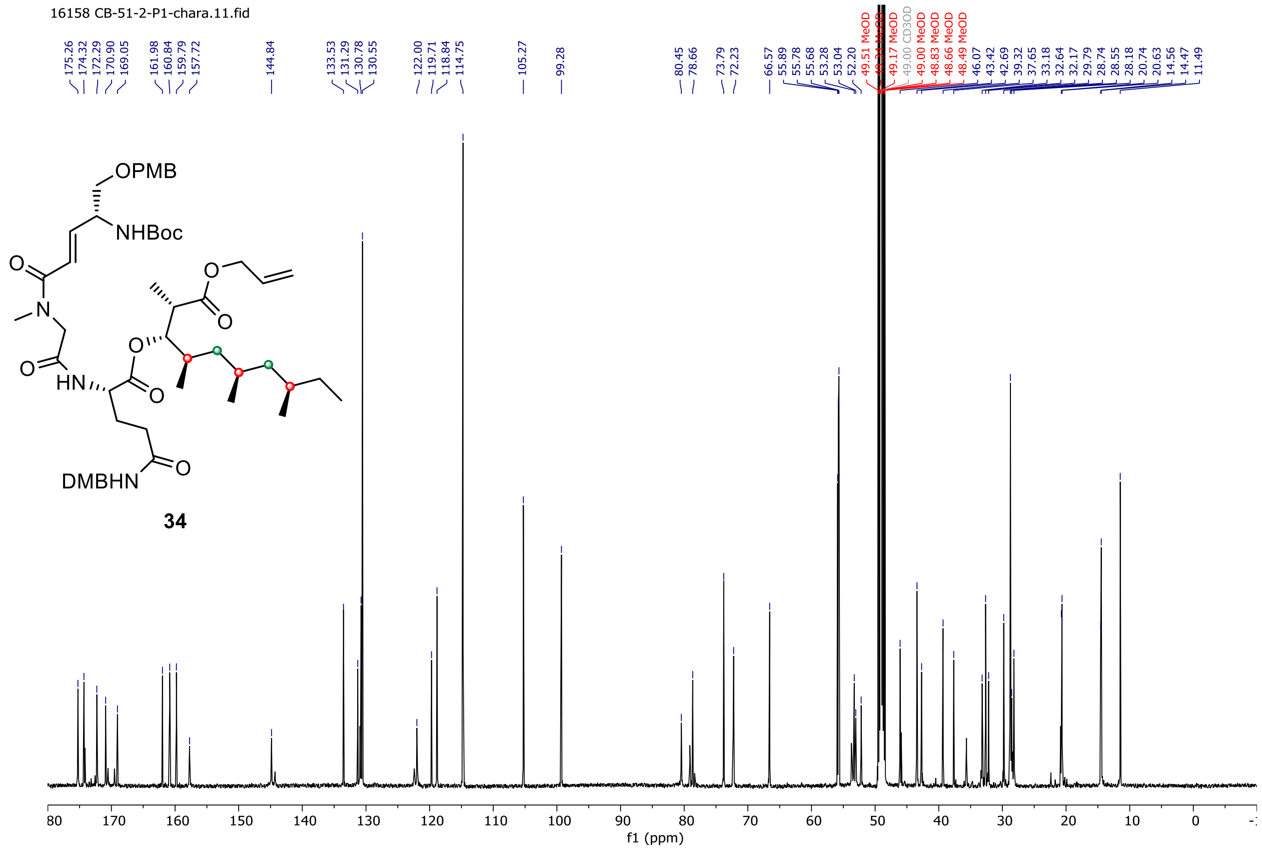

34

^{1}H NMR (500 MHz, CD_3OD) of **SI-13** (*see procedure*)

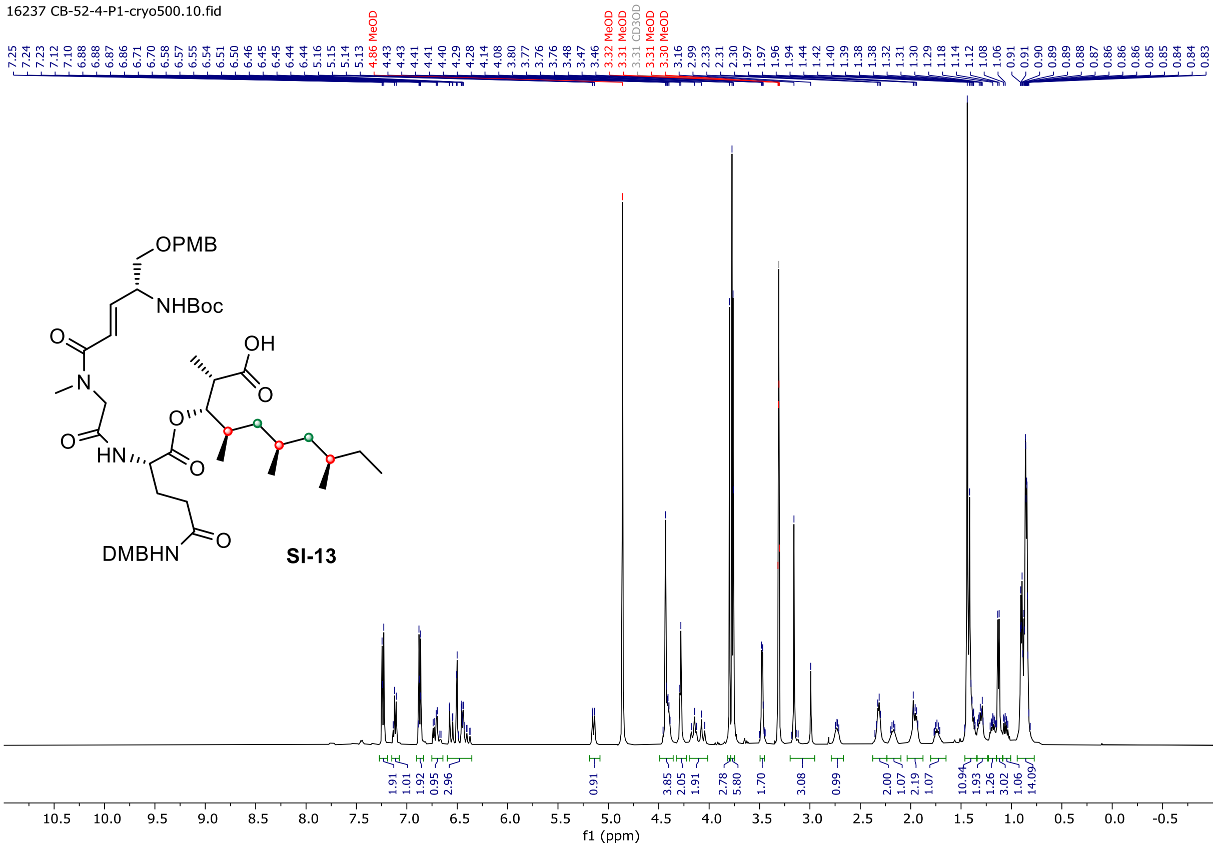

^{13}C NMR (126 MHz, CD_3OD) of **SI-13**

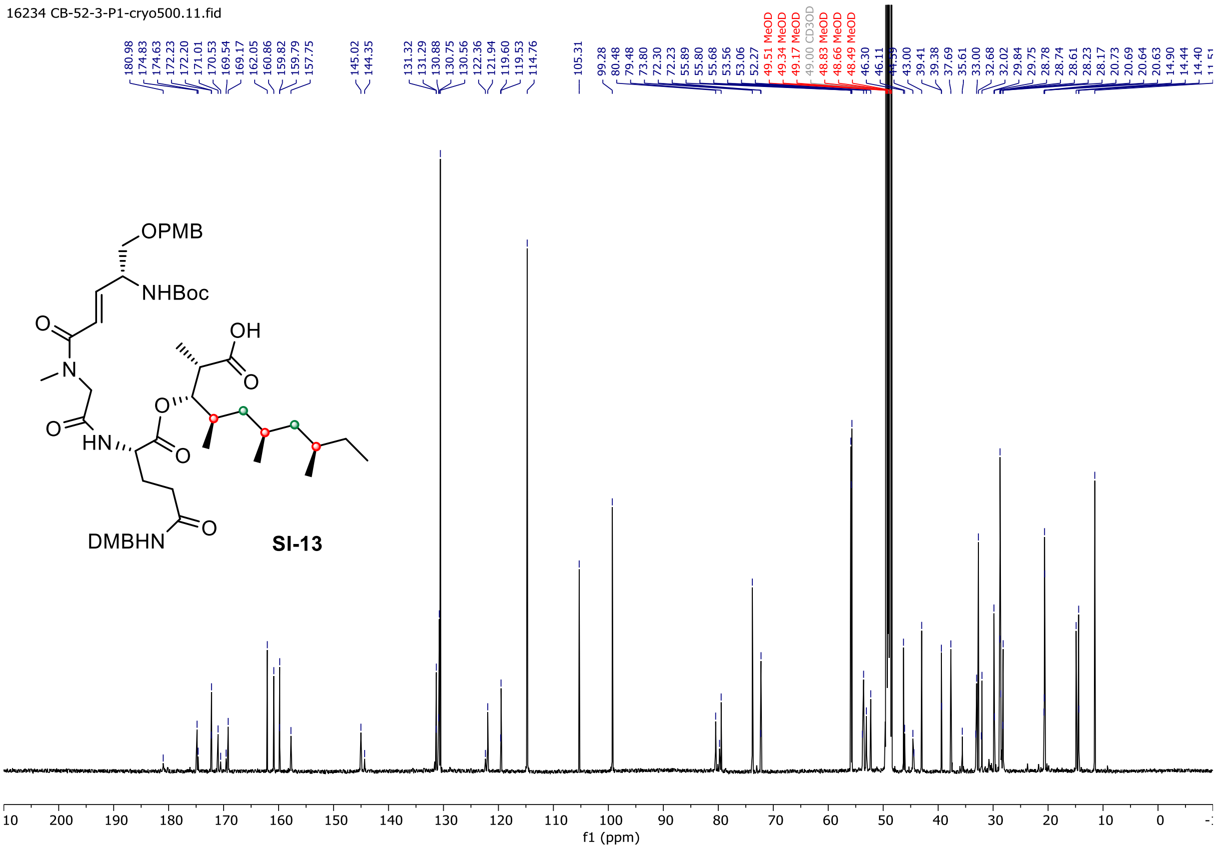

^{1}H NMR (500 MHz, DMSO-d_6) of **36** (*see procedure*)

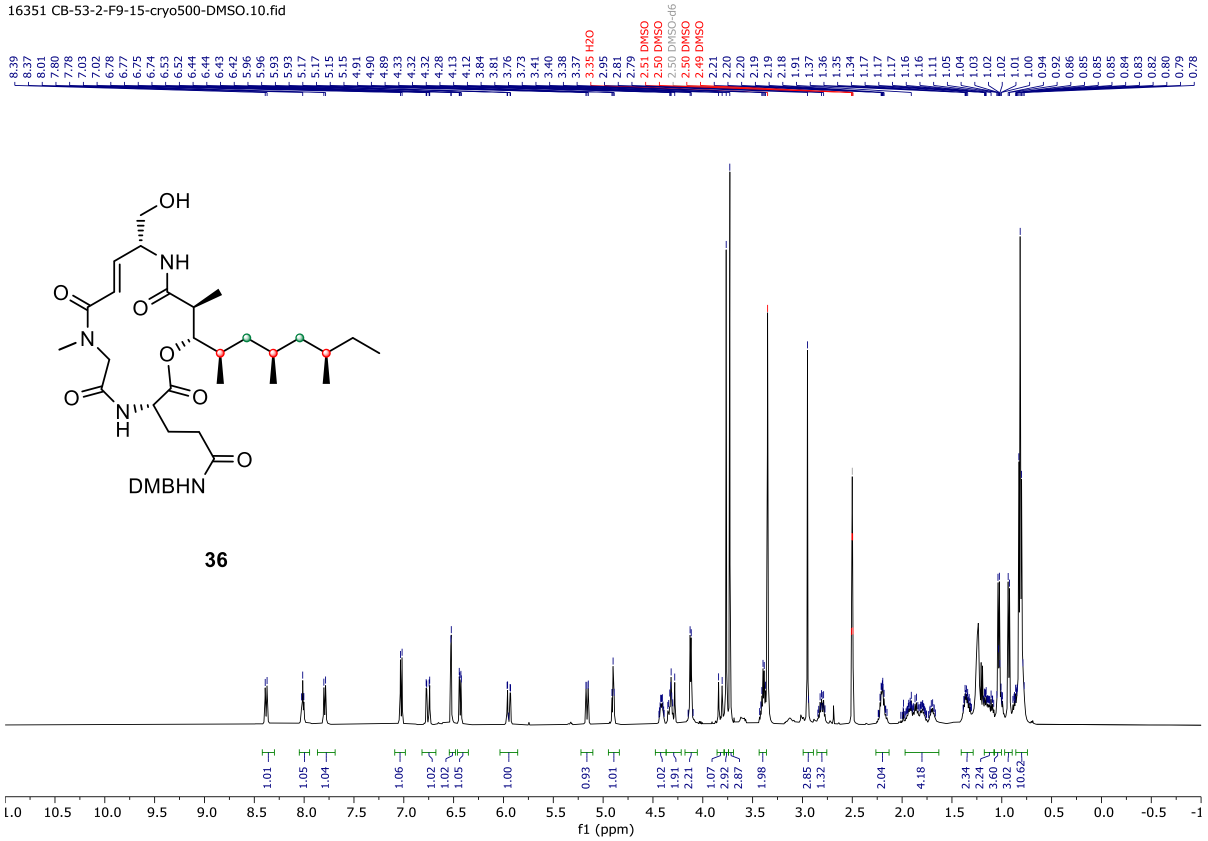

^{13}C NMR (126 MHz, DMSO-d_6) of **36**

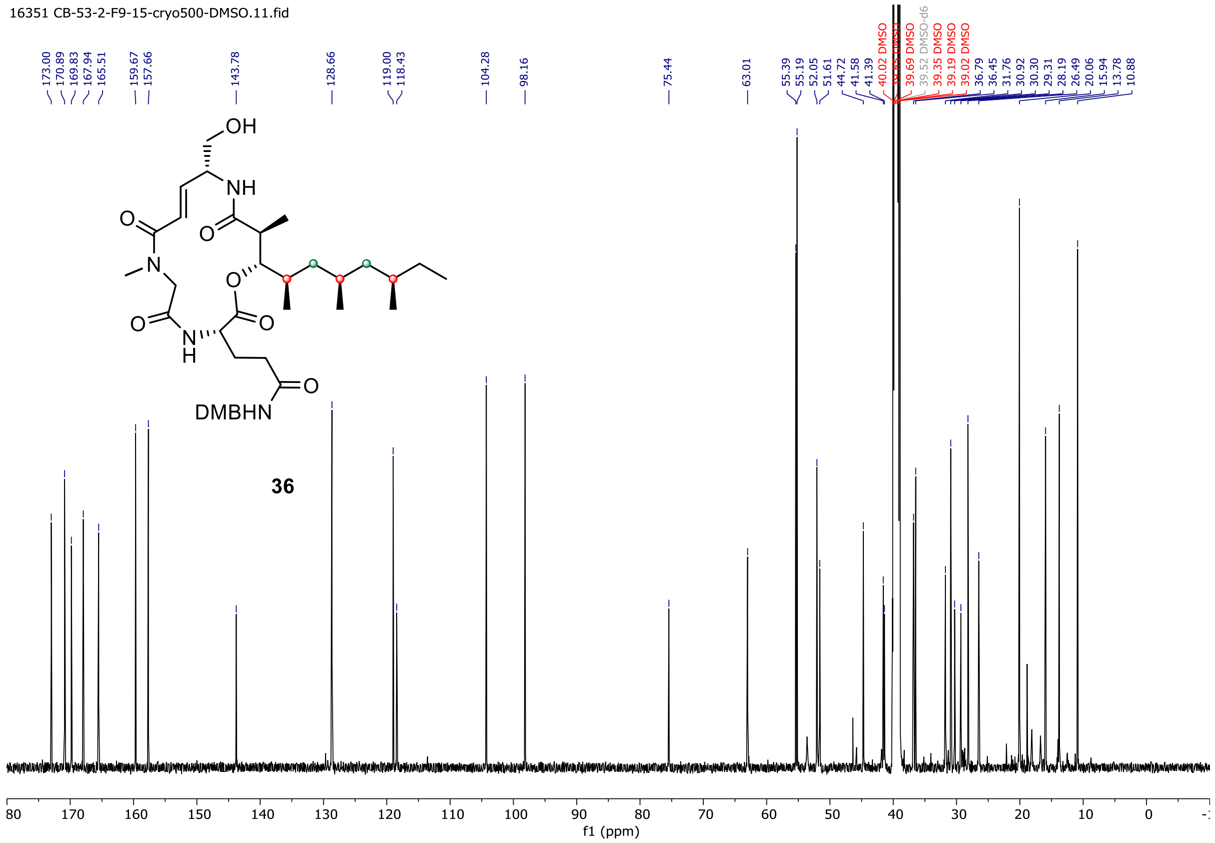

^{1}H NMR (500 MHz, CD_3OD) of (–)-rakicidin F (**3**) (*see procedure*)

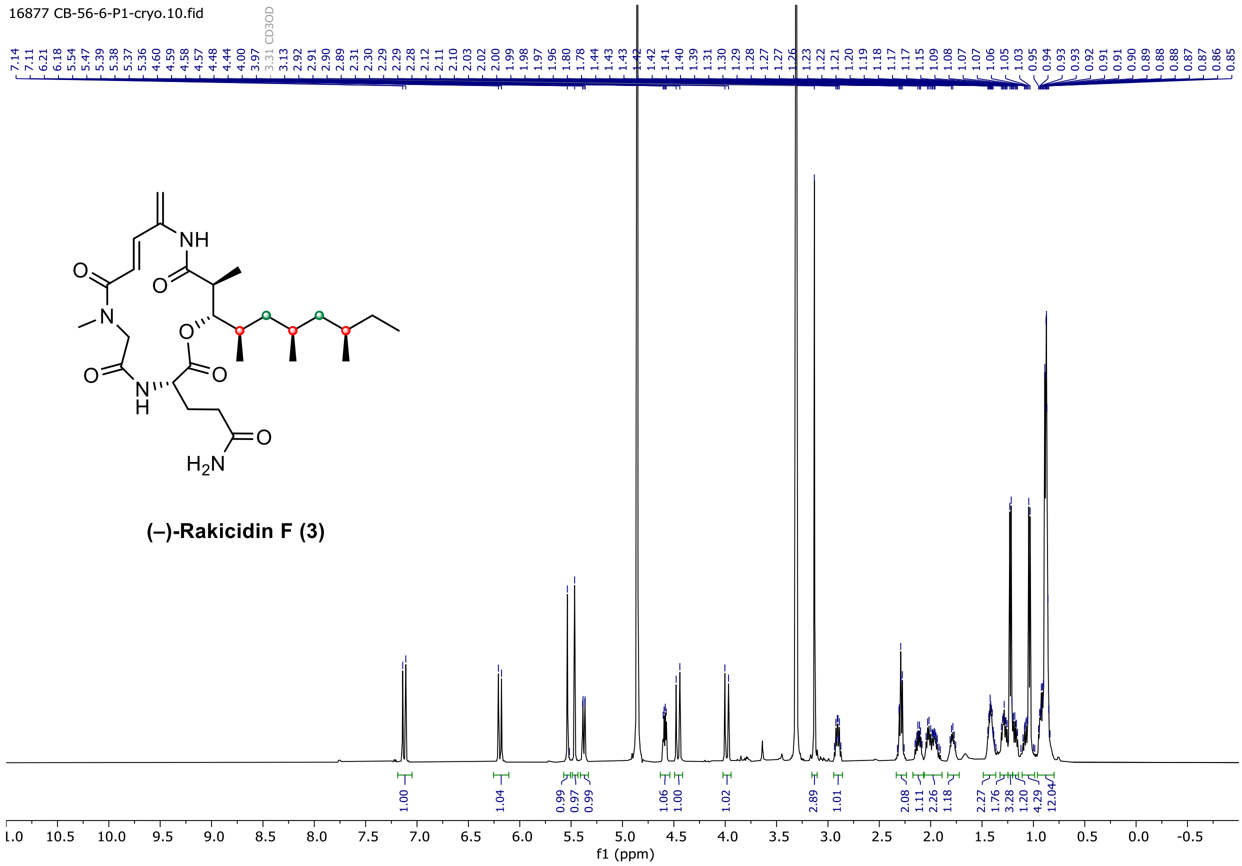

^{13}C NMR (126 MHz, CD_3OD) of (–)-rakicidin F (**3**)

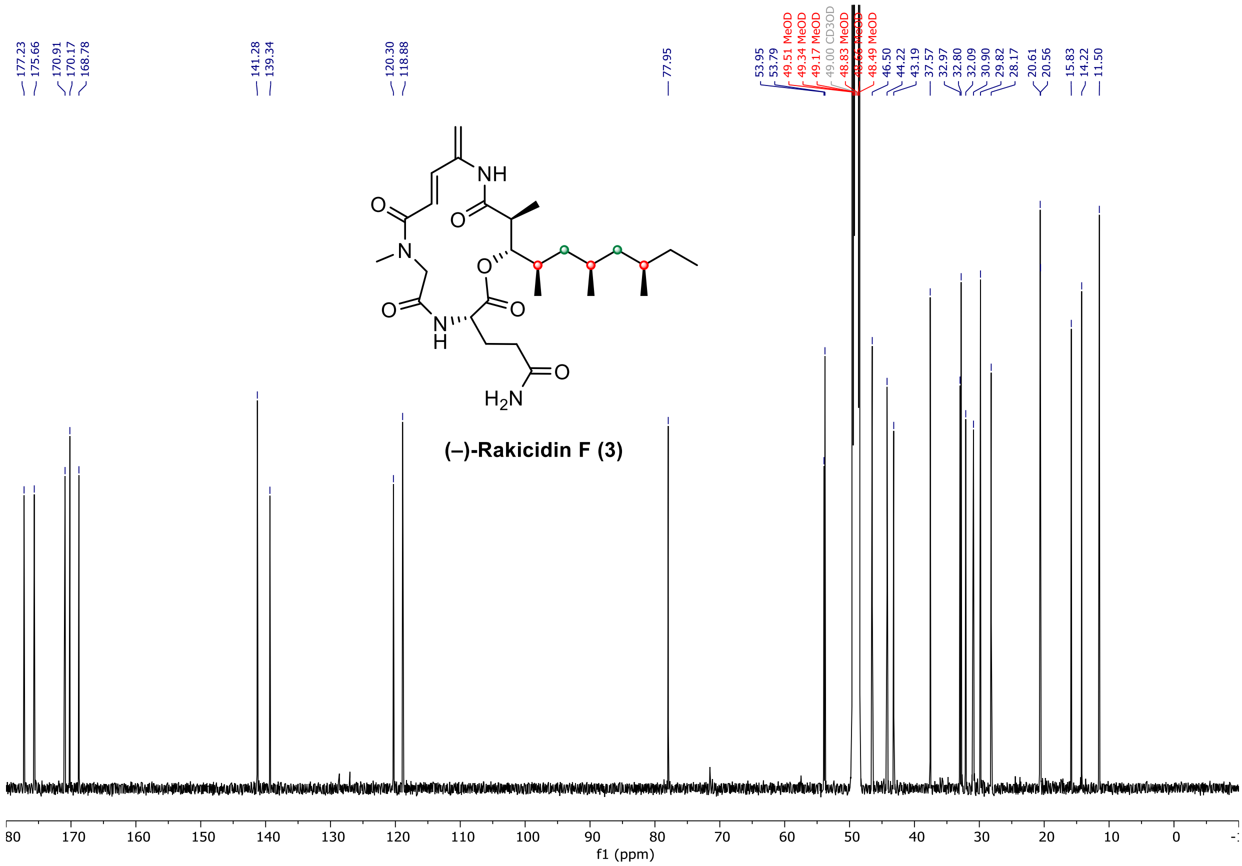

^{1}H NMR Spectrum Comparison of crude (with ISTD (benzene)) and pure (–)-Rakicidin F (**3**) in CD_3OD

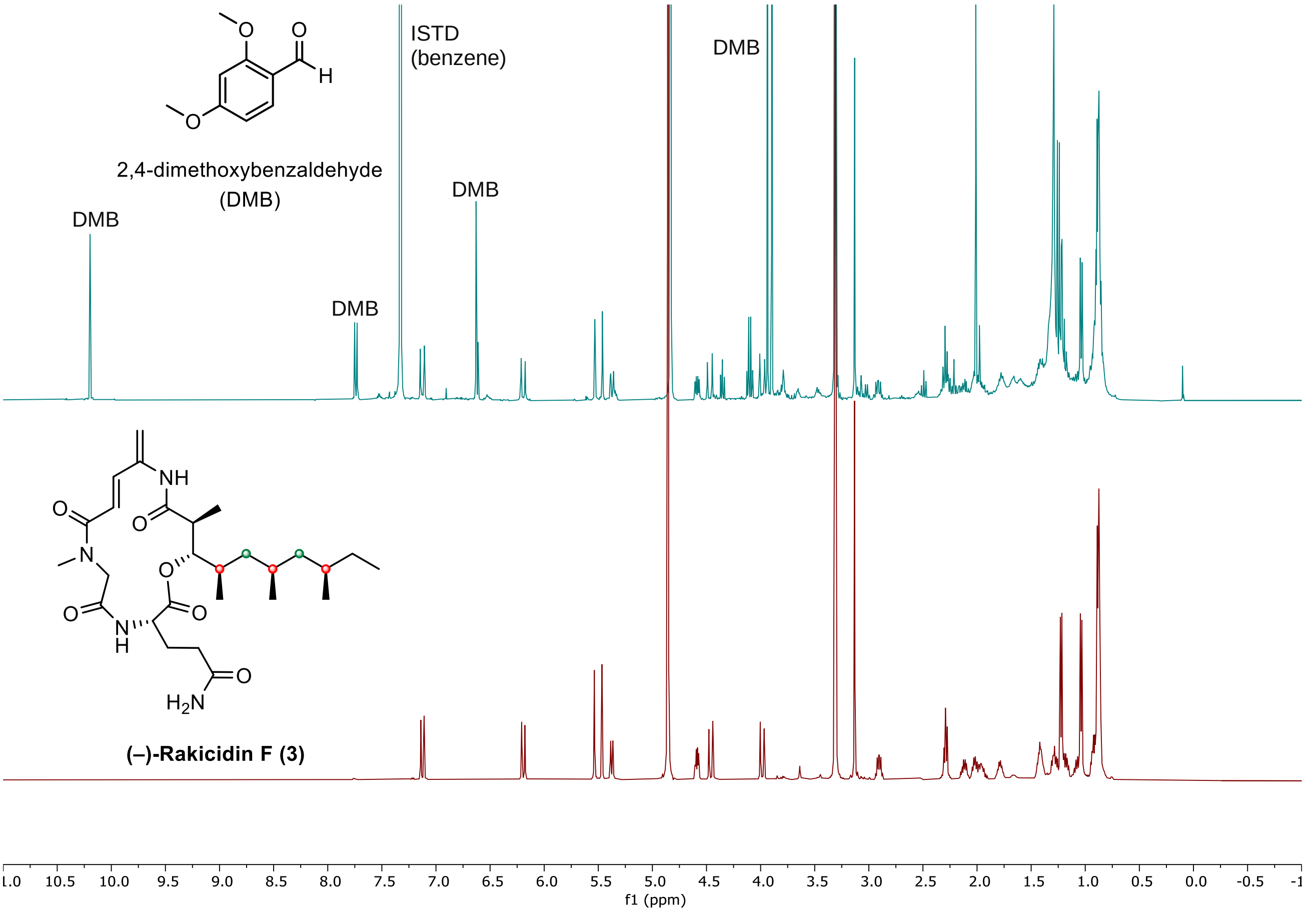

COSY of (–)-Rakicidin F (**3**)

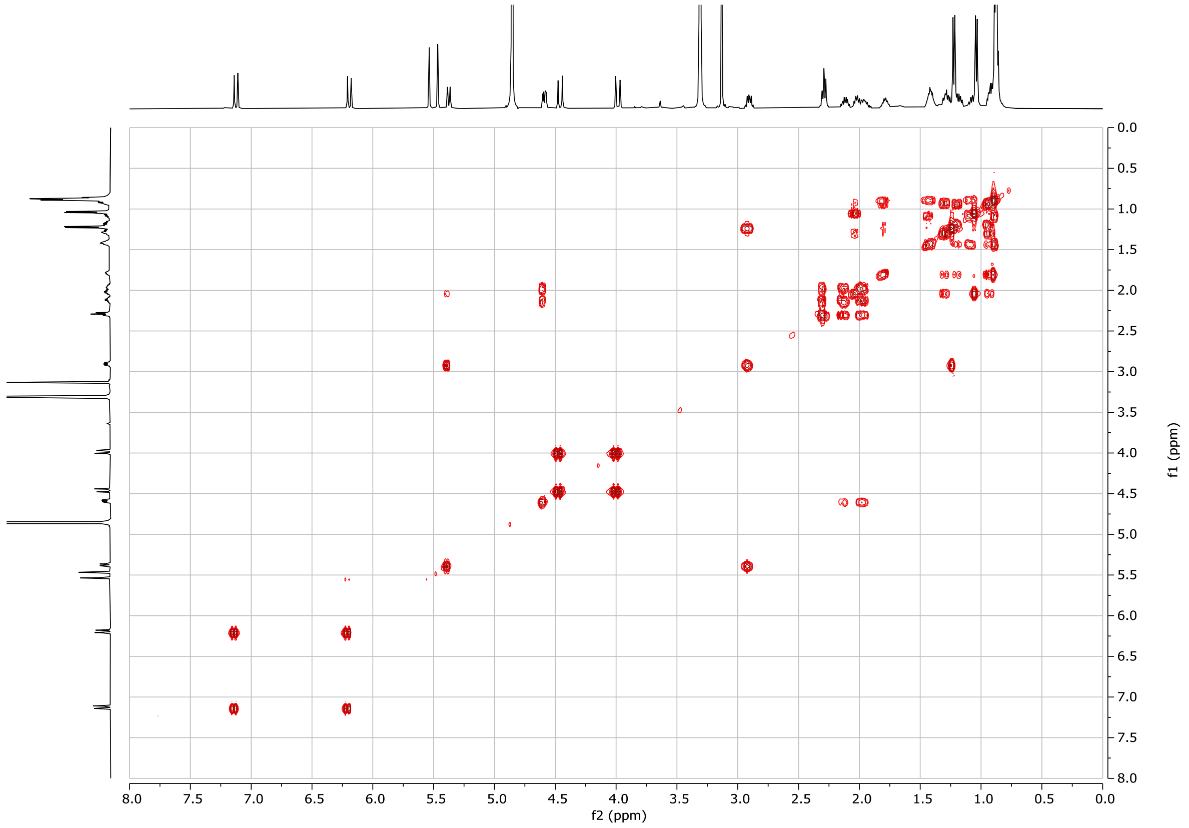

HSQC of (–)-Rakicidin F (**3**)

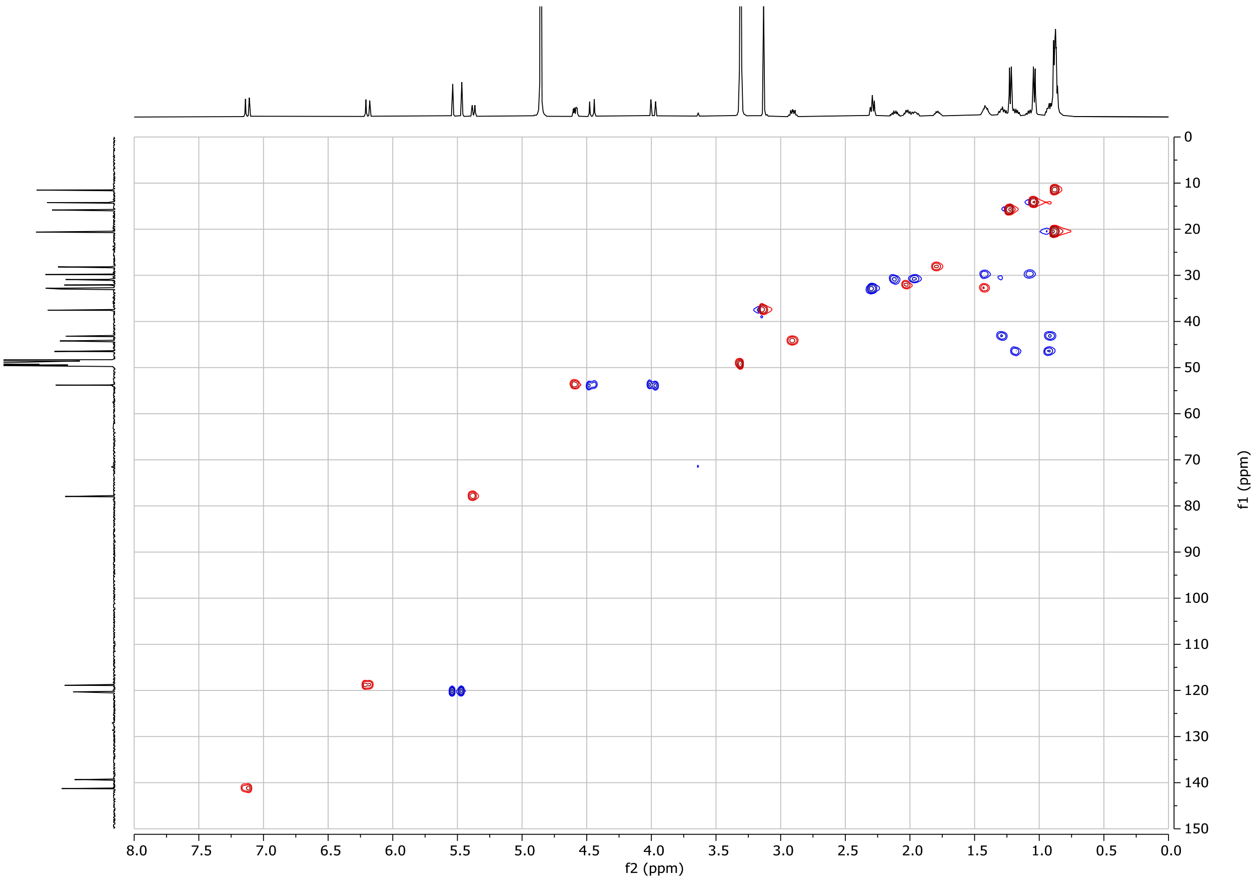

HMBC of (–)-Rakicidin F (**3**)

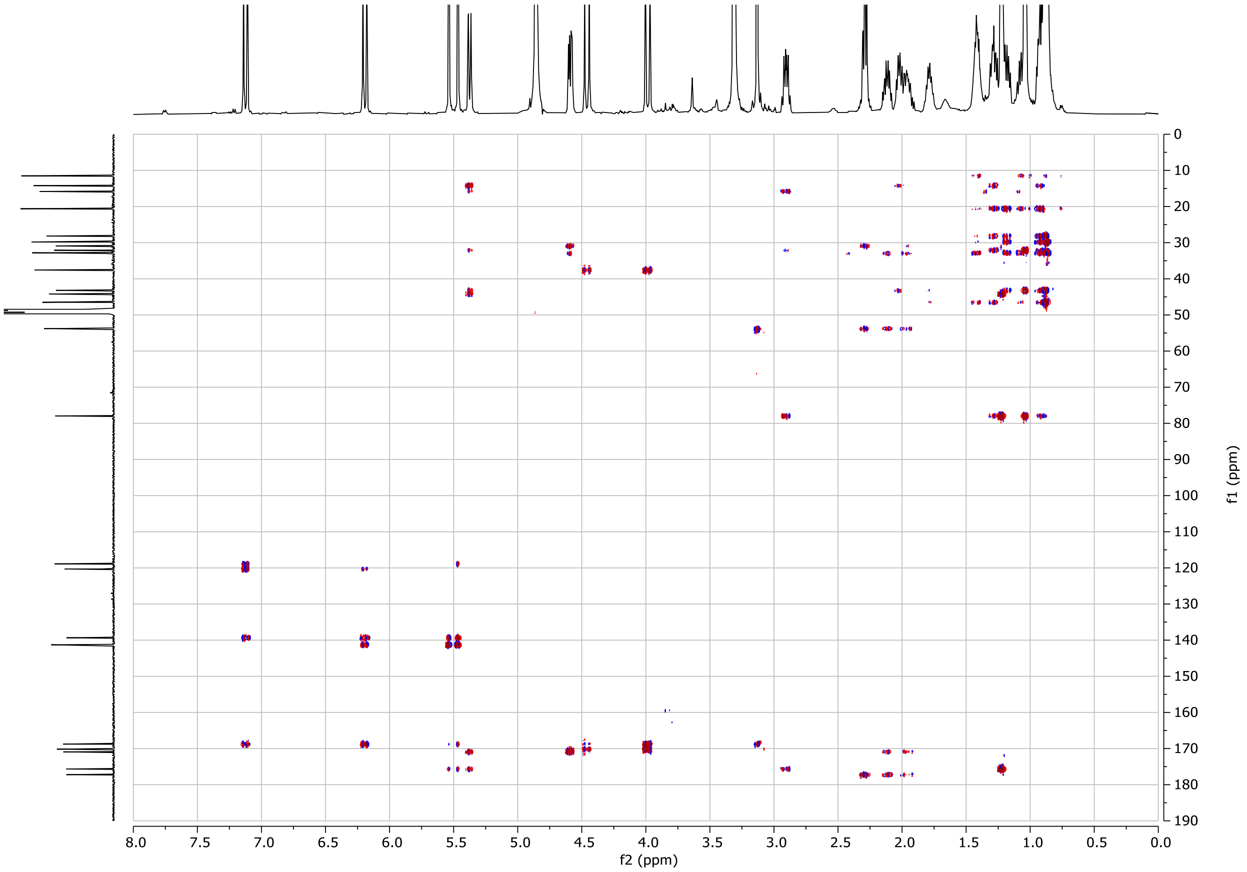

HRMS Spectrum of (–)-Rakicidin F (**3**)

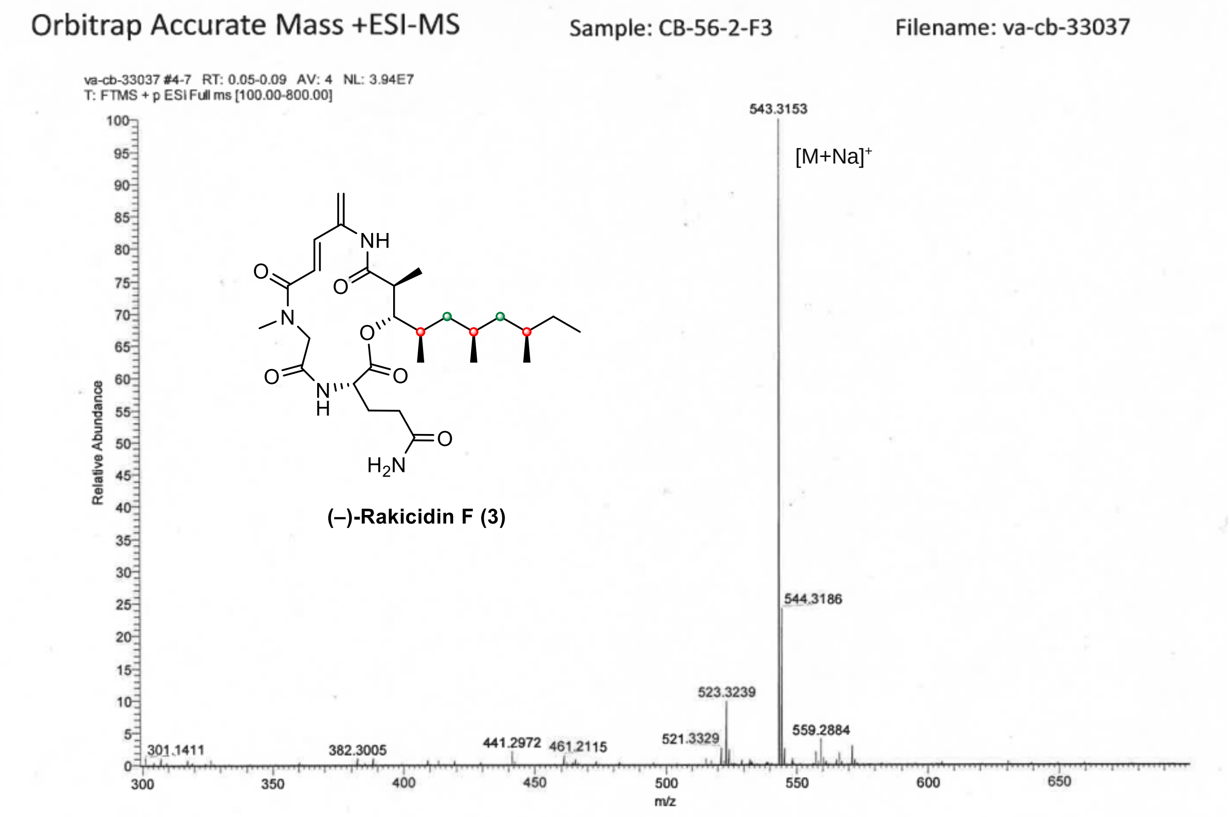

Observed m/z	Theoretical Mass	Error (ppm)	Formula	ID
543.3153	543.3153	0.00	$C_{27}H_{44}O_6N_4$	$[M+Na]^+$

IR Spectrum of (–)-Rakicidin F (**3**)

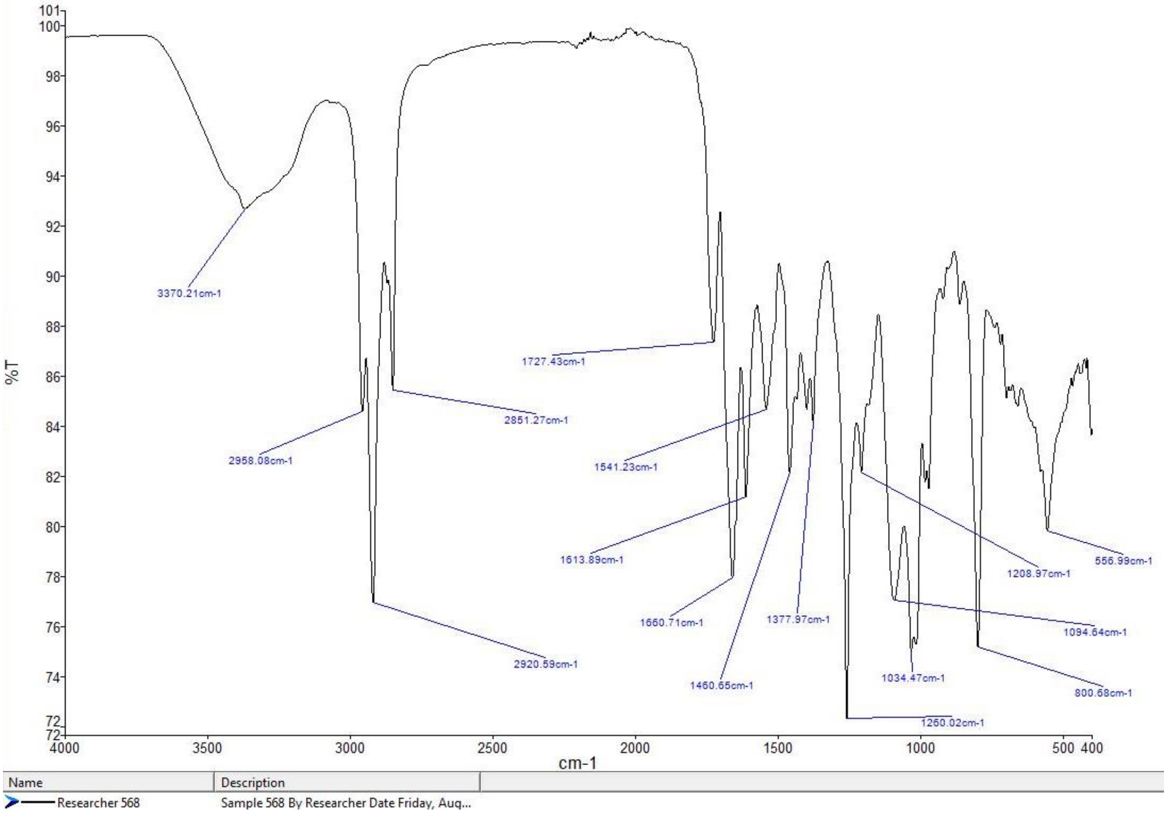

4. REFERENCES

1) A. F. Burchat, J. M. Chong, N. Nielsen, *J. Organomet. Chem.* **1997**, *542*, 281-283.

2) M. Tsakos, L. L. Clement, E. S. Schaffert, F. N. Olsen, S. Rupiani, R. Djurhuus, W. Yu, K. M. Jacobsen, N. L. Villadsen, T. B. Poulsen, *Angew. Chem. Int. Ed.* **2016**, *55*, 1030-1035.

3) M. Burns, S. Essafi, J. R. Bame, S. P. Bull, M. P. Webster, S. Balieu, J. W. Dale, C. P. Butts, J. N. Harvey, V. K. Aggarwal, *Nature* **2014**, *513*, 183-188.

4) S. Kitani, T. Ueguchi, Y. Igarashi, K. Leetanasaksakul, A. Thamchaipenet, T. Nihira, *J. Antibiot.* **2018**, *71*, 139-141.

5) M. A. Blanchette, W. Choy, J. T. Davis, A. P. Essenfeld, S. Masamune, W. R. Roush, T. Sakai, *Tetrahedron Lett.* **1984**, *25*, 2183-2186.

6) I. Paterson, K.-S. Yeung, J. B. Smaill, *Synlett* **1993**, *10*, 774-776.
